# Novel eukaryotic enzymes modifying cell-surface biopolymers

**DOI:** 10.1186/1745-6150-5-1

**Published:** 2010-01-07

**Authors:** Vivek Anantharaman, L Aravind

**Affiliations:** 1 National Center for Biotechnology Information, National Library of Medicine, National Institutes of Health, Bethesda, MD 20894, USA

## Abstract

**Background:**

Eukaryotic extracellular matrices such as proteoglycans, sclerotinized structures, mucus, external tests, capsules, cell walls and waxes contain highly modified proteins, glycans and other composite biopolymers. Using comparative genomics and sequence profile analysis we identify several novel enzymes that could be potentially involved in the modification of cell-surface glycans or glycoproteins.

**Results:**

Using sequence analysis and conservation we define the acyltransferase domain prototyped by the fungal Cas1p proteins, identify its active site residues and unify them to the superfamily of classical 10TM acyltransferases (e.g. oatA). We also identify a novel family of esterases (prototyped by the previously uncharacterized N-terminal domain of Cas1p) that have a similar fold as the SGNH/GDSL esterases but differ from them in their conservation pattern.

**Conclusions:**

We posit that the combined action of the acyltransferase and esterase domain plays an important role in controlling the acylation levels of glycans and thereby regulates their physico-chemical properties such as hygroscopicity, resistance to enzymatic hydrolysis and physical strength. We present evidence that the action of these novel enzymes on glycans might play an important role in host-pathogen interaction of plants, fungi and metazoans. We present evidence that in plants (e.g. PMR5 and ESK1) the regulation of carbohydrate acylation by these acylesterases might also play an important role in regulation of transpiration and stress resistance. We also identify a subfamily of these esterases in metazoans (e.g. C7orf58), which are fused to an ATP-grasp amino acid ligase domain that is predicted to catalyze, in certain animals, modification of cell surface polymers by amino acid or peptides.

**Reviewers:**

This article was reviewed by Gaspar Jekely and Frank Eisenhaber

## Findings

Eukaryotes display a rich complement of secreted and membrane-anchored cell-surface proteins, whose amino acid side-chains are subject to numerous post-translational modifications. These modifications include addition of extensive polysaccharide moieties to asparagine or serine/threonine side chains (N and O linked glycosylation respectively), sulfatation, hydroxylation and cross-linking. These modified surface proteins, together with other biopolymers such as polysaccharides, which might also be highly modified and cross-linked, constitute diverse organic matrices of eukaryotes [1]. The matrices include proteoglycans, sclerotinized structures and mucus in animals, external tests and capsules of various microbial eukaryotes, and cell walls and waxes of plants and fungi. Not only do these play a major structural role in both unicellular and multicellular eukaryotes, but they are also important in the defense against parasites or interactions of parasites with their hosts. The enzymatic components of the two major eukaryotic glycosylation systems, as well as enzymes catalyzing reactions like hydroxylation of prolines and lysines, and sulfatation of side chains such as tyrosine are reasonably well understood [2]. Recently, several studies on animal developmental pathways such as the Notch, Wnt and Hedgehog have uncovered enzymes that catalyze other modifications such as acylation of proteins by long chain fatty acids and novel glycosylations catalyzed by enzymes of the fringe and fucosyltransferase families [3]. Furthermore, the analysis of eukaryotic genomes reveals a large complement of secreted biopolymer-modifying enzymes that remain enigmatic in terms of their biochemical role. These observations suggested that there might be potentially uncharacterized modifications of eukaryotic cell surface proteins and glycans, which might contribute to their structural properties and interactions.

Given the profound importance of such cell surface modifying enzymes to host-pathogen interactions, evasion of host-surveillance and developmental processes, we were interested in identifying novel families of such enzymes and computationally predicting their function to facilitate their future exploration by experimental means. In this study we used sensitive sequence and structure analysis methods to investigate eukaryotic proteins with a potential role in cell surface modifications and identified novel enzymes that contain different catalytic domains such as: 1) an esterase with an α/β fold similar to that of the GDSL/SGNH superfamily of esterases, albeit with somewhat distinct active sites; 2) membrane-associated acyltransferases; 3) ATP-grasp enzymes with potential peptide ligase activity. Based on the available genetic data, catalytic site configuration and contextual information we predict that these proteins catalyze previously uncharacterized modifications of surface proteins in various biological roles.

### Analysis of the Cas1p protein

As part of an effort to uncover previously uncharacterized eukaryotic enzymes modifying secreted and cell-surface proteins we investigated the Cas1p protein of the pathogenic fungus *Cryptococcus neoformans*, which is required for the synthesis of O-acetylated glucuronoxylomannans (GXM), the primary capsular constituent of this fungus [4]. This acetylated capsule is critical for the virulence of *Cryptococcus *in animal hosts. Interestingly, homologs of this protein were also found in animals and plants suggesting that such modifications might have a more general function in cell-surface adhesion across eukaryotes. Evidence from *Cryptococcus *indicates that the multi-TM protein Cas1p is the acyltransferase that adds the acetyl groups to the O-6 position of the mannose residues of the backbone of the GXM [4]. Hence, the conserved multi-TM domain shared by Cas1p and its animal and plant homologs has been termed the Cas1p acetyltransferase domain (e.g. see PFAM PF07779). However, it should be noted that this region has not been unified with any previously known acyltransferase domain. Further, it was originally claimed that Cas1p shows similarities to multi-TM glycosyltransferases, though no evidence from sequence profile searches has been provided to show that it is related to any known glycosyltransferase [4]. Additionally, Cas1p contains a previously uncharacterized N-terminal globular domain that is conserved in its animal homologs, but not those from plants. This observation, taken together with the unclear affinities of the multi-TM domain, indicated that the catalytic properties of the Cas1p protein were poorly understood. Given that both the TM and the N-terminal globular domain are more widely distributed, we systematically analyzed them using sequence profile searches to better characterize their functions and phyletic spread.

### The multi-TM domain of Cas1p is unified with the classical 10TM sugar acyltransferase superfamily

To identify the affinities of the multi-TM domain in Cas1p, we initiated PSI-BLAST searches of the non-redundant database (expect (e)-value for inclusion in threshold = .01, iterated to convergence) with this region from the *Cryptococcus neoformans *protein Cas1p (gi: 74681762; region 370-960). The search initially recovers known homologs of the Cas1p family from other eukaryotes. In the 2^nd ^iteration it detects other multi-TM O-acyltransferases, like O-acyltransferase 3 of *Clostridium cellulolyticum *(gi: 220930016). A reciprocal search with the *Clostridium *protein retrieves the Cas1p transmembrane region in the 2nd iteration (e-value = .005), along with numerous other O-acyltransferases of the classical acyltransferase superfamily that is found in both bacteria and eukaryotes. This superfamily includes proteins such as: 1) *Staphylococcus *OatA that carries out the O-6 acetylation of N-acetylmuramic acid in peptidoglycan to make it resistant to lysozyme [5]; 2) The MdoC and OpgC proteins that catalyzes the O-succinylation of periplasmic glycans of proteobacteria such as *E.coli and Rhodobacter *[6,7]; 3) NolL that acetylates fucosyl penta-N-acetylglucosamine required in *Rhizobium*-host interactions [8]; 4) GumG and GumF that catalyze two acetylations of the mannosyl moiety of the xanthan gum produced by *Xanthomonas *[9]. Most of these enzymes catalyze acylations by short chain fatty acyl groups and in this respect prominently differ from MBOAT superfamily of membrane-associated acyltransferases which catalyze transfer of longer, hydrophobic fatty acyl chains in the formation of waxes and lipid-soluble adducts to proteins [10,11]. A multiple alignment of the Cas1 multi-TM domain with the classical membrane acyltransferase superfamily shows that the conserved domain shared by all these enzymes consists of 10 TM helices (See additional file 1 for alignment). Several of the helices contain conserved residues: 1) a conserved histidine in the first helix; 2) a [ST]Ga signature (where a is an aromatic residue) in the second helix that might bind the nucleotide moiety of the acyl coA; 3) a conserved arginine in the 3^rd ^helix; 4) a conserved basic residue (K or R) in 5^th ^helix; 5) a conserved glycine in the 6^th ^helix; 6) a second conserved histidine in the and 9^th ^helix. Together, these are predicted to constitute a previously obscure intra-membrane active site that potentially acylates the sugar moieties in course of their delivery to the cell-surface. Thus, it is likely that the multi-TM domain of Cas1p and its homologs indeed catalyzes the acetylation of the mannosyl moieties of GXM through a mechanism comparable to the classical membrane-linked acyltransferases such as OatA, GumG or GumF.

An investigation of the phyletic patterns and affinities of these enzymes suggests that in addition to the Cas1p family there are 3 other distinct 10TM acyltransferase groups in eukaryotes, which have all emerged through independent lateral transfers from bacteria where this superfamily shows an enormous radiation. Some of these are restricted to lineages such as nematodes or fungi while others like the Cas1p and Beltless families are more widespread in eukaryotes (additional file 1).

### The N-terminal domain of Cas1p defines a novel acylesterase domain found in diverse eukaryotic proteins

**Figure 1:**
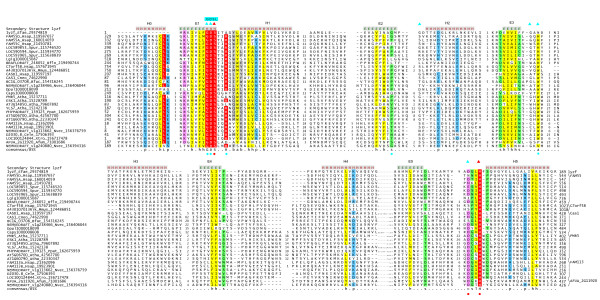
**Multiple alignment of representatives of the PC-Esterase family**. Multiple sequence alignment of the PC-Esterase domain was constructed using Kalign after parsing high-scoring pairs from PSI-BLAST search results. The alignment was refined based on the pairwise alignments produced by the profile-profile searches with the HHpred program using the PC-esterase profile. The secondary structure from the crystal structures is shown above the alignment with E representing a strand and H a helix. The 85% consensus shown below the alignment was derived using the following amino acid classes: hydrophobic (h: ALICVMYFW, yellow shading); small (s: ACDGNPSTV, green); polar (p: CDEHKNQRST, blue) and its charged subset (c: DEHKR, pink), and big (b: FILMQRWYEK; grey shading). The limits of the domains are indicated by the residue positions, on each end of the sequence. The numbers within the alignment are non-conserved inserts that have not been shown. The sequences are denoted by their gene name followed by the species abbreviation and GenBank Identifier (gi). The species abbreviations are Afum: *Aspergillus fumigatus*; Atha: *Arabidopsis thaliana*; Bflo: *Branchiostoma floridae*; Bfuc: *Botryotinia fuckeliana*; Caps: *Capitella spI*; Cele: *Caenorhabditis elegans*; Cneo: *Cryptococcus neoformans*; Dpul: *Daphnia pulex*; Efae: *Enterococcus faecalis*; Hsap: *Homo sapiens*; Lgig: *Lottia gigantea*; Mmus: *Mus musculus*; Nvec: *Nematostella vectensis*; Ppat: *Physcomitrella patens*; Spur: *Strongylocentrotus purpuratus*; Xtro: *Xenopus tropicalis*. The bacterial (1yzf) prototype of the GDSL/SGNH family is the first sequence shown. The conserved residues of this family are marked by triangles above the alignment, with the red triangles denoting active residues. The conserved residues of the PC-Esterase domains are shown using circles below the alignment, with the red ones denoting active residues. The PC-Esterase domains also have an extra N terminal Helix-0 that is not seen in the canonical SGNH domains. The PC-Esterase domain in the *Lottia gigantea *protein has a NxxxH instead of a DxxH, where the polar N has shifted one amino acid to the left. The metazoan C7orf58 have a S in place of the D in the catalytic triad, but the hydroxyl group of this residue can function equivalently. The subfamily names are shown to the right.

To investigate the globular N-terminal region in Cas1p we initiated PSI-BLAST searches of the non-redundant database (expect (e)-value for inclusion in threshold = .01) with this region from the human ortholog of Cas1p (gi: 119597197; region 207-428). In addition to recovering Cas1p orthologs, the search also detected numerous other eukaryotic proteins with significant expect (e)-values in course of successive iterations. For example, the search recovered the *Homo sapiens *C7orf58 protein (gi: 119603958; e = 8xe-06), the *Arabidopsis thaliana *pathogen resistance protein PMR5 (gi: 15237711; e = 4xe-03) and a group of plant freezing resistance/cold-acclimatization proteins typified by *Arabidopsis thaliana *ESKIMO1, the *Caenorhabditis elegans *protein D2030.8 (gi: 17506393; e = 6xe-04) and the animal FAM55D proteins (e.g. gi: 117647230; e = 5xe-03) within 5 iterations. Reciprocal PSI-BLAST searches seeded with the newly recovered proteins, in addition to recovering all their orthologs, also recovered several proteins of the former group with significant e-values. A multiple sequence alignment of the predicted domain was prepared with all the members collected in the above searches using the KALIGN program. A search with the PSI-BLAST program of the non-redundant database using a PSSM derived from this alignment recovered the so called FAM113 proteins from animals. Inclusion of the FAM113 proteins in the profiles recovers members of a previously characterized large esterase superfamily known as the SGNH/GDSL superfamily (e~10^-3^) [12]. To further test the validity of this relationship we performed a HMM-HMM comparison with HHpred program, using the HMM of the above characterized region homologous to the N-terminal globular part of Cas1p in a search against a panel of HMMs derived using the globular domains in the PDB database as seeds. The top hits in this search were the GDSL/SGNH Acyl transferases (e.g. pdb: 1yzf) with highly significant p-values (p = 1.4 × 10^-9^). A combined secondary structure prediction with the Jpred program by using residue frequencies in columns, a HMM and a PSSM derived from the alignment of the segments homologous to the N-terminal region of Cas1p showed a conserved core of 5 strand-helix units (Fig. 1). This secondary structure is entirely congruent with the secondary structure progression of the GDSL/SGNH esterase fold and strongly supports a common evolutionary origin for the domain prototyped by the Cas1p N-terminal region and the former esterase superfamily.

The GDSL/SGNH superfamily contains numerous well-known esterases from bacteria and eukaryotes such as acyl-coA thioesterase, aryl esterase, platelet activating factor acetylesterase and acetylxylan esterase [12]. The name GDSL is derived from the presence of a GDSL motif in the loop between the first conserved strand and helix, whereas the name SGNH is due to the presence of the conserved residues S (the S in the GDSL motif), G (in the loop after strand S2), N (found in a GxND motif after strand S3) and H (found in DxxH N-terminal to the last helix), which cluster around the active site. The domain family prototyped by the Cas1p N-terminal region has a mosaic of features that both resemble and differ from the canonical versions of the GDSL/SGNH superfamily (Fig. 1). Like members of the GDSL/SGNH superfamily, these domains share a catalytic triad comprised of a serine in an N-terminal GDS motif and an aspartate and histidine between S5 and H5, indicating these proteins are catalytically active esterases. However, unlike the canonical members of the superfamily they lack the conserved G after strand S2 and GxND motif after strand S3. This family however tends to have a nearly absolutely conserved W/H in the position equivalent to the N of the canonical GDSL/SGNH domains (Fig. 1). The nitrogen atom from this W/H residue could potentially position itself for forming a hydrogen bond in the active site, similar to the beta-carbonyl nitrogen of the N in canonical GDSL/SGNH enzymes. We accordingly termed this novel family the **PC-esterase **family (after the Pmr5 and Cas1p proteins and its predicted esterase function) to highlight its differences from the canonical versions GDSL/SGNH superfamily and the fact that most of its members are not recognized as such by the profiles/HMMs of widely used protein alignment databases (e.g. PFAM/Interpro).

**Figure 2:**
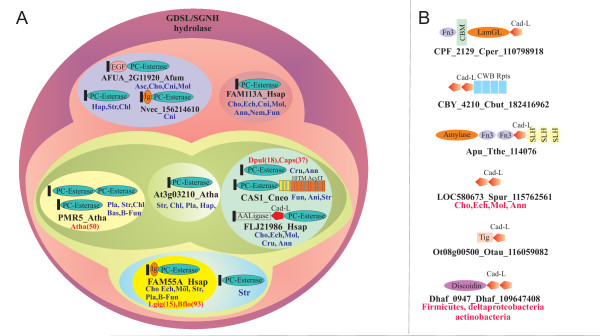
**A) A classification scheme for the PC-Esterase proteins**. Each family is represented by an ellipse while the higher level relationships like the groups and the core cluster are represented as ellipses surrounding them. The outer circle represents the classical GDSL/SGNH domains. The PSI-BLAST e-values provide a numerical measure of these relationships - each subfamily recovers other members of the same family at e-values in the range of 10^-64^-10^-7 ^within a single iteration; the member of any group are recovered by members of other groups in multiple iterations. The domain architecture representative of each family is shown along with the gene name and organism. The phyletic pattern of the family is given and any lineage specific expansion is shown in red. The class abbreviations are Cho - Chordates, Cru - crustacea, Ann -annelid, Fun - Fungi, Asc - Ascomycota, Basi - Basidomycota, B-Fun - Basal Fungi, Ani -Animals, Ech - echinodermata, Mol - molluscs, Cni - cnidaria, Nem - Nematoda, Pla - Plants, Chl - Chlorophytes, Str - Stramenopiles, Hap - Haptophyceae, and the species abbreviations are as above. B) Domain architectures of the Cadherin-like domainsThe domain architectures of the Cadherin-like (Cad-L) domains are shown. The gene name, Organism name and gi are given below. The phyletic pattern is also given, if the architecture is widespread. Domain abbreviations: Fn3 - Fibronectin 3; CBM - Carbohydrate binding domain; LamGL - LamG like jelly roll domain; CWB - Cell Wall Binding repeats.

Members of the PC-Esterase family are widely distributed across eukaryotes and are observed in the animal, fungal, plant, stramenopile and heterolobosean lineages (*Naegleria gruberi*). At least five distinct subfamilies can be recognized within the PC-esterase family (Figure 2 and Additional File 1) with maximum diversity (i.e. simultaneous presence of most subfamilies in a given lineage) observed in the animal lineage. The evolution of the PC-esterase family is characterized by multiple independent lineage-specific expansions of various subfamilies in both plants and animals. For example the subfamily that contains Cas1p has been independently expanded in annelids such as *Capitella *and crustaceans such as *Daphnia*. The subfamily prototyped by the Fam55 proteins is likewise massively expanded in the amphioxus *Branchiostoma floridae *and also in the snail *Lottia gigantea*. On the other hand the PMR5 subfamily is vastly expanded in several plant lineages (called domain of unknown function DUF231 in PFAM), including some of the early-branching forms such as chlorophyte algae. Given the evolutionary relationship to the larger GDSL/SGNH superfamily it is likely the PC-esterases branched off from them relatively early in eukaryotic evolution (Fig. 2).

### Domain Architectures of PC-esterases and identification of previously unrecognized associated domains

All members of the PC-esterase family have N-terminal signal peptides indicating that they are secreted proteins which function at the cell-surface. Four of the five distinct subfamilies contain at least one pair of cysteines located at the N- and C- termini of the protein suggesting that the termini of the PC-esterase domain are usually braced by disulfide bonds. The prototypical domain architecture of the PC-esterase family is the association with the classical 10TM acyltransferase domain as observed in Cas1p. Interestingly, a comparable combination of an esterase of the GDSL/SGNH superfamily and the 10TM acyltransferases is also observed in bacterial multidomain enzymes such as OatA suggesting that the functional association between these domains is an ancient one. Analysis of the domain architectures of the PC-esterase proteins showed that certain subfamilies are found fused to other known domains typical of cell-surface adhesion molecules such EGF and filamin-like immunoglobulin domains (Fig. 2).

In the animal-specific subfamily typified by the human C7orf58 we detected two additional domains fused to the N-terminus of the of PC-esterase domain (Fig. 2.). Sequence profile searches with the first of these N-terminal domains recovered the ATP-grasp domains with significant e-values within 3 iterations. The ATP-grasp domains of the C7orf58 subfamily were most closely related to the eukaryotic amino-acid ligases of the tubulin-tyrosine ligase family (TTL family; see additional file 1 for alignment) [13]. Examination of the alignment of this domain indicated that at least the orthologs from amniotes contain an intact predicted active site with conserved the two conserved lysines in the N-terminal module and the conserved acidic/polar residues associated with the last two strands of the C-terminal module of the ATP-grasp domain (Additional file 1). However, the versions from teleosts and molluscs apparently contain disruptions of the active site and are likely to be inactive. This implied that at least the mammalian versions are likely to catalyze amino acid ligation similar to the members of the TTL family that ligate amino acids such as glycine, tyrosine or tubulin to gamma carboxylates of glutamates or the terminal COOH groups of proteins.

Sequence profile searches with the second N-terminal globular domain the C7orf58 subfamily (gi: 31542727; region 550-690 ) recovered several bacterial proteins (e.g. CBY_4156 from *Clostridium butyricum*, CPF_2129 from *Clostridium perfringens*) and a few metazoan proteins (e.g. LOC580673 from *Strongylocentrotus purpuratus*). A profile-profile comparison with the HHpred program significantly recovered the cadherin domain and a comparison of the predicted structure of this domain with the crystal structure of the cadherin showed a congruent seven stranded secondary structure. This suggests that the group of domains prototyped by the second globular N-terminal domain of C7orf58 defines a new family of beta-sandwich domains with a cadherin-like fold. The domain is widespread in bacteria and seen in the firmicutes, actinobacteria, certain proteobacteria, bacteroides and chlamydiae with a notable expansion in *Clostridium*. In contrast, it is limited in eukaryotes thus far to metazoa and chlorophyte algae, suggesting that it was derived through lateral transfer from bacteria. In prokaryotes, this cadherin-like domain is widely fused to other domains such as FNIII (Fibronectin Type III), TIG, SLH (S-layer homology), discoidin, cell-wall-binding repeat domain and alpha-amylase-like glycohydrolases (Fig. 2). These associations are suggestive of a carbohydrate binding function for this cadherin-like domain.

### Functions of 10TM acyltransferase, PC-Esterase and C7orf58-like ATP grasp proteins in eukaryotes

Combination of the evidence from previously published studies, sequence analysis and contextual information from domain architectures indicates that the enzymes discussed here represent a constellation of novel activities that modify cell-surface biopolymers such a glucans or glycoproteins. Domain architectural analysis of this family indicated several domain fusions, the majority of which are indicative of a function on the cell-surface. Interestingly, not only in Cas1p but also in the case of several bacterial proteins two opposing activities, namely acyltransferase and acylesterase activity are observed in the same protein (Fig. 2). This apparently paradoxical architecture might have profound implications for the compositional regulation of cell-surface glycans. It is known that acylation of glycans reduces the number of available free hydroxyl groups and greatly affects their properties such as hygroscopicity, physical strength of fibers, association with cations and resistance to degradation by enzymes such as lysozyme [5,14]. Hence, we predict that combination of the opposing esterase and acyltransferase activity in the same protein results in 1) optimization of the level of acylation in the extracellular polymer to achieve desired physical strength - optimum strength coincides with an optimal acylation level [14]; 2) differential solubility - increased hydrophobicity might help transport across the hydrophobic membrane, possibly through the pore formed by the 10TM domain of the acyltransferase; 3) turn over and processing of the glycan -removal of acyl groups would make the glycans more soluble and more amenable to degradation and recycling. Thus, the combination of an acyltransferase and acylesterase domain could act as a switch that regulates the recycling of a glycan.

The lineage-specific expansion of the PC-esterases in various eukaryotic lineages is reminiscent of the comparable expansions of other surface proteins typically associated with host-pathogen interactions and stress responses [15]. Such expansions (e.g. those of TLR receptors and immunoglobulin domain proteins) typically provide a means for the organism to recognize a diversity of pathogen cell-surfaces and respond appropriately. In particular there is evidence that acylation of surface glycans in bacteria, such as *Staphylococcus*, makes them resistant to host defenses such as lysozyme [5]. Hence, it conceivable that some of these lineage-specific expansions, especially in various animal lineages, play an important role in the removal of acyl group from pathogen glycans to make them more amenable for degradation by glycohydrolases. Mutations in the plant protein PMR5, have been shown to cause an increase in the content of galacturonic acid, a major component of pectin in the cell-wall [16]. Further, a paralog of PMR5, ESKIMO1 is implicated in response to freezing stress and water status [17,18]. Mutations in this gene resulted in improved water utilization and reduced transpiration rate [19]. Based on our identification of a PC-esterase domain in PMR5 and ESKIMO1 we suggest that in both the cases the primary biochemical role of the esterase is modification of the cell-wall glycan properties by removal of acyl groups. In the case of the PMR5 mutant, loss of the esterase activity might result in suboptimal levels of glycan acylation, which subsequently results in decreased physical strength and susceptibility to pathogens. The reduction of transpiration and improved water use in the ESKIMO1 mutants might be the consequence of the more hydrophobic glycans, formed as a result of loss of esterase activity, helping to retain water.

The previously unidentified ATP-grasp domain fused to the PC-esterase in the metazoan C7orf58 subfamily is remarkable in being the first secreted TTL-like enzyme in eukaryotes. The PC-esterase domain of these proteins could function just like its homologs in other contexts in hydrolyzing glycan esterases. However, the presence of an active amino acid ligase domain in certain members of this family points to the possibility that there might modification of surface proteins by ligation of amino acids. Alternatively, it could be involved in the formation of a composite peptide-glycan copolymer as seen in bacteria [20]. Its rapid evolution and multiple losses/inactivations in metazoans are consistent with a potential role in modifying cell-surface components in the context of host-pathogen interactions.

## Conclusions

We identify novel enzymes predicted to modify cell-surface biopolymers such as glycans and glycoproteins. We present evidence that the action of some of these enzymes might be critical in regulating physico-chemical properties of endogenous as well as exogenous glycans as part of defensive mechanisms against pathogens and stresses. We also present evidence for a secreted amino acid ligase in metazoans that is predicted to catalyze a novel modification of cell-surface polymers by means of peptides or amino acids. The results presented here might help in the further exploration of neglected aspects of eukaryotic cell-surface biochemistry.

## Competing interests

The authors declare that they have no competing interests.

## Authors' contributions

VA and LA were involved in the discovery process and writing the paper. All authors read and approved the final manuscript.

## Reviewer's Comments

### Reviewer 1

Frank Eisenhaber, Director, Bioinformatics Institute (BII) Agency for Science, Technology and Research, Singapore

1. *Although the theory of sequence homology is general, our sequence-statistical criteria have only been proven for globular domains. Essentially, the conservation of an apparently random hydrophobic pattern that is necessary for composing the hydrophobic core of the globular structure serves as a strong criterion for fold conservation and this is the reason for a high score. For various non-globular segments that have amino acid compositional bias or simple repetitive patterns, sequence similarity does not necessarily mean homology. It is not rare that PSI-BLAST collects groups of proteins that just have transmembrane regions in common, especially if the inclusion cutoff is quite generous as in this work. This is relevant for the 10TM sugar acyltransferase family collection. Are there other arguments for homology than just the conservation of a few functional residues that are hypothesized to form an intramembrane active site? Are there observations of finding irrelevant proteins in the hit lists?*

Response: It is true that compositional bias can cause recovery of similarly biased non-homologous proteins in profile searches, including in the case of TM proteins. Searches with TM proteins should be examined on a case-by-case basis to rule out spurious relationships. In this case there are number of factors that favor the reported relationship to TM acyltransferases: 1) the similarity consistently extends across 10 TM domains rather than just a few TM segments as is usually seen in the case of spurious hits between membrane proteins. 2) The intra-membrane conservation patterns include a unique set of *polar *rather than just hydrophobic residues (the latter might easily arise by chance alone due to bias). 3) For at least 4-5 iterations these searches remained "clean" in recovering only known acyltransferases and their homologs. These observations, together the functional congruence between the TM domains of the Cas1p family and the classical acyltransferases, favor homology as the explanation for the detected similarity.

2. *You write that most PC-esterases have at least a pair of conserved cysteines; "most" being a not very scientific determination. Can this conclusion be supported with quantification?*

Response: Four of the five subfamilies of PC-esterase domains contain these pairs of the cysteines.

### Reviewer 2

Gaspar Jekely, European Molecular Biology Laboratory, Heidelberg, Germany

I approve publication of the manuscript. I don't have any major comments, it is a nice work.

## Supplementary Material

Additional file 1 Material and methods, comprehensive alignments of the 10TM Acytransferase and Cadherin-like domains and a complete list of families are provided. Click here for file
