# Supplementary material for: Novel eukaryotic enzymes modifying cell-surface biopolymers
Source: Biol Direct. 2010 Jan 7;5:1. doi: 10.1186/1745-6150-5-1 (PMC2824669; doi:10.1186/1745-6150-5-1)
Supplement: Additional file 1 — Material and methods, comprehensive alignments of the 10TM Acytransferase and Cadherin-like domains and a complete list of families are provided. [file 1745-6150-5-1-S1.HTML]

Novel eukaryotic enzymes modifying cell-surface biopolymers


# Novel eukaryotic enzymes modifying cell-surface biopolymers.

Vivek Anantharaman and  L.
Aravind\*  
  
\* *Address
for
correspondence: L. Aravind (aravind@mail.nih.gov)*  
  
*National
Center for Biotechnology
Information, National Library of Medicine, National Institutes of
Health, Bethesda, MD 20894, USA*


---

  

Abstract  

Eukaryotic extracellular
matrices such
proteoglycans,
sclerotinized structures, mucus, external tests, capsules, cell walls
and waxes
are abundant in highly modified proteins, glycans and other composite
biopolymers.  Using comparative genomics
and sequence profile analysis we identify several novel enzymes that
could be
potentially involved in the modification of cell-surface glycans or
glycoproteins. In particular we objectively define the acyltransferase
domain
prototyped by the fungal PC-Esterase proteins, identify its active site
residues and
unify them the superfamily of classical 10TM acyltransferases (e.g.
oatA). We
also identify a novel family of esterases (prototyContentsped by the
previously
uncharacterized N-terminal domain of PC-Esterase) that have a similar
fold as
the
SGNH/GDSL esterases but differ from them in their conservation pattern.
We
posit that that combined action of the acyltransferase and esterase
domain
plays an important role in controlling the acylation levels of glycans
and thereby
regulates their physico-chemical properties such as hygroscopicity,
resistance
to enzymatic hydrolysis and strength. We present evidence that the
action of
these novel enzymes on glycans might play an important role in
host-pathogen
interaction of plants, fungi and metazoans. Based on our findings we
present
evidence that in plants (e.g. PMR5) the regulation of acylation by the
acylesterases might also play an important role in regulation of
transpiration
and stress resistance.  We also identify
a subfamily of these esterases in metazoans (e.g. C7orf58), which are
fused to
an amino acid ligase domain that is predicted to catalyze a hitherto
unrecognized modification of cell surface polymers by amino acid or
peptides.

  


---

Contents  
  

- Materials And Methods
- Multiple
  Alignment of 10TM Acyltransferase
- Multiple
  Alignment of Cadherin-Like domain
- Family
  clusters of PC-Esterase domain containing proteins with domain
  architecture
- Family
  clusters of 10TM Acyltransferase with domain architecture
- Family
  clusters of Cadherin-Like domain containing proteins with domain
  architecture

  


---

Materials
And Methods  
  

The
non-redundant (NR) database of protein sequences (National
Center for Biotechnology
Information,
NIH, Bethesda)
was searched using the BLASTP program [1].
Iterative
database searches were conducted using the PSI-BLAST program with
either a
single sequence or an alignment used as the query, with the PSSM
inclusion
expectation (E) value threshold of 0.01 (unless specified otherwise);
the
searches were iterated until convergence [1,
2].
For all searches with
compositionally biased proteins, the statistical correction for this
bias was
employed. Multiple alignments were constructed using the KALIGN
programs [3,
4],
followed by manual correction based
on the PSI-BLAST results. Globular domains were predicted using the SEG
program
with the following parameters: window size 40, trigger complexity=3.4;
extension complexity=3.75 [5].
All large-scale
sequence analysis procedures were carried out using the TASS package
developed
by our group (unpublished; Vivek Anantharaman, Santhanam Balaji, and L.
Aravind). Protein secondary structure was predicted using a
multiple
alignment as the input for the JPRED program [6].
Similarity-based
clustering of proteins was carried out using the BLASTCLUST program
(For
documentation see ftp://ftp.ncbi.nih.gov/blast/documents/README.bcl).

 References

1.           
Altschul
SF, Madden TL, Schaffer AA, Zhang J, Zhang Z, Miller W, Lipman DJ:
Gapped BLAST and PSI-BLAST: a new
generation of protein database search programs. *Nucleic
Acids Res* 1997, 25(17):3389-3402.

2.           
Aravind L, Koonin EV: Gleaning non-trivial structural,
functional
and evolutionary information about proteins by iterative database
searches.
*J Mol Biol* 1999, 287(5):1023-1040.

3.           
Lassmann T, Sonnhammer EL: Kalign--an accurate and fast multiple
sequence alignment algorithm. *BMC
Bioinformatics* 2005, 6:298.

4.           
Lassmann T, Sonnhammer EL: Kalign, Kalignvu and Mumsa: web
servers for
multiple sequence alignment. *Nucleic
Acids Res* 2006, 34(Web Server
issue):W596-599.

5.           
Wootton JC: Non-globular domains in protein sequences: automated
segmentation using
complexity measures. *Comput Chem* 1994,
18(3):269-285.

6.           
Cuff JA, Barton GJ: Application of multiple sequence alignment
profiles to improve protein secondary structure prediction. *Proteins*2000, 40(3):502-511.

 

   
  
  
  
  
Top  


---

Multiple
Alignment of 10TM Acyltransferase   

|  |
| --- |
| ..........................................A1...........................................................A2........................................A3......................................................................TM1........................................TM2.........................TM3...........................................................TM4....................................................TM5................................................TM6..............................................TM7.......................................TM8....................................TM9..........................................TM10                                                        out         >>>>>>>>>>>>>>>>>>>>>>>>-----in------------------------------------------->>>>>>>>>>>>>>>>>>>>>>-Out------------>>>>>>>>>>>>>>>>>> in---------------------------------------------->>>>>>>>>>>>>>>>>>>>>>>>-----------------out->>>>>>>>>>>>>>>>>>-----------in>>>>>>>>>>>>>>>>>>>>>>>>>--------------------------out------>>>>>>>>>>>>>>>>>>>>--in--------------------------->>>>>>>>>>>>>>>>>>>>>>>>>----out------------------->>>>>>>>>>>>>>>>>>>>>>>--------in--------->>>>>>>>>>>>>>>>>>>>>>>>>--out---------------------->>>>>>>>>>>>>>>>>>>>>>>---in--------->>>>>>>>>>>>>>>>>>>>>>>------------------------->>>>>>>>>>>>>>>>>>>>>------------  CNN01530\_Cneo\_58262436               LSWRCNDVMRH---------E-GATGTCCKRYDWVTPIQGLILAVLILWAPL----G----TF-ITPRLPPNSPILDY-------------------------LPATSIAPALSTFGLAMGYLFLADRTHVFQKEQKDYDAVIFGMITLAAFVAGLLTIKNSGK------------------------------DLGFLNRDITDEWKGWMQIAILIYHFFGASKI-SGIYN-----------------PIRVLVASYLFMTGYGHFFFYYKK---------ADFG--FQRVVMVLVRLNLLSVVLPYT-------------------------MNTDYA----FYYFAPLVSWWYLIIYATMAIGSKYNDRP---------------------AFLLTKLFTCAGLVTLFMH-FP-----WLMEDVFKV--LNTVFNI-QWSAKEWSFRVTLDLFIVWVGMLCAYGFVKFN-----EHQISDRPWFPVMRTAT-----LVGSVLGMIWYFWFE-LHLASKF--------------VYNEYHAVVCIVPIMSFVFLRNASPVLRSSTSKIFCFIGQCSLETFILQFHGWLASDTKAILLA--------V-PSTQWR--------PVNLVISTICFIWLSYRVSGATGEITEWLVGKKK  FG00106.1\_Gzea\_46103822              LNLRCNAKLDRIK-------PYPYKRTCCTDYGIKPLTQLGIVALGIIYLVACLVGE----ILDLYHDRSE--------------------------------PHWGLFNMKVGSFILALLMCYYADRTQMMAKGEKLWLPIDFAVLCAPCIAILLLTIRRSRSPISMDMSLLTKET-----------------NESFLSRHQTEEWKGWMQAVILIYHWTGAIKGSKSIYI-----------------LIRLCVAAYLFQTGYGHTLYFVRK---------NDFS--FRRVATVLLRLNVLSCSLAYV-------------------------MDTDYM----FYYFSPLVSFWFLVVYATMAVGGKRFNSDP--------------------QIVLSKICISGLLISAIFM-CT-----PFTQFVFGL--LKTVFNI-QWSYETWQYRVTLDMFIVYAGMLTAVVHNEMK-QT--SVHLGLRIILAF------------AGLFVTMYYFKS--TLHLRHS--------------VYKTWHPLVSFIPIIAFITLRNISATIRNYHSKAMAWLGRCSLETYILQYHILMAADTEGVLIVDGLFGDGTV-LGDRWR----------TLIIIVPLFLWVSNAAAISTGYVVELI-----  FG03124.1\_Gzea\_46114564              LNLRCNAKLDMQN-------SYPYDRTCCTNYGQKSFVQIILVALGMLYVGGAAI-T----EIIALRSGEA--------------------------------PKWSFFNLDVATFVTGLLACYWADRTQSFAKGSKQYSMFDFNLMAALCFIVGFALMTKSKPPPPRPGAQAAAAAAPAA-PATLD-------DAKPLSRDQTDEWKGWMQALILVYHWTGASRD-LNIYV-----------------GIRLLVAAYLFQTGFGHGVFFSSK---------KDFS--FKRVAAVLLRLNLLSVALPFF-------------------------MNTDYM----FYYFAPLVSFWFLIIYSLFAICPKYNDNT---------------------WALMGKIAISAAICPGAML-WT-----PVLQWVFDA--LNIVFRI-EWDLHEWQFRLGLDGLIVYVGIIMGVASVRTKLYN--KILTQSYGLAGI------------AGILSIPLYWWVAVSKAEKKQ--------------DYTALHPIFSFIPIMGFIAARNMFPAARTWYSRSFAWIGRCSLETFTLQFHILLAADTKGLLLLDIFKGDGSL-LCDRWR----------SLIIIAPVFLWISSRVADATGGMVKLLTIDWA  FG10575.1\_Gzea\_46138121              LNLRCNAKLDRMR-------RSPHGGTCCTDYGVTQYRII-IAVVGIVYLSACVV-C----EILDL-TSAK--------------------------------EAWSLLNMRIGSFVLVLLMCYFSDRTQMMAKSSKLWEVDGFAILCAACLLPLLVTIRRTRPKSHQHLPSTEDETSEKLLPENPELEENYQQDEPFLSRTQTEEWKGWMQCFVLIYQWTGADQGPISLYI-----------------LFRLCIAAYMFQTGYGHAVYFITT---------SDFS--LKRVVTTLLRLNAFSCALAYS-------------------------MNMDYM----FYYSAPLASFWFLVIYATMAIGKQCNSDT---------------------QMVVAKVCISGVLVFAMFV--T-----PLPRWIFNL--FEIIFNI-QWSADQWIRYATLDMFIVYIGMVTAIV-SQMG-GT--QIILSLRLMLGL------------AGVFATCYYFIK--GSTLSQS--------------SYDSLHPYLSSIPILGYVALRNISVYNRNYHSKAMAWLGRHSLESSILQSHILLAADREGVLSIDGLFGDSTI-LGDRWR----------SLLIVVPIFLWTCYIARSATAYIIELL-----  FG03602.1\_Gzea\_46115520              FNMRCNAKLDRM------------------DY-----------------------------------------------------------------------HHWNLFNMRVGCFVLALLMCYYADRTQMMAKGSKPWQIGDFIALCLPCIAICLSTIRRSDPPRYLSLTQPST-------------------DQPFLSLDQIDEWKGWIQAFILICHWTGAQEG--SIQV-----------------LVSLCVGAYIFQTGYVHTLDFMNK---------KGFS--FNHAASTLFRLNILSCLLAYF-------------------------MDTDYM----TYHLSPLLSFWFLVVYATVAVDRQQNNEL---------------------KFLLVKICTSCMIISFIFL-DT-----PSTSWTFNI--LQGIFKI-QWRVEEWQRSVTLDLFIAYVGMLAAVIGREMK-KAEVSVRLGLRVCLAF------------GGLFSILHYLSF--TSHVRES--------------SYMKWHPYVSAIPILGFVMLRNIPWSTRNYHSRAMVWLGRCSLGTYTLQFHILLAADSKGILIVDGLFGDGTL-LGDRWR----------TLVIIVPIFLWISHSVAASTAYMVKLM-----  FG07886.1\_Gzea\_46127017              LNLRCNAKMDRRS-------GYPYTRTCCTDYGGRSWVQIVILAIIAIYSIACV-------FFEGFTFVSS--------------------------------LTVKGVDMKVGIFAVALIYCYAADRTHLFSKGMKEFVSNEFYLLSGICAIFGGLTIRKVQFRAPRPPPAVVATEPESEPTPAPVTPVVQ--DAGILARDQTEEWKGWMQAAILVYHWTGASTS-LPIYI-----------------FIRLLVAAYLFQTGYGHTIYFLSK---------KDFS--FRRIASVLLRLNILSCALPYV-------------------------MGTDYM----FYYFAPLVSFWFLVVYVTMAVCSSFND-S---------------------FKVVGSKILASFIFFTLVLNVT-----PLMKWLFAI--LELVFRI-KWDLNEWEFRVTLDGAIVFVGMLAGVVHQRVE-RD--AFWYTNYKFAVI------------PSLIAVFGFGYFC-SSMETKA--------------IYVLLHPVISIFPVLGFIGLRNAITPFRNRYSTAAAWLGKCSLETFILQFHVFLAGDTRGVLLLDIFKGDGSL-INDRWR----------ALAVIVPVFLWISHLVAEASGQIVKLIMGESK  YALI0C00187g\_Ylip\_50547609           RNVRCNQELLDHKYDITNGPVYPFSDTCCIHYKQPRTMGQVLLLGVICAILPIIYLGQ---R-YTRSDYYPRFVRYVSWPVPS--------------------IQDETLLTACVVIAASLAYSFFCDRTQFFGKSSKQFEASEFWVLILLFLVATGYSFEPQN-------------------------------DNSFLNRHQTEEWKGWMQIIILIYHITGASKI-LPIYK-----------------FVRVLVAAYLFMTGFGHATFFIKR---------GDFS--LKRATSVLFRMNFLSILLAYV-------------------------MDTDYL----FYYFAPLVSFWFCVVWITFRVLPSWNSIDT------------------SVGPVLAKISASAVILNILVR-FQ-----LPFQIVFAV--LKYLFNI-QWNLREWRFRLILDIYIVYIGMFAAVATLRYK-----QGNFPLKNLVTN----R-----LVLGVVALITFIVY--IAVAASFGIKQ----------NYNQAHPYISWMPIVAFVILRNITVRFRSHYSTWMSFIGTCSLETFTLQFHFFLAADTKGRLYI--------L-NTPTSSGVLGMLQKYLNFGLVTVIFIIVSHAVAEQSNALTKLFVGNQD  Pbla1000006496\_Pbla\_Pbla1000006496   LNFRCNQELPK---------TFPMDTTCCYTYPSPQWFQTVWFLVPLVWVPF---------CFLVANYEKRQDQFFVKYL-----------------------VPSNNVLYALLTMGLGVGYMYLGDRTQLFGKIHKVFQPVVFSGLLLLLLGVGLVTLRKKESG-----------------------------DQGFLNRAQTDEWKGWMQIIILVYHFLGASSV-SGIYN-----------------PVRVLVAAYLFQTGYGHFFFFYKK---------GDYS--FGRVLSIMVRLNLLTFVLQYL-------------------------MNTNYL----SYYFSPLVSFWFIIIWVTMYVGNKWNKTP---------------------SFLLAKMFGMAMLTTLIIH-YP-----GLLEFAFKC--LEYTFNI-QWNPVEWRFRLALDAWIVYVGMLIAYLNIKIT-----EQKLPSKAYWSTAKNAS-----IVVSCVAMIWYFWFE-LSRPNKF--------------VYNTYHPYISWIPVLAFTILRNCTLGLRNRSSRFFEFIGKCSLETFIGQFHMWLAADTQGLLLI--------L-ANPMWAHGLGW---WVNLGLSTVLFVFVSYHLSQATGEITQWICSGAN  CC1G\_10518\_Ccin\_169843844            LNLRCNDVLPK---------KFPFNKTCCNSYPWPNYVHSFLLFAIIVSAPLI--------C--YLTASPGTRSVML--------------------------NATNPSNLPLLVLSGGLIAIYLSDRSWLWLKEHKQFDAINFTFLCLLAVGIGLASLKRADN------------------------------DLGFLNRDQTDEWKGWMQ---------RASKV-SGIYN-----------------PIRVLVASYLFMTGYGHTTFYLRK---------ADFG--FKRLAQVLIRLNLLTVLLAYV-------------------------MDTDYI----SYYFSPLVSMWYLVIYGTMAIGSQLNSRT---------------------PILLLKILVSATLMTSFMW-KS-----QPLEAVFSF--LELIFGI-RWSAREWSFRVNLDLWIVYVGMLTSIFVVKAR-----ENRLTDHRLWPLTTKIA-----IGGSILVLVWFFAFE-LCQDSKF--------------TYNRWHPYISLLPVLAFVILRNASVILRSASSRAFAFVGRCSLETFIIQYHFWLAGDTKGVLLV--------I-PGTRWR--------PANFVLTSIMFLYLSDRMAWATNQVTAWICSDGA  Ppla1000007369\_Ppla\_Ppla1000007369   LNLRCNDALPK---------TFPMDKTCCRRYPLPSPLHSIVLVAAILWGPV---------C-IVLTIGPGSRPPGQ--------------------------QLVGESELPAVVVSAASALIYTADRTGFWLKEQKQFSPWTFSFLSLLSLTVGLVTVRRGDK------------------------------DLGFLNREQTDEWKGWMQ----------TSKI-SGIYN-----------------PIRVLVASYLFMTGYGHTTFYVKK---------ADFG--FTRIAQVMIRLNLLTLLLAYT-------------------------MNTDYL----SYYFAPLVSWWFLVIYGTMLVGSRFNDRT---------------------VFLVCKIFLSMGVVTWFMS-EQ-----WLLETIFEF--LKRTCGI-HWSAREWAFRVNLDLWIVYIGMFTALAVMKIR-----EHRLTDHPQWPLIVKAS-----AGASGVVLLWFFAFE-LYQPDKF--------------AYNLWHPYVSFLPIAAFVVLRNANVILRSASSRMFAFIGTCSLETFIIQYHFWLAGDTKGILLV--------I-PGTRWR--------PVNLILTTVMFIYLSHHVAQATGEITNWVCGSSK  Ppla1000003910\_Ppla\_Ppla1000003910   LNLRCNDALPK---------TFPMDKTCCRRYPLPSPLHSIVLAAAIFWGPVGGCNIVI--REIVLTIGPGSRPPGQ--------------------------QLVGESEMPAVVVSAASALIYIADRTGFWLKEQKQFSPWTFSFLSLLSLTVGLVTVRRGDK------------------------------DLGFLNREQTDEWKGWMQ---------RASKI-SGIYN-----------------PIRILVASYLFMTGYGHTTFYVKK---------ADFG--FTRIAQVMIRLNLLTLLLAYT-------------------------MNTDYL----SYYFAPLVSWWFLVIYGTMLVGSRFNDRT---------------------VFLVCKIFLSMGVVTWFMS-EQ-----WLLDTIFEF--LKRTCGI-HWSAREWAFRVNLDLWIVYIGMFTALAVMKIR-----EHRLTDHPQWPLIVKAS-----AGASGIVLLWFFAFE-LYQPDKF--------------AYNLWHPYISFLPIAAFVVLRNANVILRSASSRMFAFIGTCSLETFIIQYHFWLAGDTKGILLV--------I-PGTRWR--------PVNLILTTVMFIYLSHHVAQATGEITNWVCGSSK  BRAFLDRAFT\_66940\_Bflo\_210128365      -------------------------------------------MVGAML---------------FSVTRGRTPSVSEDAETAQNQKKTKSTA-----------EIARTTLVALAKFGVIMAYFYLCDRTPLFMKENKHYTHLQFFVPFVWVLLVGLFFHSDTK-------------------------------Q------------------------------Y-LPIYM-----------------HIRILVAMYLFQTGYGHFFYFWMK---------GDYG--IVRLCQVNFRLNFLVVFLCMV-------------------------MDRPYQ----FYYFVPLVTFWFFVVYGTMAVLPRVTAKSSGD-------------NSSGFVLMMFKLLVLCGIIALLAS-MQ-----VLFESLFSWWPAVQLFQL-EGSIREWWFRWQLDRYAVSYGMFFAFTYLGLK-----KLQIIDDSFHGNLFTPCVTYLIVTLSVCITLGYTIFM-TTCSSKV--------------ECNRLHPYISFLPITSFILVRNVPGYLRSRYSTFFAWFGKISLELFIGQYHIWLAADTKGVLVV--------I-PGHHTL----------NMLVTSFIFVCIAHEISAVTGTLATTL-----  NEMVEDRAFT\_v1g238466\_Nvec\_156406044  LNFYCNRYMQV------------NDASCCLPTPRITQLQYNTFAVFVTCFVLFIIM-----FVCRRMWPPSEQDGTAGVEATTPGGHESYSLHWVYS------ESMYPVMRYMAKLGIIMLYFYLCDRTNLFFKEQKQYSNTAFFLSMLGFLILG----------------------------------------------------------------------------------------------------------------AYTWTP---------HD---------EVMTRMNLFTVVLCLV-------------------------MGRPYQ----FYYFVPLISFWFVVIYATMVFFPRVSASSVRE-------------DPKQYIFIWLKFFVLFGTIYILWS-SP-----ILFDWVFSQWAVKQLFIDENDSVREWRFRSWLDRYIVLYGMVFGFAYHTAK-----HFKIFDDTLRKGLFKSLHSKVTMVMSVVALAVYGIQA-FTCSNKP--------------SCNATHSVASCIPITAYILLRNVPGSMRSRFSRFYAWVGSISLELFIGQYHIWLAQDTRGVLVL--------I-PGYPAL----------NAILTSFVFVCVCHEVNK--------------  BRAFLDRAFT\_107904\_Bflo\_219498463     LNLFCNDRLRP------------IDGSCCQSYQPPSLLQVATGVFFGLCMVGAML---------FSVTRGRTPSVSEDAETAQNQKKTKSTA-----------EIARTTLVALAKFGVIMAYFYLCDRTPLFMKENKHYTHLQFFVPFVWVLLVGLFFHSDTK-------------------------------QTSILNRDQTDEWKGWMQLVILIYHYTGTSSY-LPIYM-----------------HIRILVAMYLFQTGYGHFFYFWMK---------GDYG--IVRLCQVNFRLNFLVVFLCMV-------------------------MDRPYQ----FYYFVPLVTFWFFVVYGTMAVLPRVTAKSSGD-------------NSSGFVLMMFKLLVLCGIIALLAS-MQ-----VLFESLFSWWPAVQLFQL-EGSIREWWFRWQLDRY----------------------------------------------------------------------------------------------------------------------------------------------------------------------------------------------------------  LOC100177911\_Cint\_198430770          LNLLCNQMTHA------------TDGTCCRKPDPLTIVQIIMAVLGFIFILL---------AVGIRIQRIGKPTPTNEDEVAKNKDSIH--------------NIIAELTYAAAKMSLILFYFYICDRTDVFMKSNKHYTNTRFFLPLLYIVFLGIFGIDSTK-------------------------------QPVFLNRDQTDEWKGWMQLVILIYHVTGASVN-VPIYM-----------------HVRLLVAMYLFMTGYGHFSYFWNK---------GDFG--VHRVFGVMFRLNFLTVMLCLT-------------------------MDRSYQ----FYYFVPMCSFWFLVLYLFMALWPRAYMVATNKETTLSDGETKEVTFTSPIFVMCAKLSLLLFTIVLVFL-SQ-----ELFESMFSWWPVIRLFELPPGMVREWWFRCHLDRFAMLHGAVFAFGYIILK-----RLSIVDDSRQGCLLSTKVSLIAVTASILCTLFYSVWA-LQCSDKQ--------------SCNEVHSFASLIPISAFILIRNIPGYLRAGYSSFFAWFGKISLELFIGQYHIWLAADTKGILVI--------TSPQWPML----------NLLVSTFVFVCVAHEISALTGTFAKYLVGPAK  Caps1000021394\_Caps\_Caps1000021394   WNLLCNEHLQF------------RDGQCCTRPEAVSNLQLFTAAFFFLWFHIYLPIHSILIAAPLWLLTRETKKPSLDLEKAAVDAKAAPN------------LKKDALVA-LAKLGLIMTYFFLADRNNYFMKTNKHYSHLHFFLAFIYFVFLGLFFSEKSK-------------------------------QTKVLHRDQTDEWKGWMQIVILIYHYTGASQV-LPIYM-----------------QVRVLVSTYLFTSGFGHFCFFWNK---------ADYS--IHRFCMVMFRMNFLVIVLCFV-------------------------MNRPYQ----FYYFVPLVSFWFLVIYSTMAMWPRVSAATQGAY------------SSGGVLYMLLKFFLLVAIITLFYA-SE-----VLFEQVFLTQPIQSLFVSADSSIHEWRFRWQLDRFSTIYGMVFGLFFILGQ-----KVKLWDDSGDAGLFSVPVNAAVGFVALIGFIGYSVFA-STCESKP--------------SCNHIHSYIAFIPIVCYILLRNLPGWLRVRYSTLFAWFGRISLELFIGQYHIWLAADTQGLLVL--------I-PSSPGL----------NTLLCSFIFVCIAHEIHCITGVLAPILVPSDL  Hrob1000020574\_Hrob\_Hrob1000020574   FNLICNNYMNF------------DDGSCCTSPENPTTVQIAMAAFFATSLAAAVLI-----ATCRRLNGRSNKNNKNNNKTNNNNNNQSKN------------MDCPTLISCIAKLGLILLYFFICDRTTFFMKENKYYTHVNFFLPFTYLMILGLFFTESTK-------------------------------QVQVLNRCQTDEWKGWMQIVILIYHLTGASKV-LSIYM-----------------HIRVMVSAYLFLSAYGHFVYFFNK---------NDFS--FHRLCTVLVRFNLLVVLLCLS-------------------------MNRPYQ----FYYFVPLVTFWFIVIFIVMALPPRSSSSM----------------SISSFFWIFLKIVGLAVVVTLLYS-SE-----VFFDQLFLSTSLKPLFVTSDESIHDWRFRWNLDRYSSIYGLLFALALVMAK-----RFKLIDDSVSDLVASRKVNMLSMALSICGLVGYAVFT-SLCNSKE--------------QCNNNNNK----KILSYICLRNMMGCARRKFSAFFAWFGKMSLELFIAQYHIWLAADTHGILVL--------I-PSYPVM----------NLIVTSFVFVCVAHEIKLLTSLLTPVLVPRSR  Dpul1000014677\_Dpul\_Dpul1000014677   MNMYCNERMDF------------NDGTCCRSPESATTLQIVTFTILLTLMEAEDDE-----MAKKDVVVANGHSTVVVAEGFDEKATTAHRKAAVEDVE----SSSRVVVTALGKLGLIMAYFYLCDRTTFFMKENKYYSHLNFWVPVGYVFALGLFFNEESR-------------------------------STKVLHRDQTDEWKGWMQLVILIYHMTGASQV-IPLYM-----------------QMRVLVSSYLFLTGFGHLSFFWNG---------GTAS--FPRLFQVLFRMNLMTVVICLC-------------------------MNRPYQ----SYYFVPLVSFWYLVVYIVLAVPPKVSAAICDA-------------NSFAYLYIIIKFCTLIGAITILYM-SE-----VFFETIFLIRPWKALFVTSSDEIHEWWFRWSLDRYSICYGMIFGFLYLNAQ-----KFGLIDDSTRQHLLSRTIRFLVVLAALIGLGGYTAFT-ITCHSKP--------------ECNEVHSYLAFLPIISFIVLRNVFGPLRVRYSSFFAWFGRISLELFIGQYHIWLAADTHGVLVL--------I-PNYPVL----------NVMVTTYILVCVAHEVHTITGQLVTLAVPSDW  AgaP\_AGAP001402\_Agam\_158302105       LNMYCNDYMNY------------NDGTCCSSAEPYTIIQVTTYAFLAVCASIATAM-----YVRKWIVKWRGGHAYMPLNQP---------------------IETQSPVAALASLAIIMTYFYLCDRTNFFMKENKYYSEFSFWIPVGYVFALGLFFTEDSK-------------------------------LTKVLHRDQTDELKGWMQIVILIYYMTGASHI-LPIYM-----------------HIKVLISGFLFLSGYAHFTYWWQT---------GNAG--LVRFLNVMFRMNFLTVILCLC-------------------------MNRPYQ----FYFFVPLLSFWYSIMYLMLSLPPRITAQSTEA-------------NPYQYLYVVIKFVTMLATVTVLYM-SE-----VFFERIFVTRPWKALFVTTDDDIHEWWYRWKLDRYTITYGMIFAAIFQISQ-----RFAVVDDNNHGNLFSKRISLTSTLAAITGIGCYMTWT-FFCRNRQ--------------DCEEVHSYVVFIPIVGYILLRNISGILRTRYSTFFAWFGKISLELFLCQYHIWLAADRNGVLVL--------L-PGFPTL----------NVLITSFIFVCVSHEIHRVTSVLLPYAVPNDW  LOC411555\_Amel\_66549058              LNTYCNDHMNF------------DDGSCCSYPEPATILQLLSLSILALCIIIGGGM-----WLYRKFCYYRTEISYLRVDMENTEEANNSETIQIEQPEV---QDFYTLMTSLALLSIILSYFYLCDRTNFFMKENKYYSEFSFWLPLGYILALGLFFTEDRER------------------------------GPRTLNREQTDEWKGLMQAVVLIYHVTGAKNV-LPIYM-----------------YLRLINSAYLFLSGYGHFCYFWQT---------GDVS--LIRFARVMFRLNFLTVSLCLC-------------------------MNRPYQ----FYHFVPLVSFWFLVIYFLAWLPPRIYSGSLNEY------------GPRALLYLALKLLGLVSMITILYM-SE-----VFFEKVFVTRPWKALFVTTDDDIREWWSRWRVDRYSVTWGVTFGAGLVALQ-----RI----DHIPGTALS-------SLLALISLTAYTTFT-ILCHSVS--------------ECEEIHSYVAFIPIIGYIALRNASLALRGKHSALLTGLGRISLETLVAQGHIWLAADSHGVLVL--------L-PRFPVL----------NLLVTSFIFICASHEIHRLTQVLAPYAVPNDW  LOC655926\_Tcas\_189235703             LNMYCNDYMNF------------NDGTCCSSTETYTTLQIVTFAILGICASIAIVM-----LCTRLIMKWRGRPIHEYSQLPDNAHQPPATP-----------GSYYNLFTSLAKMALIMSYFFLCDRTNFFMKENKYYSEFSFWLPIGYVTVLGLFFTEDSK-------------------------------YTKVLHRDQLNEWKGWMQLVILVYHITGASRI-LPINM-----------------HIKVLISAYLFLLGYEQFCCVWQR---------GDIG--IVSFFRVLFQLNFITVTLCLC-------------------------MNRPYQ----FYYFVPLLSFWYLMTYCFLAFPPHITAQTSEN-------------NVMQYFYLLIKFVVFFTVITILFM-SE-----VFFEKVFVTRPWKALFVTTDDDIREWWFRWKLDRYTIIYGMGFAVILLLAQ-----RYNIYDDNNHNNLFSRGLALTGILVAIAGIGCYLSIT-FLCSTEL--------------ECSEIHSYIVFIPIVGYIVLRNISGVLRTRYSSLFAWFGEISLELFISQYHIWLAADTHGVLVL--------I-PGYPVL----------NVMITSFIFVCASHEVHRLTKVLLPYAVPYDW  CG2938\_Dmel\_18543323                 WNMYCNDYMNY------------NDGTCCSSSEPYTTLQIVAYALFGVCMALVCGM-----CLRRWVLHLRGQTLYVPLQQQQSYDGGRAGGGSPSNALSALITDYGTPMVALSLLGLILAYFYLCDRTNFFMKENKYYSEFSFWIPVGYVFALGLFFTEDSR-------------------------------FTKVLNRDQTDELRGWILLVVLIYYMTGAQRV-LPIHM-----------------HIKLLISGYFFLTGYTHFTHMWQT---------GGSGSLFVRFFQAMFRANFLSVLLCFC-------------------------MNRPYQ----FYYFVPLLSFWLCIVYFVLALPPRISSASVDA-------------NPLHYLYLVCKCIGCLGGITVLFM-SE-----VFFERIFVTRPWKALFVTTDDDLHEWWHQWKLDRYTVAFGMIYAACFHIAQ-----KYNVFDDNNHGNLFSRRTSISVTLLALLGVGVYTSFS-FLCRNVQ--------------NCEEIHSYILFIPIVGYVVLRNISGILRTRYSAFFAWFGRISLELFVCQYHIWLAADRHGVLVL--------L-PGFPTL----------NMIITSFIFVCASHEVHRLTQILLPYAVPSDW  Lgig1000008388\_Lgig\_Lgig1000008388   INMYCNNDMNH------------NDGTCCNTPETTTPLQIVTFSVLLVCIVSAIAL-----FLVLSRLMVCFLLHSSMIYTENASE----------------------IFTSLAKLAIIMFYFYLCDRTNFFMKENKYYTHVNFFLPFAYVMILGFFFTENTE-------------------------------QTALLHRDQTDEWKGWMQLVILIYHLTGASKV-LPIYM-----------------HIRVLVSSYLFLTGFGHFTYFWTK---------GEYS--LFRFCQVLFRLNLLVICLCFV-------------------------MNRPYQ----FYYFVPLVSYWFLIMYITMAIWPHLTKTSAET-------------NTLHYIYMIVKFIILATCICLFYL-SE-----VFFEKVFLTRPFKGLFVTSDDSIHEWRFRWQLDRFSVLYGMIFAFAHQLLL-----KYKIVSDTSTGDLFSKPISTIVMAIGVAGIAGYTIFS-CFCRNKL--------------ECNEFHSYLTFLPIISYIVLRNVPGWLRTRYSSFFAWFGRISLELFIVQYHIWLAADTHGVLVL--------I-PSYPVL----------NVIITSFIFICVSHEMSKITNVLVKYAVPSDW  CASD1\_Ggal\_118085840                 MNVYCNKIMKP------------IDGSCCQPQPPLTLIQKLAFCFFTLSIIGYFV------ISLIHRNNYRKNKSCTDLEIGEEKKPTINTPSV---------STLEMLLHCFCKLGLIMTYFYLCDRANLFMKENKFYTHSSFFIPIVYILVLGVFYTENTK-------------------------------ETKVLNREQTDEWKGWMQLVILIYHISGASTF-LPVYM-----------------HIRVLVAAYLFQTGYGHFSYFWIK---------GDFG--VYRVCQVLFRLNFLVVVLCIV-------------------------MDRPYQ----FYYFVPLVTVWFMIIYATLAMWPQIVQKKAN--------------EIEIEVKSQFFMDY---SSEKSCH-LV-----GAFEKVFSFWPLSKCFEL-NGNVYEWWFRWKLDRYVVFHGMLFAFIYLALQ-----KHQMISEGKGDPLFSSRVSNVLLFISIVSFLTYSIWA-SSCKNKA--------------ECNELHPSVSVVQILAFILIRNIPGYVRSVYSSFFAWFGKISLELFICQYHIWLAADTKGILVL--------I-PGYPMF----------NVIVSTFIFVCVAHEISQITNDLAQIVVPKDN  CASD1\_Hsap\_40255037                  MNVYCNKILKP------------VDGSCCQPRPPVTLIQKLAACFFTLSIIGYLI------FYIIHRNAHRKNKPCTDLESGEEKKNIINTPV----------SSLEILLQSFCKLGLIMAYFYMCDSANLFMKENKFYTHSSFFIPIIYILVLGVFYNENTK-------------------------------ETKVLNREQTDEWKGWMQLVILIYHISGASTF-LPVYM-----------------HIRVLVAAYLFQTGYGHFSYFWIK---------GDFG--IYRVCQVLFRLNFLVVVLCIV-------------------------MDRPYQ----FYYFVPLVTVWFMVIYVTLALWPQIIQKKANG-------------NCFWHFGLLLKLGFLLLFICFLAY-SQ-----GAFEKIFSLWPLSKCFEL-KGNVYEWWFRWRLDRYVVFHGMLFAFIYLALQ-----KRQILSEGKGEPLFSNKISNFLLFISVVSFLTYSIWA-SSCKNKA--------------ECNELHPSVSVVQILAFILIRNIPGYARSVYSSFFAWFGKISLELFICQYHIWLAADTRGILVL--------I-PGNPML----------NIIVSTFIFVCVAHEISQITNDLAQIIIPKDN  LOC100045658\_Mmus\_149254923          MNVYCNKVVKP------------VDGSCCQPRPPLTLIQKLAACFFTLSIIGYFI------FYVIHRNAHRKNKPCTDLESGEEKKNIINTPV----------SSLEILLQSFCKLGLIMAYFYMCDRANLFMKENKFYTHSSFFIPIIYILVLGVFYNENTK-------------------------------ETKVLNREQTDEWKGWMQLVILIYHISGASTF-LPVYM-----------------HIRVLVAAYLFQTGYGHFSYFWIK---------GDFG--IHRVCQVLFRLNFLVVVLCIV-------------------------MDRPYQ----FYYFVPLVTVWFMVIYVTLALWPQITQKKANG-------------NFFWYLGLLLKLGLLLLCIWFLAY-SQ-----GAFEKIFSLWPLSKCFEL-EGSVYEWWFRWRLDRYVVFHGVLFAFIYLALQ-----RRQILSEGKGEPLFSNKISNFLLFVSVVSFLTYSIWA-SSCKNKA--------------ECNELHPSVSVVQIVAFILIRNIPGYARSIYSSFFAWFGKISLELFICQYHIWLAADTRGILVL--------I-PGNPTL----------NIIVSTFIFVCVAHEISQITTDLAQVVIPKDN  Casd1\_Mmus\_37620157                  MNVYCNKVVKP------------VDGSCCQPRPPLTLIQKLAACFFTLSIIGYFI------FYVIHRNAHRKNKPCTDLESGEEKKNIINTPV----------SSLEILLQSFCKLGLIMAYFYMCDRANLFMKENKFYTHSSFFIPIIYILVLGVFYNENTK-------------------------------ETKVLNREQTDEWKGWMQLVILIYHISGASTF-LPVYM-----------------HIRVLVAAYLFQTGYGHFSYFWIK---------GDFG--IHRVCQVLFRLNFLVVVLCIV-------------------------MDRPYQ----FYYFVPLVTVWFMVIYVTLALWPQITQKKANG-------------NFFWYLGLLLKLGLLLLCIWFLAY-SQ-----GAFEKIFSLWPLSKCFEL-EGSVYEWWFRWRLDRYVVFHGVLFAFIYLALQ-----RRQILSEGKGEPLFSNKISNFLLFVSVVSFLTYSIWA-SSCKNKA--------------ECNELHPSVSVVQIVAFILIRNIPGYARSIYSSFFAWFGKISLELFICQYHIWLAADTRGILVL--------I-PGNPTL----------NIIVSTFIFVCVAHEISQITTDLAQVVIPKDN  LOC678772\_Rnor\_109473087             MNVYCNKILKP------------VDGSCCQPRPPLTLIQKLAACFFTLSIIGYLI------FYVIHRNAHRKNKPCPDLESGEEKKNIINTPV----------SSLEILLQSFCKLGLIMAYFYMCDRANLFMKENKFYTHSSFFIPIIYILVLGVFYNENTK-------------------------------ETKVLNREQTDEWKGWMQLVILIYHISGASTF-LPVYM-----------------HIRVLVAAYLFQTGYGHFSYFWIK---------GDFG--IHRVCQVLFRLNFLVVVLCIV-------------------------MDRPYQ----FYYFVPLVTVWFMVIYITLALWPQVTQKKANG-------------NGFWYLGLLLKLAFLLLCICFLAY-SQ-----GAFEKIFSLWPLSKCFEL-EGSVYEWWFRWRLDRYVVLHGALFAFIYLALQ-----RRQILSEGKGEPLFSNKISNFLLFVSVVSFLTYSIWA-SSCKNKA--------------ECNELHPSVSVVQIVAFILIRNTPGYARSIYSSFFAWFGKISLELFICQYHIWLAADTRGILVL--------I-PGNPTL----------NIAVSTFIFVCVAHEISQITADLAHVVIPKDN  CASD1\_Ptro\_114614562                 MNVYCNKILKP------------VDGSCCQPRPPVTLIQKLAACFFTLSIIGYLI------FYIIHRNAHRKNKPCTDLESGEEKKNIINTPV----------SSLEILLQSFCKLGLIMAYFYMCDRANLFMKENKFYTHSSFFIPIIYILVLGVFYNENTK-------------------------------ETKVLNREQTDEWKGWMQLVILIYHISGASTF-LPVYM-----------------HIRVLVAAYLFQTGYGHFSYFWIK---------GDFG--IHRVCQVLFRLNFLVVVLCIV-------------------------MDRPYQ----FYYFVPLVTVWFMVIYVTLALWPQIIQKKANG-------------NCFWHFGLLLKLGFLLLFICFLAY-SQ-----GAFEKIFSLWPLSKCFEL-KGNVYEWWFRWRLDRYVVFHGMLFAFIYLALQ-----KRQILSEGKGEPLFSNKISNFLLFISVVSFLTYSIWA-SSCKNKA--------------ECNELHPSVSVVQILAFILIRNIPGYARSVYSSFFAWFGKISLELFICQYHIWLAADTRGILVL--------I-PGNPML----------NIIVSTFIFVCVAHEISQITNDLAQIIIPKDN  CNN01530\_Cneo\_58262436               LSWRCNDVMRH----------EGATGTCCKRYDWVTPIQGLILA-VLILWAPL--------GTFITPRLPPNSPILDYLPAT-S------------------------IAPALSTFGLAMGYLFLADRTHVFQKEQKDYDAVIFGMITLAAFVAGLLTIKNSGK------------------------------DLGFLNRDITDEWKGWMQIAILIYHFFGASKI-SGIYN-----------------PIRVLVASYLFMTGYGHFFFYYKK---------ADFG--FQRVVMVLVRLNLLSVVLPYT-------------------------MNTDYA----FYYFAPLVSWWYLIIYATMAIGSKYNDRPA---------------------FLLTKLFTCAGLVTLFMH-FP-----WLMEDVFKV--LNTVFNI-QWSAKEWSFRVTLDLFIVWVGMLCAYGFVKFN-----EHQISDRPWFPVMRTAT-----LVGSVLGMIWYFWFE-LHLASKF--------------VYNEYHAVVCIVPIMSFVFLRNASPVLRSSTSKIFCFIGQCSLETFILQFHGWLASDTKAILLA--------V-PSTQWR--------PVNLVISTICFIWLSYRVSGATGEITEWLVGKKK  consensus/100%                       ...................................................................................................................h.....h.a.sDps..h.K..K.a....F........................................................................................-----------------........................---------...............bhNhhsh.l.h.-------------------------Ms.sY.----.Ya.sPhhohWh.h.a..h.h..p...............................p..........h.............p..F....h...h........pW.....lD.h......................................................................----------..........................................................................................................................  consensus/95%                        bs.bCN.............................................................................................................hs....hhahsDRs.hh.K.pK.a....F.h.....h.h.....p....................................................................sl..-----------------.hplh.u.ahF..ua.p...hh..---------.s.u..h.ph..s.hRhNhhsh.Lsh.-------------------------MspsYb----.YahsPLhohWahlha..h.h..p.............................h.ch.........hh............p.hF....h...h...p.ph.pW..bh.LD.h....Gh..uh....................................u......a................----------.h...ps......l.sa..hRN.....R...S..h..hGp.SL.....Q.H.hhA.Dppu.L.h..........................s..l.s.hhlh.s..h................  consensus/90%                        hNhbCN..h.................pCC....................h.................................................................hsh.h.hhahsDRs.hh.K.pK.ap...F.h....hh.hs.h..pp.....................................L.b.b.pEh+Ghhb..........s.....sl..-----------------.h+lhlu.YhFboGasH..ahh.p---------.shu..h.+hh.shhRhNhhsh.Lsh.-------------------------MspsYb----.YaFsPLlohWahlha..hhh..ph............................h.Kh......h..hh............p.hF....h...F...p.ph.-W.bbh.LD.a....Gh..uh...........p........................u.hs...a.........p......----------.h...Hs.h..h.l.ual.lRNh....Rs..S.hhsahGphSLE.h..Q.HhhLAuDppGlLhl........l.................sh.l.s.hFlhhs..h...o..h...h.....  consensus/85%                        hNhbCN..h...............s.oCC......p.hp...h..h...h............................hp...................................huhhh.hhahsDRs.hahK.pK.ap..pF.h.h.hhhhlGhh..cp....................................hLpRpQT-EWKGWMQ..........App...slY.-----------------.l+lhlusYLFboGauH..aah.p---------s-hu..h.RhhpVhhRhNhLshhLshs-------------------------MspsYb----.YYFsPLlohWahllY..hhl.sphs...........................h.Kh...h..h.hhh...p......hhc.lF....hp.hF...p.sh.EWbaRhpLDba.hh.Ghh.uh....hp.....p..h..c..................ulhs...a..h......sp.....----------.hp.hHs.hshl.IhualhlRNh.s.hRs..SphhsahGphSLE.al.QaHhhLAuDocGlLll........l..s...b..........shhl.s.hFlhhua.h...T..h..hh.....  consensus/80%                        hNhbCN..h...............s.oCCp.....s.hp...h..h.h.h..h...................p.s...hp................................h..hulhh.hhYhsDRsphahKppK.as..pF.h.h.hhhhlGlh..cp.................................p..hLpRcQT-EWKGWMQhhlLlh.h.GApp...slY.-----------------.lRllluuYLFboGaGH..aah.p---------sDhu..h.RhhpVhhRhNhLslhLshs-------------------------MspsYb----.YYFsPLlohWahllY.shhl.sphs...........................hhKh...h.hl.hhh...p......hhc.lF....hp.hF...p.sh.EWbaRhpLDba.lh.Ghh.uhh...hp.....p..h..c.................hulhsh..a..h....p.sc.....----------.hp.hHs.lshl.IhualhlRNhss.hRs..SphhuahGphSLE.Fl.QaHhaLAuDocGlLll........l..s...b..........shhlss.lFlhluapl..hTs.l..hh.....  consensus/75%                        hNhbCNp.hp..............c.oCCp.....o.lQ.h.hs.h.h.h..h..........b........p.s...hpss...........................h..h.phulhhhYhYhsDRophFhKppKbas..sFhl.hhhhhhlGlh..cp......c..........................p..hLsR-QTDEWKGWMQhhlLlYahoGASph..slYh-----------------.lRlLVuuYLFbTGYGH.saah.+---------uDau..h.RhhpVhhRLNhLslhLshs-------------------------MspsYb----FYYFsPLVoaWFhllYhshAl.sphs..p....................h...hhKhh..h.hlshhh...p.....shh-.lF....hp.hF...p.slpEWbFRhpLDba.lh.Ghlhuhh...hp.....c.pl.scs......p.........hulhuhh.Y.ha....p.s+.....----------phsphHs.luhl.IhualhlRNhss.hRs.aSphaAahG+hSLE.FI.QaHlWLAADT+GlLll........l.ss...b..........NlllsohlFlhluHcl..hTs.ls.hh.....  consensus/70%                        hNhbCNp.hp..............DsoCCps....o.lQ.h.hs.h.l.hhsh..........b........p.s..shpss...........................h.sh.phulhhhYhYhsDRTphFhKpsKbao..sFhl.hhhhhhlGlh..cp.....sc..........................p..hLsR-QTDEWKGWMQhhILlYHhoGASph.lslYh-----------------.lRlLVuuYLFbTGYGHhsaFhpK---------uDau..h.RhhpVhhRLNhLslhLshs-------------------------MspsYb----FYYFsPLVoaWFhVlYhThAl.sphsppps...................h...lhKhhh.h.hlshhh..pp.....shh-blFsh..hp.hF...p.slcEWbFRhpLDba.VhhGMlhAhh...hp.....c.pl.scs..s.h.p.........hulhuhh.Y.ha....h.s+.....----------phsphHshluhlPIlualhlRNhsshhRs.aSphFAWhG+hSLE.FIhQaHIWLAADT+GlLll........l.Pu...b..........NllloohlFlhluHcl.phTs.ls.hh.....  Ccel\_2617\_Ccel\_220930016             ----------------------------------------------------------------------------------------------------------------------------------------------SIYVILAVILLFGAKVRIRGW-------------------------------DDGFLSLSNTKIIQGFCAVLIIIHHISQIITDSQALSPF--------------ADYGVLFVGIFFFCSGYGLIKSYKTKYSY-----LKGFV--GRRLPSVLVPFY-VTTLIY---------------------MAVFLALNPKPS----LLQVFLNLTGIQLINP---QAWFIVT---------------MVIFYLAFYFIFNYLKNEKIAFVSMAIFIIFYTILSLIL---------RHGPWW-LQG--EWWYN--TCF-LFYVGMIIARIEKGLIVKVK-KYYVLLLPSVIAALIVMFKVSVYTLDKFS-----YYAQTAIASGYPESL----------ICFSTQLPAVILFVSSIFILSMKITFS-N---MILRFLSKISLELYL--IH-YLFLLLYKSEIINISNNLLYVTM-VVISSVAAAFLLHIINRTLIGLITPKGK------------------  SAB2441c\_Saur\_82752149               ----------------------------------------------------------------------------------------------------------------------------------------------------------------------------------------------------SPRYLPGLDGLRAFAVIGIIIYHLNAQW-----------------------LSGGFLGVDTFFVISGYLITSLLISEYYRTQKIDLLEFW--KRRLKRLIPAVLFLICVVLTFTLIF---KPELIIQMKRDAIAAIFYVSNWWY----ISQNVDYFNQFAI-EP-LKHLWSLAIEEQF-----------YLLFPLVITFLLHRFKPRNIIQTLFIVSLISLGLMIVIH---------FIT-GD-NSR--VYFGTDTRLQ-TLLLGCILAFIWPPFALKKD--ISKKIVVSLDIIGISGFAVLMTLFFIVG-----DQDQWIYNGGFYIIS----------FATLFIIAIAVHPSSLFAKFLSM---------KPLLIIGKRSYSLYL--WHYPII-VFVNSYYV-------Q------GQI---------PVYVYIIEILLTALMAEIS---YRFIETPIRK  nodX\_Retl\_190891955                  ---------------------------------------------------------------------------------------------------------------------------------------------------------------------------------------------------------PGFDFLRIGLAFSIVLTHSFLLTRNDAFIRGSIFW-----------FTEYA-LVPMFFALSGFLIA-------GSAQRLSLRNFL--INRGLRIVPA---LAVDIVVCSLII---GPIVTIVYHSEYFTDPRFVKYFLNIVGWIHYELPGVFQDNPSQKVNGALWTVPWEIFC-----------YIIMSFLMITAIVKTRYKLLAVTAAYIVI-GLIVQKM-----------PYLLPDTIKPRFLFMSRGSQLITAFLMGIVAFQFKA--------------VIPHSRWIFAAACLVCVAAILT------------LDSRAIESV----------INRPLVITSLVYMTVFIGLSEVPI--------PAFFRKGDYSYGVYL--YHDPFLQIWISAFPS-------VFLYPKYGAL---------ALYAVGLPSALA--VAVLS---WHFIEKPI--  gumF\_Xaxo\_21243313                   ------------------------------------------------------------------------------------------------------------------------------------------------------------------------------SVSAMPASPSALPAANARVATSGRVRDPRIDATKAIAILLVVFCHAKGVPHGMTL------------------FAYSF-HVPLFFLVSGWL-----------AAGYASRNTGL-TQTISKQARS---LLLPYVTFYLLG---YAYWLLTRNIGEKAARWGSHPWWEP------IVAMFTGIGPDLYVQPPLW-------------------FLPVMLATVVSYLVLRRLMSPILIAGLALIVAWFWMN-----------WFP-AQ-YVR--IFFGLDVLPV-SLCFYALGALLIYLSPRLPT-------SLAGSALATVVLALAVAWLAVA--NGRIDVNMLEFGHDHALFL----------VSALLGSLMVICAARLVQ---SW---------AWLQWIGRNTLLILC--THMLVF-FVLSGVAA-------L---A--GGFGGARPGLG---WAVFVTLFAL--AASVP---LRWILMRFAP  mdoC\_Ecol\_15801164                   ------------------------------------------------------------------------------------------------------------------------------------------------------------------------------------------------MNPVPAQREYFXDSIRAWLMLLGIPFHISLIYSSHTWHVNSAEPSLWLTLFND--FIHSF-RMQVFFVISGYF-------SYMLFLRYPLKKWW--KVRVERV------GIPMLTAIPLLT---LPQFIMLQYVKGKAES------WP----GLSLYDKYNTLAW-EL-ISHLWFL-----------------LVLVVMTTLCVWIFKRIRMVKLSVIFLCLGIGYAVIRRTIFI--VYPPILS-NG-TFN--FIVMQTLFYL-PFFILGALAFIFPHLKAL---------FTTPSRGCTLAAALAFVAYLLNQRYG--SGDAWMYETESVITM----------VLGLWMVNVVFSFGHRLLNF-QS---------ARVTYFVNASLFIYL--VHHPLT-LFFGAYITPHITSNWL------GFL------CG-LIFVVGIAIILYEIHLRIPLLKFLFSGKPVVK  gumG\_Xaxo\_21243312                   -------------------------------------------------------------------------------------------------------------------------------------------------------------------------------------------------TAPALTRDWQIDAAKALAIALVVLGHASGMPPAYKM------------------FAYSF-HVPLFFVLSGWV-----------GERFGHRALT--AASVAKLART---LLVPYLAFFMVA---YALWLLTAAIDGHAGHPQTRPWWHP------LTGLLWANGSRLYVLPALW-------------------FLPALFVTTLAYIALRARLSAAAVATISLPLALAWA-----------GWFP-SL-QQR--LPLALDVLPV-ALFFIAFGGWLSRFADAL-R-------SLRAQVWALALLPLAALWWWLAGWNGQVDVNNLQFGQSAAIFL----------LVSLLGTAMTFCVAYFIR---RL---------RWVQWIGTNTLLILC--THTLVF-LVTTSVVA-------R---T--GLI--ARSAIGTPAWALGLSAFAI--AASVP---MRAVLVPIAP  opgC\_Rsph\_77463700                   -------------------------------------------------------------------------------------------------------------------------------------------------------------------------------------------------PPRVAGRDKRLDFFRGLALVMIFINHVPGTVWENFTSRN---------------FGFSD-AAEGFVLMSGIAAGLAYSKGFWTPTWAAARKIW--RRAWTLYL-----VHLLTTFWAIAI---SAAFALWFAAPDLLHQNQVWILFQKPLGFLIGVPALTHQLGYTN-ILPLYATL----------------LLVTPALILAALRFPRTLLVGSVLLWALTGQFRLNL-----------PNFP--N-PGG--WFFNPLAWQI-IFVMGLLTGVALKKGKRFVP-ILP---WLQALTAAYLLLALIWLKVPVV--YDMMNHFMRVLNRDLGLPFYITAFDKT--FATVPRLLHVLALAYLLSTLGWVR--------HLAASGWAEPFALLG--RHSLQV-FATGTILCFALQGVKDL--T--GDQ---------FLFDTLLLAMGL--SVQFG---VAWLADRWKG  beltless\_Dmel\_85724824               ---------------------------------------------------------------------------------------------------------------------------------------------------------------------------------WFTAFSLDKNLRWLFSTSSAPGDIEAVHGIRFLNAIMLIFSHKSMAMFFNPYNNRTAMSESLGQPWTVIGRAASL-YTDPFLLFSGMLTSYSLFGRLMKQQPIRLKNEY--ISRLMRIVPPLA-ALILFCTYVLPL---WGSGPQWNLVVGHHADICKKNWWR----NLLFIHNYFGFSE-MC-LTHTHHLGIDTEL-----------FAVAPLLILALWRWPRRGLFALLLLCTVGTAARYYT-----------TIVN-QL-SNY--IYFGTNIQRL-VYIMGILLGYVLRKY--QNA-RL----SSLQLRLGWLVATVCVLASLLG--PAPMGDINYVYNSTHAAIYAAFAPIAWCLFFSWIVFVSHNGYTNKLTKLFAW---------RGFQVSTKLSYAIYL--TQFPVF-FFNVGRRR-HIHHY-Y------NFV-SIILDTN-EFISIFLASVALTVLFDAP---FQNLKKLLIK  FRAAL0973\_Faln\_111220440             -------------------------------------------------------------------------------------------------------------------------------------------------------------------------------------------------AAAKFAYNPALDGLRVVCIYIILAGHMGAIH-------------------------ASNVAVDVFFVLSGFLITALLLAERSRTGTISLGRFL--VRRAYRLMPAMWVYLLVGLAVTVAFKWNDVPFRDDYIGSALSAFFNVNNWYK----VVHPAAGGRW-------LAHVWSLSLEEQFYL---------LWPGLFLLVSRSARLRPHLIKILLAMIVVCAVWTYT-----------VASGGAP-HSR--VYLALDTHVA-PLLIGCLLAVWRDARLRALAEEEPVADPPTGLLAGIGLILFAFLGPNKDTH----EANWLDHAAYIPSAL----------LGAIVILSADLRRDTAWVRLLGS---------PRMAFLGKITFSIYL--WHYPVI-SA----------------------------------------------------------------  yrhL\_Bsub\_16079768                   -----------------------------------------------------------------------------------------------------------------------------------------------------------------------------------------------------HRYIPGLDGLRAFAVLSVITYHLNFNW-----------------------ANGGFIGVDIFFVLSGYLITSILLPAYGNDINLDFRDFW--VRRIRRLLPAAYLMIFSTVVWVVLF---DRELLHTVRGDAISSLFYMSNWWF----IFHKLSYFDSFGSPSP-LKNLWSLAIEEQFYI---------IWPMFLVVGMYIMKSRARLAAVISLLVLCSAVMMSV-----------LYEPGGD-PSR--VYYGTDTRSF-ELLIGCALALVWPMKRLSSN-RLPSKLKHTLHATEFLAFCILVLCVYFTD-----EYEPFLYRGGMLFIS----------VTAAILIACVCHPSSFLGNLLSW---------RPLRWLGTRSYGIYL--WHYPVI-VLSTPVQE-------I------GNP---------VFWHIVLKVIVTCILAELS---YHFIEKPIRT                                       ..........................................................................................................................................................................................................................H..........................................oGa........................................................................................................................................K.........................................................................................................................................................................................H.......................................................................  The first, second, 5th and 9th helices respectively contain: 1) a conserved histidine; 2) a [ST]Ga signature (where a is an aromatic residue) which might bind the nucleotide of the acyl coA; 3) a conserved basic residues (K or R); 4) A second conserved histidine. |

  
  
  
  
  
Top  


---

Multiple
Alignment of Cadherin-Like domain  
  

|  |
| --- |
| RES                                     ENTPCHIKQIFTHPHLELNPDFH-PKIKDYYCEVPFDVVTVTIGVETPKCL-----CKVHLYE-----------------QAGPSF-----------ASYPL-GLGM--NKISIFVVDESPAH-------------------------GETLITYKLTIYRE  ALIGN                                   -----HHHHHH------------------EEEEEE---EEEEEEEE----------EEEEEE-----------------------------------EEEEE-E------EEEEEEEE---------------------------------EEEEEEEE---  HMM                                     ----------------------------EEEEEE-----EEEEEEEE---------EEEEEE------------------------------------EEE--------EEEEEEEE----------------------------------EEEEEEEEE--  FREQ                                    -------EEEE----------------EEEEEEE---EEEEEEEE------------EEEE-------------------------------------EEEE--------EEEEEEEE---------------------------------EEEEEEEEE--  PSSM                                    ---------E------------------EEEEEE----EEEEEEEEE---------EEEEEE-------------------------------------EE-----E--EEEEEEEE---------------------------------EEEEEEEEE---  FINAL                                   -------EEE------------------EEEEEE---EEEEEEEEE----------EEEEEE------------------------------------EEEE--------EEEEEEEE---------------------------------EEEEEEEEE--  DKFZp451J1618\_Hsap\_30268329             ENTPCHIKQIFTHPHLELNPDFH-PKIKDYYCEVPFDVVTVTIGVETPKCL-----CKVHLYE-----------------QAGPSF-----------ASYPL-GLGM--NKISIFVVDESPAH-------------------------GETLITYKLTIYRE  NEMVEDRAFT\_v1g244124\_Nvec\_156378255     PGAIPVLTNIHTHPHMELQPAFR-HDTEAYQARVPFHVLNVRLWGKTSTCL-----SEARINS-----------------YRGTAQP----------ANYSL-GVGW--NAFALHLMDTSHAK-------------------------AWNLMSYKLHIYRE  DKFZp451O0317\_Hsap\_30268303             ENTPCHIKQIFTHPHLELNPDFH-PKIKDYYCEVPFDVVTVTIGVETPKCL-----CKVHLYE-----------------QAGPSF-----------ASYPL-GLGM--NRISIFVVDESPAH-------------------------GETLITYKLTIYRE  mCG\_123947\_Mmus\_148681901               EDVLCHIKKIITHPRLELSPEFN-PKIKEYYCEVPFDVLTVRIGAETSKCQ-----CKAHLQE-----------------QAGPSF-----------ATYPL-GIGM--NRISVLVVDESHAG-------------------------GAALTTYKLTIYRE  LOC500046\_Rnor\_109473163                EEAQCQIKKIVTYPRLELSPEFN-AKIKDYYCEVPFDVLTVRIRAETSKCQ-----CKVHLQE-----------------QTGPSF-----------ANYPL-GLGM--NRISMLVVDESRAE-------------------------GDALTTYKLTIYRE  LOC472491\_Ptro\_114615647                KNTPCHIKQIFTHPHLELNPDFH-PKIKDYYCEVPFDVVTVTIGVETPKCQ-----CKVHLYE-----------------QAGPSF-----------ASYPL-GLGM--NKISIFVVDESPAH-------------------------GETLIMYKLTIYRE  LOC100012920\_Mdom\_126340617             KDTLSHIKQIFTNPHLDLNPEFN-PNIKDYYSEVPFDIITIKIGAETSNCQ-----CKIHLDE-----------------KAGPSF-----------ANYPL-GLGM--NKILILVVDESQVN-------------------------GEVLSIYKLTVYRE  LOC100071386\_Ecab\_149706163             RDTQCHIKQIFTHPHLKLNPEFN-PKIKDYYSEVPFDVVTVTIGAETSKCQ-----CKVYLRE-----------------RAGPSF-----------GSYPL-GLGM--NKISMLAVDESPAQ-------------------------GETLITYKLTIYRE  LOC417759\_Ggal\_50728144                 KDTLSHIRQIFTSPQLDLNPQFN-PKIREYYAEVPFDVVTVKIGAEPSNCQ-----CQVHLDE-----------------VKGPSI-----------ANYPL-GLGL--NKIVVLVTDDSQPS-------------------------PQVVSSYKIKIYRE  CH211-152M4.1-001\_Drer\_122891352        KCVDSHLRQLYTDPPMVLMPAFS-AWVKEYRAEVPFDVITIQIRPEPVMPH-----CHIHLDE-----------------HRGPRM-----------ANYPV-GLGN--SRINILVMDESEAE-------------------------PVVMTIYTLNIYRE  GSTEN:00005234:G:001\_Tnig\_47211695      ------LRQIYTDPALTLAPPFR-PGVKVYRAEVSFETVTVRIRAEPQSPG-----CRVHVDG-----------------PRGPRT-----------VTYPV-GLGD--NTVRVLVTDDRGPQ-------------------------PVVVATYTLSLRRE  Swol\_0169\_Swol\_114565738                NNTGNANLNSLTLSNGTLSPAFD-PGITSYTVNVDNTVSSISITPTAAAAQ-----AAIKVNGSPV--------------SSGSLS-----------QAIAL-NPGN--NTIAILITAQN----------------------------AAT-RTYTITINRA  GeobDRAFT\_1350\_Gsp\_110601032            VLSNNADLGSMTISAGELTPAFS-STVTSYTAAVTNGTSSITVTPTLADAN-----AEVTVNGVEV--------------ASGTAS-----------SPISL-AVGD--NTISIRVTAQD----------------------------EVTTKTYTIVVNRA  GeobDRAFT\_1350\_Gsp\_110601032\_2          GSSTNANLSNLALSSGTLTPAFA-SSTTSYTASVANTVTSITVTPTEEDTT-----ATTTVNGVPV--------------ESGTAS-----------GPISL-AVES--NTVTVVVTAQD----------------------------GVTKKTYTVVVTRA  GeobDRAFT\_1350\_Gsp\_110601032\_3          APSTNANLSNLTLSTGTLTPAFA-SGTISYTASVTNSTTSLTVTPTAQDTT-----ATIKVNGITV--------------ASGVAS-----------AAIPL-AVGS--NTINTVVTAQD----------------------------GSTTTTYTVVVTRA  GeobDRAFT\_1350\_Gsp\_110601032\_4          APSTNANLSSLTLSNGTLIPAFA-SGTTYYTASVTNGTTSVTVTPTVQDST-----ATVTINGVSV--------------ASGTPS-----------GAIPL-SVGS--NTINTVVTAQD----------------------------GSTTKTYTVVVTRA  GeobDRAFT\_1350\_Gsp\_110601032\_5          APSTNANLSNLALSAGTLTPAFA-SGTTSYAASVSNATSTITVTPTVQDAT-----ATITVNGVAV--------------TSGTAS-----------VPFAL-VVGA--NTLNTVVTAQD----------------------------GATTKIYTVMVTRA  GeobDRAFT\_1350\_Gsp\_110601032\_6          AASTNANLNNFALSSGTLSPAFA-SGTTSYTASVSNATSTITVTPTAQDST-----ATIKVNGVAV--------------TSGTAS-----------VPFAL-VVGA--NTLNTVVTAQD----------------------------GTTTKTYTVVVTRA  GeobDRAFT\_1350\_Gsp\_110601032\_7          APSTNTNLSNLVLSAGTLSPSFA-SGTTSYTASVTNATSSITVTPTVQDTT-----ATIKVNGATV--------------ASGSAS-----------GAITL-TVGS--NTIDTQVTAQD----------------------------GTTIKTYTVVVTRA  ESA\_pESA3p05539\_Esak\_156936658          TLSSDATLSGLSFSGGALSPGFS-ASNTSYHATVDYAVSSVTLTPVTTHNA-----STVTVNGNVV--------------SSGSAS-----------PSVNL-SVGT--NTLTIVVTAED----------------------------GTT-KNYSVVIQRN  Cbei\_4725\_Cbei\_150019535                TKKGEAKLQSLVPSTGTLSPAFN-SSTYDYTLQVPTAQTTIAFTPTAVDNS-----STIKVNGVTV--------------KSGSRS-----------QNIKL-DEGE--NDVEVIVTTKD----------------------------GDT-NTYNIKVTRT  Cbei\_4725\_Cbei\_150019535\_2              ALFRSSQLIGLTLTSGTLTPAFN-KGIYEYSGTVDNSVTSIGVTPTAEDVN-----ATITVNGKKV--------------PSGATS-----------PYISL-DEGG--NTINVKVTDSK----------------------------GNS-NTYVLNITRR  Cbei\_4725\_Cbei\_150019535\_3              YPKDNVNLASLSVTDGTMSPKFD-PETYLYSVKVARNIEKVRVMYTSQNDK-----AKIKINGKEY--------------TNGQ-S-----------DYIKL-DIGA--NLVVVEVTAED----------------------------GKTTTTYKLSVIRG  Pjdr2DRAFT\_0650\_Psp\_169185381           MGISYPKNDANLSGLTASAGALTKVDQTSYTMDVPNSVSQLTLTPTAESEFA----REITVNGNKV--------------ASGEAS-----------PSLSL-VDGE--NAITVVVTAED----------------------------GVTTATYQVTVNRT  Pjdr2DRAFT\_0650\_Psp\_169185381\_2         PAAVDNTLQSLTVSEGTLAPAFS-PEVTEYTVSVPFSVSELSVTPTSSAAS-----ATLKLNNFST--------------PNGQPS-----------ANVPL-QVGE--NTITVNVASAD----------------------------GTNFLDYTLVVTRE  Cbei\_4765\_Cbei\_150019574                AEEDREYAYLDGIYLSDGSVDFS-KNKTSYDVNVGEDVDKLTVRANPDDDD-----DYIEINGNSVY-------------EDDNFE-----------KTVNL-DKGN--NTITIYVEHED----------------------------ED--TTYTLNVYRG  Bsph\_2088\_Lsph\_169827635                SLSNNADLSNLTLSTGTLSPAFA-SGTTDYTANVASNVGNIQVTPTLADSY-----ATLTLNGAAV--------------TSGIG------------KDIAL-NVGL--NTITIIVTAQN----------------------------QTT-KIYTITVTRA  STH2197\_Sthe\_51893335                   SKSTVAELVDLSINPGRLQPEFR-PDRTTYSATVPHEVTEVAVEYQAGDAW-----AKVTVEG-----------------------------------NKDL-KVGT--NRITVTVLAES----------------------------GDK-RVYTINLTRE  Amet\_3803\_Amet\_150391531                IPSGNAKLMELLLSQGTLNPTFS-EDIVSYSANVGSGISSMNVTTVIEDSN-----ATVAINGNSV--------------ASGSV------------IPISL-NTGN--NTITIVVTAEN----------------------------GSI-KTYTIIVNRA  Cbei\_4726\_Cbei\_150019536                YAEQDNPSLKDIYLSDGGNLDFS-EDKYSYVTDVGNDIDQVLIKAKPYDLL-----DTVKVDGEIV--------------TRDDSYR----------KVVPL-VKGK--NKVEIEVLDNR----------------------------SDATSTYNVYIYKG  Cbei\_4726\_Cbei\_150019536\_2              YKGGKDAVYLKDININDSNIGFD-KNTNFYNIELDEGTNMMELEATPEDGN-----YSMTANGKQL--------------SNDSIK-------------VKFNGIGK--YTINLGVKDDD----------------------------TQRIGNYTLNMYLG  LOC580673\_Spur\_115762561                --MDDADLEKLSIKPGSLNPKFN-RNTTEYNVTVASDVGKLTISCLTSDSN-----ASYQIVG-----------------GDGGRT-------------VDL-AEGKT-TTIKIDVSAED----------------------------GTS-KLYIIHAKRL  LOC580673\_Spur\_115762561\_2              LSATDASLSGIKIADGCLVPDFD-PDTLNYSCLLPYTASALKVTPTAPDKK-----NAVKVNG-----------------QDPGSE-------------VNL-NVGD--TAVEIEVTSAD----------------------------GSNKQTYTVNATKK  Cphy\_3465\_Cphy\_160881587                AASKNTNLAILKVSPGTLTPSFK-ADEKEYSIMVGPETEKIIISAIPEDTD-----ATVGIKG-----------------------------------NEAL-KIGN--NTIVITVTAQS----------------------------GNT-GEYRIQVLKE  Cbut5\_010100004686\_Cbut\_168189450       EDTTQDPVYLKGLSLNKGDITFL-KQRTSYNIKVASSVNEIKITAEPEDDS-----SRVRIDGSLV--------------DEDDNYR----------KTISL-DKGK--NEIKIKVTDDK----------------------------DNQ-RVYTLNITRG  Cbut5\_010100004686\_Cbut\_168189450\_2     DGDSQGDVHLSNLELDEADLEFK-EDKTSYEVNVDEDVSKILVTAEPEDEE-----YLVTINGSEV--------------NSGDEYE----------KKVSL-SKGK--NTITVVVQDEVE---------------------------DTK-RTYKITVNRG  Cbut5\_010100016489\_Cbut\_168191789       TKKGVAKLKSLVPSTGSLSPSFN-SDTTEYTVTVPTTQETIAFTPTAVDNS-----STIKVKKITV--------------ASGKKSQ-----------DIKL-NEGE--NEIPIVVTNKD----------------------------GDV-TTYKVTVTRT  Cbut5\_010100016489\_Cbut\_168191789\_2     EKFRSANLKSLKLTSGTLSPTFN-KGVYQYTTIVENTVSSVGVTPVAEDAN-----ATIEVNGKEI--------------PSGATSP-----------YINL-DEGG--NVINVTVTDSK----------------------------GND-NTYVIMITRK  Cbut5\_010100016489\_Cbut\_168191789\_3     YSKNNVNLSSLSVTDGTFSPKFD-PEIYAYSVKVPRNIEEVRIKFETQNEK-----SKVTINGVEY--------------KSGQQSD-----------KIKL-DLGA--NTVVVKVVSED----------------------------NKSTVNYTLSIIRD  HM1\_0140\_Hmod\_167628271                 NANLGELTVTVGGKQRNLIPELN-DTDTSYTAYVPYNTNLVTIEAKPQSPG-----AKVRFWGL----------------DTNRYT-------------EDL-NGYN--MLVPITVWAED----------------------------GTM-KEYQLSVLQG  HM1\_0140\_Hmod\_167628271\_2               PSIKLRSLSSPNDSLYFSPDQFS-PDQNNYVINVPLYRTDIEFVPVLDHIG-----ATLKVNGVPW--------------SSGQTF------------KAPL-NVGE--NIITFEVGDYM----------------------------DT--NTYTVKVNRA  HM1\_0140\_Hmod\_167628271\_3               APSSDATLAYLSVNQGTLDPAFD-KNTTSYTVSVGEDVASIAIYADVNEPH-----ATATLYK-----------------VDGNQREELQSQLYGIRKTVEI-QPGE--NSFEIVVTAQD----------------------------GTTRETYSLNVFRT  HM1\_0140\_Hmod\_167628271\_4               MGLESVRFVYKDQSGNDQTVVPS-SGTDGFHAEVPSYVDHIKVYATPEYTTGNNP-AYTFVNHMID--------------YAGSTVFVDHSVPG---VTVSL-GVEM--NTIPITVVAAN----------------------------GNT-STYNLYVTRP  HM1\_0140\_Hmod\_167628271\_5               DPNSGLQVSIDKNSIESLNPVSY-PFNLAYTVNVGSEVDKIYLRAKPWYPG-----ASLTINGATAEYQ-----------YDDVNSPYPVQYCEW--REVPL-SAGA--NKVEVAVTAPN----------------------------GKNTKTYTVTINRA  HM1\_0140\_Hmod\_167628271\_6               EPPLSSNASLSGLTLSAGSLSFA-PDTVSYTLSVANGVTSTTVTPVAADAN-----ARVQVNGKDV--------------ESGRSSE-----------PIAL-KVGS--NTIAIVVTAQD----------------------------GVTVRTYTVTVNRA  CLOBOL\_00465\_Cbol\_160935575             SASKDATLKSLQISPGSLTPAFS-PDVDTYAVTVGTDVDKVIVSADCTDEN-----ATKVVSG-----------------------------------NEGL-QMGE--NRVTCRVTAQD----------------------------GETIKEYVIVVTKA  Cbut5\_010100004701\_Cbut\_168189453       EEDDVEYPYLSSIYLSDGSINFT-KSKTSYKVNVDEDVKQLLVRAKPQDDD-----DLVEINGDSVD-------------EDGDYEK-----------KVSL-DKGE--NTIEITVENDD----------------------------ETT--TYTIVVNRG  Cbei\_4766\_Cbei\_150019575                EDDSQDSIYLDKIDLSKGNIDFL-KGKTSYDIKVDENVDSMWIKAIPESNS-----DRVRVDGSLV--------------DSSDNYK----------ETVSL-NKGK--NEIKIKVTDSK----------------------------DNQ-RTYTLNVTRG  Cbei\_4766\_Cbei\_150019575\_2              DSDNEDDIYLDNITLSEGDIDFS-KDDNSYNVDLDESVSKITIGAEPEDSD-----YSVTIDGDEV--------------RSSDDYE----------KKVSL-DKGE--NVIKVNVEDEL----------------------------NDKKRTYTLTINRG  Cbut5\_010100004691\_Cbut\_168189451       HAAKYDDPLLSDLKVSGYDVDFD-KETYSYSIKVDKDVDQITVTAKPEDSA-----YEVTVNGDDA--------------KEDDKYK----------VKVDV-KQGK--STITIRVKDENH---------------------------DLK-TTYELEVIRG  Cbut5\_010100004691\_Cbut\_168189451\_2     EDLNEDDVYLDNITLSAGKINFS-SKVTEYTVSVDDSVRYLDIKAKPMDSDE----EYVEINGEEA--------------EEDDDYE----------QTVTL-KDGK--NKITIRIENSE----------------------------KS--KKYILNVYKG  GeobDRAFT\_1055\_Gsp\_110600938            VAASNNANLSNLVLSSGTLTPTFSSTTTSYTASVANSVSSITVTPTEADTT-----ATTTVNGVAV--------------NSGTASG-----------PISL-AVGS--NTITTVVTAQD----------------------------GVTTKTYTVVVTRA  GeobDRAFT\_1055\_Gsp\_110600938\_2          AGSSNADLSNLVLSSGTLSPAFS-SATTSYTASVANSVSSITVTPTEADTT-----ATTTVNGTAV--------------NSGTASG-----------PIAL-SVGA--NTITTVVTAQD----------------------------GTTTKTYTVVVTRA  GeobDRAFT\_1055\_Gsp\_110600938\_3          AASTNANLSNLVLSSGTLSPAFA-SGTISYTASVANPVASITVTPTVEDAT-----ATVKVNGTTV--------------ASGTASA-----------PIAL-VVGS--NTITTVVTAQD----------------------------GTTTKTYTVTVTRA  LOC757233\_Spur\_115762573                --MDDADLEKLSIKPGSLNPKFH-RNTTEYNVTVASDVGKLTISCLTSDSN-----ASYQIVG-----------------GDGGRT-------------VDL-AEGKT-TTIKIDVSAED----------------------------GT-SKLYIIHAKRL  CcelDRAFT\_2267\_Ccel\_118725478           VDPTSPYLSSLTMNSGTLTPAFD-KTTLSYTATVDYGVTSIIVTPTAETPTAYGQNAVIKVNGVVT--------------NSGTGA------------SVNL-AVGV--NTITIDVTSAV----------------------------GYLTQRYTLTVTRL  CcelDRAFT\_2267\_Ccel\_118725478\_2         SNKLTSLSVLNGTTVLPIVPAFV-KNTYGYTVEVDNTVQSVMLNATCENPA-----STLTINGGAA--------------VSGQPYG-----------PITLQTTGD--TTVNVIVTTATM---------------------------AP--QTYTVTIKRK  CcelDRAFT\_2267\_Ccel\_118725478\_3         DLTLFSLSIKNGKTSLILQPNFS-STQYQYSTSVS-GTTSLSIIPTASNTNG----VNITVNGNPV--------------ASGSSYT-----------AANL-KIGA--NTINIDVISKNT---------------------------GNK-NTYICNITIS  Ot08g00500\_Otau\_116059082               TTASVSSVFPSIPPGIKLEPPFA-PTVYAYTATVPAQVAGVQVFYQLRQST-----GSVSARRLLA--------------PSLVSMR-----------YFAL-STGS--NLIRLVSNSPN----------------------------GQATDTYRFTITRE  CLOBOL\_00466\_Cbol\_160935576             DGSPNNKLSGLGVDGFNLTPSFN-RDTQEYNLIVDSSVSNITVSAYASDSN-----AR----------------------VDGAGN-------------VSL-QNGG--NDISIAVTAKN----------------------------GSV-RTYTIHVVKQ  Gura\_0755\_Gura\_148262831                PNTLSALTITGNGAAVSTTPAFA-STTTSYTASVTYDVASVVVSPTATDQN-----AVMKVNGNPV--------------PRGGNA------------TVPL-NVGS--NAVTVVVTPTI----------------------------GDP-QTYTITVTRA  Gura\_0755\_Gura\_148262831\_2              SQYLSGFTLKSGRNPVNYTPTFD-KNTTQYT-STSSGLTSITITPTTEDPA-----AVINVGGTTV--------------SSGQPV------------TLTV--SGS--TVIPITVTSNI----------------------------GSVTKKYDLTVN--  Cthe\_2506\_Cthe\_125974991                EVKSDPYLSDLVVTGAKLKPAFV-PDILNYEAVAEEDVRFVCIVAYARDDG-----AEITLNGVPV--------------KSGSISH-----------AVEL-KEGK--NELIVKVVAED----------------------------GITSRTYRISVLLE  CLOL250\_01290\_Csp\_160893736             SGNPNNYLKSLTIDNYSLTPTFAINSTKKYSLIVSENTTSVKISATPVNSH-----AS----------------------VSGTGK-------------ISL-SKGT--NTAKVTVTAQS----------------------------GAK-RTYTITIVRG  Cbei\_4763\_Cbei\_150019572                QNNDNDNIYLERLSVNNNKVQLS-KSELTYTYNVASDVKRVTIKATPEDDD-----YDVTIDNKNV--------------QSADNYK----------KEVDL-DEGT--NEFEIELEDGD----------------------------KD--RVYTLIINRG  Cbei\_4763\_Cbei\_150019572\_2              AETEHQDSIYLDKLSIDGRLFSLSQSQVNYSSNVPSDVNKVTIKAEPEKDF-----YTVKVNGDEV--------------FEDDDYK----------ATVNL-KDGE--NKIKVDVKNEN----------------------------SDEERVYTLTVIRG  Pjdr2DRAFT\_0895\_Psp\_169185627           DANLKAISLSAMGQVLRLSPAFS-PAATEYTAETEADQLDVSAAVSDSKAR-----IGLKVNGQ----------------VPASQS------------SVNL-SLGV--NVLEWTVAAEN----------------------------GNT-KTYKVTVTRV  Cbei\_1455\_Cbei\_150016335                DEDDNKDVYLKSISLSDGDLSFS-KNTSDYSVNVSGSVDEIKITAKPDCDSDEYDDYEVRIDGSTV--------------DESDKFR----------KTVSL-NKGK--NDIKITVKNDD----------------------------DK--RTYTLNIYRG  FBALC1\_04492\_Fbac\_163787771             AVHDDRTLATLGGTDLVLSPPFD-PNVSSYTAAVCNSVLSTTINATVTNSG-------DTIGGTGL---------------------------------VNL-NEGT--NMITVSVTDQC----------------------------GGT-DDYTVEVTRV  Pjdr2DRAFT\_4207\_Psp\_169189097           IVSSNANLSTLKLSDIQMNEPFK-DSTLNYTAGVAYSVLKTKVTAVPSDAN-----AKVDVLD-----------------EKGEPAG----------EFIEL-AVGT--QTFTVRVTAQD----------------------------GTTVQAYTVKITKY  Cbut5\_010100016434\_Cbut\_168191778       NNSKGGSSKLKDLILSSGDFKFD-SDVYTTKVQVEQSVNKISITPIAEDSK-----AKITINGQK---------------FTGKPI------------SVSL--AGKQTTEVDIEVESED----------------------------GESTTTYTLKIKRV  Dhaf\_0947\_Dhaf\_109647408                PSLAGLIVKDSDDHIVELNPAFN-SNTDSYKATVLLSVASVTLIPTVLDSS-----AVIKVNSMEV--------------VSGTSSA-----------PITI-NVGT--NQIEVSVTVD-----------------------------GVT-KIYTIEITKA  Dhaf\_0947\_Dhaf\_109647408\_2              PYLKAISITGNNKVAISLDQTFDPKNSFNYTALADYDDTSATVVLTADDPN-----AKLSVNGGAS--------------SSGPIT-----------FPVTMSSPGD--YSTAIVVEAAD----------------------------GTTTQSYSLKVTRP  SSA\_2023\_Ssan\_125718810                 NPELEDIRVTSSVGKVEQKGQFT-APITIQYVKHDAETVNLTVVAKNPA-------AKVSVTD-----------------AAGRVYSDL--------KNIPL-AVGA--NYLTVTSTVTDADG-------------------------QEVSLTYRLNVHRR  ANACOL\_01569\_Acol\_167770226             DATLKSLVINDQDGVDTKKISFN-PDVETYEISVPYETREVSFVPTANHEG-----ARIKIQSNRDSILPGVIPPRWYTVNSGTASKNFD-------LPYAV-GDAT--TTFTIRVEAENYDYVD-----------------------PPSYKEYIVKITRE  ANACOL\_01569\_Acol\_167770226\_2           SHDAYLKALSATNTENWQPDPFI-RTTLDYEATVKEGSSTVTITATTNHPG-----ATLTINGMAA--------------QSGVASD-----------PIDL-VDIY--TTIEIVVTAQD----------------------------GTTKMTYTIEFYNQ  ANACOL\_01569\_Acol\_167770226\_3           EKTNNADLRSLKVNYGLMTPKFK-PAVTEYEVTATEDTYSVDIIPRTDDRL-----ATYEVFAGTR--------------KIGDYNNN---------YALAL-EDGE--NEVRIVVTSPD----------------------------KTVTKEYTVMIYRN  apu\_Tthe\_114076                         NQGSNTMVVNDTVQRWRDVPIY--IYSPKDKTIVDANTSEIEIKGNTYKG------AKVTINDESFVQQ-----------ENGVFTK-----------VVPL-EYGV--NTIKIHVEPSGDKN-------------------------NELTKDITITVTRE  NEMVEDRAFT\_v1g226029\_Nvec\_156304173     --MDNADLEKLAISPGKLSPKFH-ANTTEYQVTLGSEVGQLKVDPLTSDSG-----ASYAISG-----------------SGGGKT-------------VSL-KEGEV-TTIKIEVSAED----------------------------GSTIKNYYIYANRL  Cbut5\_010100011820\_Cbut\_168190862       DDDDDDDIYLKSITLSDGEISFS-KKTTKYDVYVASTVSNLTIKAKPEDED-----YTVTIDGTEV--------------DDDDNYK----------EEVSL-SKGK--NTIEIEVEDEDE---------------------------DES-RVYTLNIYRG  Sde\_2261\_Sdeg\_90021906                  FYNKFDASLSTTSFIQPYTPTFN-SATKEYTASVNSTACSLRTRLLTNQRL-----STATINGE----------------AIGHLES----------RDFAI-VEGD--NTVEIAVTSED----------------------------GANTENYSFTFTRA  Cbut5\_010100004696\_Cbut\_168189452       FASDKSSLNLESISLSTGKIDFS-SDVTSYKVYYNKAVKNIDIKVSPEGSDKR---IRVTINDESV--------------SSDDSYK----------KNFKL-SVGE--NVFKIKIENRDDE--------------------------SQS-KTYTLKAYRG  Cbut5\_010100004696\_Cbut\_168189452\_2     DQEIEQDLYINYLTINGKEMSLS-KDKKVYDYKVDKDVKEAKIVIEPDQKY-----YTVKIGDNT---------------YEGEESIK---------KTFTL-TEGK--NEIKIKLTDGEDE--------------------------EKKQRTYTLNIYRG  DSY0534\_Dhaf\_89893280                   PSLAGLIVKDNSDHTVELNPAFN-SNTDSYQATVLLSVASVTLIPTVLDSS-----AVIKVNNTEV--------------VSGTSSA-----------PITI-NVGT--NQIEVSVTVA-----------------------------GVT-KIYTIEITKA  DSY0534\_Dhaf\_89893280\_2                 PYLKAISITGNNKGAISLAQTFDPKNSFNYTALADYDDTNATVVLTADDPN-----AKLSVNGGAS--------------SSGPIT-----------FPVTMSSLGD--YSTAIVVEAAD----------------------------GTTTQSYSLKVTRP  EUBDOL\_01948\_Edol\_160915925             KRSDNNNLASLAISSGKLTPAFH-AETTEYRVELAATVTSINITASAADAK-------AKVNG------------------TGTKA---------------L-EPGE--NKFSITVTAEN----------------------------GAV-KTYTIVAVVN  AAur\_pTC20128\_Aaur\_119952588            PLAADATLRSLAVESATLTPAFA-SDVQEYEVTTDPGATELVVTPTATGPA-----SEISVNGQTV--------------QSGSAA------------TVAV-SNG---QVVPIRVSSAD----------------------------GTALVTYSLTVKVG  Teth39\_2173\_Tpse\_167038553              NQGSNTMVVNDTVQRWRDLPIY--IYSPKDNTTVDANTNEIEIKGNTYKG------AKVTINDESFVQQ-----------ENGVFTK-----------VVPL-EYGV--NTTKIHVEPSGDKN-------------------------NELTKDITITVIRE  OSTLU\_27686\_Oluc\_145354833              QSLDDASSKGFVKLDATLSPKFA-ASTTAYDVLIAPRATHVNVMATSFAPG-----CTIAIDGV-----------------IGNMA------------N----RPAN--ATIEVTVTASG----------------------------GEK-TTYSLTTKAT  Cbei\_4761\_Cbei\_150019570                DDEDSDNVYLSSITLMGGNIDFS-KKVYTYDVQVPEDLSKITIRARPDCDSGKYDDYKVKINGVKV--------------DKDDKFK----------DDVSL-NKGK--NVIDIKVEDDD----------------------------DNE-RVYTLNITRG  SGO\_0385\_Sgor\_157151265                 NPELEDIRVTSSVGKVEQKGQFT-APITIQYVKHDAETVDLTVVAKNPA-------AKVSVTD-----------------AAGRVYSDL--------KNIPL-AVGA--NYLTVTGTVTDADG-------------------------QEVSLTYRLNVHRR  Cbei\_4762\_Cbei\_150019571                DKTDFDDIYLERLSIAGSTINLS-NSITKYTYNVDSNVNTVVIKATPENDN-----YDVTIDGEDA--------------DYDDNYK----------RTVNL-EKGQ--NIIKVEIEHNN----------------------------KD--RVYTLIINRG  Cbut5\_010100016504\_Cbut\_168191792       KNIEDKPYLKDIYVGLGYNIEFI-KDKYEYIVDVDKDIEDIYVKTRPENDN-----DIIKINGKVI--------------TKDDKFK----------KDLSL-EMGK--NKVVIEVLKNNDNNGEEYTDNDDDFSEDDVQSDKDKYESAQEKTVYTLYIYRG  Teth514\_1781\_Tsp\_167040417              NQGSNTMVVNDVVQRWRDIPIY--IYFPKDNTTVDANTNEIGIKGNTYKG------AIVTINDESFVQQ-----------ENGVFTK-----------IVPL-EYGV--NIIKIHVEPSGEKN-------------------------NELTKDITITVTKE  EUBSIR\_01285\_Esir\_167750311             WSGTDTSLKAISSDYGTLSPEFS-AKTYNYTLTVPFGTKSINFRPTALNKNF----KTVSKIGD----------------KTLSLTK-----------PTEI-KDG---DIITVTVGEGA----------------------------TA--STYKVTIKEG  EUBSIR\_01285\_Esir\_167750311\_2           VSIKNVHFTELKSEHGDMYPAFD-PSLLSYNIVIANDAEFPTVYFKVANG------CTVKIGNDA---------------ATADENG-----------VYSL-VTKS----ANTTVTVTD----------------------------GTLSESYTVKATKR  CLOL250\_01291\_Csp\_160893737             EMSDNCFLSSLQITPGELKPEFS-KDVYSYETTVPGETTSLAINALPEDSK-----SSVSIDGNENF-------------EPGKQA------------H------------VTIKITAET----------------------------G-DTHIYDLTVNVE  Cbut5\_010100013680\_Cbut\_168191230       DDEDNDDIYLDSINLSAGSIDFS-RKKAEFDVVVDKGVDSIKIQAKPEDED-----YKVEIGGEEV--------------DEDSKWR----------TSVDL-KTGK--NVVEIYIEDEDDD--------------------------DNPQRTYTLNIYRG  Cbut5\_010100013680\_Cbut\_168191230\_2     TKQDEIYVKKLVLNDGDVPVTFD-KRITSYDITVNKDLEDIIVKAEPEDND-----DLVKINGS----------------KADSNYR----------KRVALNSTGT--TTIEVKVYNDYDDDED-----------------------DYEERIYNLNIKKA  Cbei\_4696\_Cbei\_150019506                ASLNSLKISGYTLTDSDGNSGFS-SDKLDYTTTVAKEDTTEKITAIAEDDNVKS--ITASINGSDATYDL----------TSGESN------------EIPL-NTNGN-TTITIVVTAQD----------------------------GKTSKTYSIVIKNN  Cbei\_4696\_Cbei\_150019506\_2              NNSKGSNANLKNVILSQGDYTFD-PKEDITKVRVDQNVSKINVTPIAEDSK-----STISVNGEE---------------YSDSAI------------SVSL--KGAQKTEIDIEVISED----------------------------GKETKTYTLEVYRV  OSTLU\_93102\_Oluc\_145349718              TTVAMSSVFPYVPDGIAIQPAFD-PTRHTYTATVPHGVTGVQVFYQFRQSTTS---ITTTLSARRRL-------------LAASLVNM---------KYFTL-NQGD--NVIRLITTSPN----------------------------GQSSETYRLTIERL  Pjdr2DRAFT\_1346\_Psp\_169186077           DASLKRLGVYDNTNDVDKSPVLN-PDEKNYSVEVESNTKQLWMSIIPTDED-----SQLTIAGGVQ--------------DPEEPIK----------VFYGI-PDKT--TVFTIKVTSPD----------------------------GTVTETYTLTVTKK  Cbut5\_010100019699\_Cbut\_168192403       SDNSTSDVGLSSISLSSGSLTFN-KNTSSYNINVNSDLNSISVEAIPEDSD-----YTVKINDSTV--------------SESDYYS----------KKVTL-TDSV--TPVIIKVTNDE----------------------------NES-KSYTVNITKT  ACTODO\_00303\_Aodo\_154507821             DLVVDFVIDQLPYGEPGKPVDFA-PGTHTYTATGYYHAKTVSARIRAVDG------ATVTINGATPD-------------ADGRVS------------NLDL-TTGL--NVITAT-VAKD----------------------------GKE-ATYVVNITKV  AAur\_pTC20163\_Aaur\_119952571            LQGTTRGKGNTALAAVQTDAGNAVQDGTGWRLDVPAGTQTLRVATKPAADVV----SGITINGATA--------------QPGVLSD-----------PITL-SEGT--NTISIVVTAEN----------------------------GTS-QTHTLTVQRA  AAur\_pTC20163\_Aaur\_119952571\_2          LLAADATLRSLTVGSATLSPDFG-SAVQDYQITTEPGATEITVTPTSTGPS-----SEISVNGHTV--------------QNGAVT------------TVAV-TNG---QVIPISVSSAD----------------------------GTGLVTYSLTIKVQ  Daci\_5804\_Daci\_160901235                VNSTNADLSALALSSGTLAPGFG-AGTLAYTATVPNSSSTVTVTATAADAG-----ATILVNGA-----------------AASTP-------------VAL-NVGS--NTVSIEVTAQD----------------------------GTTKKTYVVTVTRE  Cbei\_4764\_Cbei\_150019573                SSDDYDDIYLDRLSVDGQSISLS-KSKVEYTYNVSSDTDEVTIKATPEDED-----YDVTIDGDSV--------------DDDDKYK----------SDVDL-KKGE--NKIKIELEDGD----------------------------DE--RVYTLIINRG  Swol\_0161\_Swol\_114565730                AKPLISLSVKAADGGPELLTCFI-SKTTGYKMTVENPLEAVKITPVPVSGAT----ASVKLNGVVQ--------------NDGIVN-------------LAV-DPEQ--NKVEVVVSES-----------------------------GKDDLTYNIDIRRL  Swol\_0161\_Swol\_114565730\_2              PLTDLSVRGKADGSGNEYLTGFS-GTSYAYNVAVENNIDAVKVVPTAVSGAT----TVIKLNGVP---------------QNGGVI-------------SNL-AIGWD-NRVDIVVIQED----------------------------RTD-RKYTIEIGRK  Cphy\_3466\_Cphy\_160881588                NYLKTLSVKDFNGNELTLTPTFDILNTKEFFLVVPNSCEGINISAATVSNK-------AVLSGTGV---------------------------------VQL-NPGL--NEITIMVIAQN----------------------------GDL-RDYKISVARE  Pjdr2DRAFT\_0826\_Psp\_169185558           ALDLALANVQLKAGDYVPSFAFD-PTNNNYTIDVPYPANVLTVTPTASYPEG----VEVNVGDSAV--------------ESGFSI------------DVPI-NDAS--SPVSITVASKQY---------------------------TDVARTYTINVNQL  fruA\_Smut\_24378602                      APLIDNITVRSDRGNVEKQGQFF-SEEPLHIQYVSNDASQVSLDIAKHNPA-----ATVTVEDK-----------------TGRVYTDP--------SHLPV-NVGA--NYFTVKSTVIDSFG-------------------------RTVTLTYRINVHRR  Sde\_2599\_Sdeg\_90022244                  FNLYDFASGSGSGTSLGLAPSFS-EAVFDYSTTVPYSSCAIAYQITAKNSG-----VSMQTNGSTSY-------------ERNVYY-------------HNL-SPGV--NYLDTTVVAED----------------------------FSTSERYTIAITRT  Sde\_2599\_Sdeg\_90022244\_2                TTDQIESNAQLLKLDTDGDYFQYVCGITDYTFFIDNNTDTINLDITPEIEG-----AKVFINNNEI--------------NPSETQ------------TLQV-NESA--GTATITVISAN----------------------------GGNSQTYTLTYIRR  ACL\_1118\_Alai\_162447972                 SDNQIIDVRFFDASTNINRLNFD-PEITTYDLEFPASTTIARLRVETHNK------ALVFINGVQ---------------EINTRK------------DFNL-TSG---NTITVKFYVIAEN--------------------------GDKGIEYTINVYKR  CLOSS21\_01234\_Csp\_167766728             NTPELKNISVSSSTGDVEKAAQFTSTEPIMIQYVKNNAETVDLNIEKKNKNAD---VQVEYDGKIY--------------NDGK--------------NIPV-KVGK--NYITVKSTVQGENG-------------------------QTATLTYRVNVHRR  amyB\_Tthe\_1168478                       NQGNNEMTINDTVYRWRDIPIF--IYSPSSNMTVDSNISTMEVKGNTYKG------AKVTINGDSFVQD-----------KNGVFTK-----------DVSL-NYGV--NKIKIHVEPND----------------------------GSVYGNDQGRITEL  COPEUT\_01026\_Ceut\_163814875             PGNQNNYLKSLKVGSAKLSPTFAINKTTTYTVNVAASVGSIKIAASPVNRY-------ATVSGT------------------G---------------TKKL-KKGK--NTFKIVCKSQS----------------------------KKA-RTYTIIINRG  CperN\_010100000445\_Cper\_168215503       RVASSNASLSDISINGETMEGFK-KDVFDYEIDLEEGTNIVPEITATSEDSN----ATIEIKN------------------------------------AEI-LPGV----SKIKVVAED----------------------------GTE-SIYTINLNVK  FER\_Atha\_155242200                      GPYTLLKNFSASQTAEALTYAFI---IKEFVVNVEGGTLNMTFTPESSPSN-----AYAFVNG-----------------IEVTSMPD----------MYSS-TDGT----LTMVGSSGSVTIDN-----------------------STA-LENVYRLNVG |

  
  
  
  
Top  


---

Family
clusters of PC-Esterase domain containing proteins with domain
architecture   
  

```
# 147; Fam55  ;N terminal Ig fold 1wlh  
219453119         SIG+ig_filamin+PC-Esterase                                                        BRAFLDRAFT_86598       677    Branchiostoma floridae                          metazoa                              hypothetical protein BRAFLDRAFT_86598 [Branchiostoma floridae].  
210090817         SIG+PC-Esterase                                                                   BRAFLDRAFT_103707      193    Branchiostoma floridae                          metazoa                              hypothetical protein BRAFLDRAFT_103707 [Branchiostoma floridae].  
219424090         SIG+ig_filamin+PC-Esterase+PC-Esterase                                            BRAFLDRAFT_72332       651    Branchiostoma floridae                          metazoa                              hypothetical protein BRAFLDRAFT_72332 [Branchiostoma floridae].  
219442081         TM+ig_filamin+PC-Esterase                                                         BRAFLDRAFT_122744      611    Branchiostoma floridae                          metazoa                              hypothetical protein BRAFLDRAFT_122744 [Branchiostoma floridae].  
219423764         SIG+ig_filamin+PC-Esterase                                                        BRAFLDRAFT_72224       604    Branchiostoma floridae                          metazoa                              hypothetical protein BRAFLDRAFT_72224 [Branchiostoma floridae].  
210096858         PC-Esterase                                                                       BRAFLDRAFT_234355      244    Branchiostoma floridae                          metazoa                              hypothetical protein BRAFLDRAFT_234355 [Branchiostoma floridae].  
210126637         SIG+ig_filamin+PC-Esterase                                                        BRAFLDRAFT_68683       585    Branchiostoma floridae                          metazoa                              hypothetical protein BRAFLDRAFT_68683 [Branchiostoma floridae].  
219490765         SIG+PC-Esterase                                                                   BRAFLDRAFT_246014      248    Branchiostoma floridae                          metazoa                              hypothetical protein BRAFLDRAFT_246014 [Branchiostoma floridae].  
210121136         SIG+PC-Esterase                                                                   BRAFLDRAFT_212157      248    Branchiostoma floridae                          metazoa                              hypothetical protein BRAFLDRAFT_212157 [Branchiostoma floridae].  
210121206         ig_filamin+PC-Esterase                                                            BRAFLDRAFT_212258      449    Branchiostoma floridae                          metazoa                              hypothetical protein BRAFLDRAFT_212258 [Branchiostoma floridae].  
219427640         ig_filamin+PC-Esterase                                                            BRAFLDRAFT_212358      452    Branchiostoma floridae                          metazoa                              hypothetical protein BRAFLDRAFT_212358 [Branchiostoma floridae].  
219428178         ig_filamin+PC-Esterase                                                            BRAFLDRAFT_212695      454    Branchiostoma floridae                          metazoa                              hypothetical protein BRAFLDRAFT_212695 [Branchiostoma floridae].  
210124180         ig_filamin+PC-Esterase                                                            BRAFLDRAFT_277198      455    Branchiostoma floridae                          metazoa                              hypothetical protein BRAFLDRAFT_277198 [Branchiostoma floridae].  
210131675         SIG+ig_filamin+PC-Esterase                                                        BRAFLDRAFT_63756       571    Branchiostoma floridae                          metazoa                              hypothetical protein BRAFLDRAFT_63756 [Branchiostoma floridae].  
210090012         ig_filamin+PC-Esterase                                                            BRAFLDRAFT_246039      455    Branchiostoma floridae                          metazoa                              hypothetical protein BRAFLDRAFT_246039 [Branchiostoma floridae].  
210115525         PC-Esterase                                                                       BRAFLDRAFT_220759      249    Branchiostoma floridae                          metazoa                              hypothetical protein BRAFLDRAFT_220759 [Branchiostoma floridae].  
219476917         ig_filamin+PC-Esterase                                                            BRAFLDRAFT_98272       564    Branchiostoma floridae                          metazoa                              hypothetical protein BRAFLDRAFT_98272 [Branchiostoma floridae].  
210121127         SIG+PC-Esterase                                                                   BRAFLDRAFT_212326      250    Branchiostoma floridae                          metazoa                              hypothetical protein BRAFLDRAFT_212326 [Branchiostoma floridae].  
210091029         PC-Esterase                                                                       BRAFLDRAFT_103574      251    Branchiostoma floridae                          metazoa                              hypothetical protein BRAFLDRAFT_103574 [Branchiostoma floridae].  
210112886         PC-Esterase                                                                       BRAFLDRAFT_225479      256    Branchiostoma floridae                          metazoa                              hypothetical protein BRAFLDRAFT_225479 [Branchiostoma floridae].  
210089856         PC-Esterase                                                                       BRAFLDRAFT_246344      257    Branchiostoma floridae                          metazoa                              hypothetical protein BRAFLDRAFT_246344 [Branchiostoma floridae].  
210121901         ig_filamin+PC-Esterase                                                            BRAFLDRAFT_211232      325    Branchiostoma floridae                          metazoa                              hypothetical protein BRAFLDRAFT_211232 [Branchiostoma floridae].  
210131957         ig_filamin+PC-Esterase                                                            BRAFLDRAFT_199192      456    Branchiostoma floridae                          metazoa                              hypothetical protein BRAFLDRAFT_199192 [Branchiostoma floridae].  
210123212         PC-Esterase                                                                       BRAFLDRAFT_72331       326    Branchiostoma floridae                          metazoa                              hypothetical protein BRAFLDRAFT_72331 [Branchiostoma floridae].  
219488955         SIG+ig_filamin+PC-Esterase                                                        BRAFLDRAFT_103710      330    Branchiostoma floridae                          metazoa                              hypothetical protein BRAFLDRAFT_103710 [Branchiostoma floridae].  
219410313         ig_filamin+PC-Esterase                                                            BRAFLDRAFT_202080      457    Branchiostoma floridae                          metazoa                              hypothetical protein BRAFLDRAFT_202080 [Branchiostoma floridae].  
219511110         ig_filamin+PC-Esterase                                                            BRAFLDRAFT_112991      340    Branchiostoma floridae                          metazoa                              hypothetical protein BRAFLDRAFT_112991 [Branchiostoma floridae].  
219421348         SIG+PC-Esterase                                                                   BRAFLDRAFT_71084       349    Branchiostoma floridae                          metazoa                              hypothetical protein BRAFLDRAFT_71084 [Branchiostoma floridae].  
210100472         ig_filamin+PC-Esterase                                                            BRAFLDRAFT_144783      354    Branchiostoma floridae                          metazoa                              hypothetical protein BRAFLDRAFT_144783 [Branchiostoma floridae].  
219455018         SIG+PC-Esterase                                                                   BRAFLDRAFT_87614       363    Branchiostoma floridae                          metazoa                              hypothetical protein BRAFLDRAFT_87614 [Branchiostoma floridae].  
219511427         ig_filamin+PC-Esterase                                                            BRAFLDRAFT_113070      381    Branchiostoma floridae                          metazoa                              hypothetical protein BRAFLDRAFT_113070 [Branchiostoma floridae].  
210126635         ig_filamin+PC-Esterase                                                            BRAFLDRAFT_68681       385    Branchiostoma floridae                          metazoa                              hypothetical protein BRAFLDRAFT_68681 [Branchiostoma floridae].  
210101009         ig_filamin+PC-Esterase                                                            BRAFLDRAFT_94105       389    Branchiostoma floridae                          metazoa                              hypothetical protein BRAFLDRAFT_94105 [Branchiostoma floridae].  
219439740         ig_filamin+PC-Esterase                                                            BRAFLDRAFT_58828       395    Branchiostoma floridae                          metazoa                              hypothetical protein BRAFLDRAFT_58828 [Branchiostoma floridae].  
219407385         ig_filamin+PC-Esterase                                                            BRAFLDRAFT_64103       399    Branchiostoma floridae                          metazoa                              hypothetical protein BRAFLDRAFT_64103 [Branchiostoma floridae].  
219438956         SIG+ig_filamin+PC-Esterase                                                        BRAFLDRAFT_280610      534    Branchiostoma floridae                          metazoa                              hypothetical protein BRAFLDRAFT_280610 [Branchiostoma floridae].  
219416835         SIG+ig_filamin+PC-Esterase                                                        BRAFLDRAFT_68682       530    Branchiostoma floridae                          metazoa                              hypothetical protein BRAFLDRAFT_68682 [Branchiostoma floridae].  
210088814         ig_filamin+PC-Esterase                                                            BRAFLDRAFT_247878      399    Branchiostoma floridae                          metazoa                              hypothetical protein BRAFLDRAFT_247878 [Branchiostoma floridae].  
210124918         SIG+ig_filamin+PC-Esterase                                                        BRAFLDRAFT_70423       525    Branchiostoma floridae                          metazoa                              hypothetical protein BRAFLDRAFT_70423 [Branchiostoma floridae].  
219408328         ig_filamin+PC-Esterase                                                            BRAFLDRAFT_200059      403    Branchiostoma floridae                          metazoa                              hypothetical protein BRAFLDRAFT_200059 [Branchiostoma floridae].  
210121807         ig_filamin+PC-Esterase                                                            BRAFLDRAFT_211092      405    Branchiostoma floridae                          metazoa                              hypothetical protein BRAFLDRAFT_211092 [Branchiostoma floridae].  
210088799         ig_filamin+PC-Esterase                                                            BRAFLDRAFT_247895      406    Branchiostoma floridae                          metazoa                              hypothetical protein BRAFLDRAFT_247895 [Branchiostoma floridae].  
210097429         TM+ig_filamin+PC-Esterase                                                         BRAFLDRAFT_97499       520    Branchiostoma floridae                          metazoa                              hypothetical protein BRAFLDRAFT_97499 [Branchiostoma floridae].  
219444204         ig_filamin+PC-Esterase                                                            BRAFLDRAFT_225371      407    Branchiostoma floridae                          metazoa                              hypothetical protein BRAFLDRAFT_225371 [Branchiostoma floridae].  
219427844         ig_filamin+PC-Esterase                                                            BRAFLDRAFT_212302      408    Branchiostoma floridae                          metazoa                              hypothetical protein BRAFLDRAFT_212302 [Branchiostoma floridae].  
219408330         ig_filamin+PC-Esterase                                                            BRAFLDRAFT_275187      518    Branchiostoma floridae                          metazoa                              hypothetical protein BRAFLDRAFT_275187 [Branchiostoma floridae].  
210105923         SIG+ig_filamin+PC-Esterase                                                        BRAFLDRAFT_89060       514    Branchiostoma floridae                          metazoa                              hypothetical protein BRAFLDRAFT_89060 [Branchiostoma floridae].  
210080656         ig_filamin+PC-Esterase                                                            BRAFLDRAFT_112650      412    Branchiostoma floridae                          metazoa                              hypothetical protein BRAFLDRAFT_112650 [Branchiostoma floridae].  
219472805         SIG+ig_filamin+PC-Esterase                                                        BRAFLDRAFT_267691      415    Branchiostoma floridae                          metazoa                              hypothetical protein BRAFLDRAFT_267691 [Branchiostoma floridae].  
219427654         ig_filamin+PC-Esterase                                                            BRAFLDRAFT_212311      427    Branchiostoma floridae                          metazoa                              hypothetical protein BRAFLDRAFT_212311 [Branchiostoma floridae].  
210124919         ig_filamin+PC-Esterase                                                            BRAFLDRAFT_70425       457    Branchiostoma floridae                          metazoa                              hypothetical protein BRAFLDRAFT_70425 [Branchiostoma floridae].  
219409155         ig_filamin+PC-Esterase                                                            BRAFLDRAFT_200335      428    Branchiostoma floridae                          metazoa                              hypothetical protein BRAFLDRAFT_200335 [Branchiostoma floridae].  
210105885         SIG+ig_filamin+PC-Esterase                                                        BRAFLDRAFT_89054       504    Branchiostoma floridae                          metazoa                              hypothetical protein BRAFLDRAFT_89054 [Branchiostoma floridae].  
219409115         ig_filamin+PC-Esterase                                                            BRAFLDRAFT_200255      428    Branchiostoma floridae                          metazoa                              hypothetical protein BRAFLDRAFT_200255 [Branchiostoma floridae].  
210084583         ig_filamin+PC-Esterase                                                            BRAFLDRAFT_254658      430    Branchiostoma floridae                          metazoa                              hypothetical protein BRAFLDRAFT_254658 [Branchiostoma floridae].  
219505557         ig_filamin+PC-Esterase                                                            BRAFLDRAFT_256938      436    Branchiostoma floridae                          metazoa                              hypothetical protein BRAFLDRAFT_256938 [Branchiostoma floridae].  
210121117         ig_filamin+PC-Esterase                                                            BRAFLDRAFT_212299      438    Branchiostoma floridae                          metazoa                              hypothetical protein BRAFLDRAFT_212299 [Branchiostoma floridae].  
210091362         ig_filamin+PC-Esterase                                                            BRAFLDRAFT_243661      438    Branchiostoma floridae                          metazoa                              hypothetical protein BRAFLDRAFT_243661 [Branchiostoma floridae].  
210105893         ig_filamin+PC-Esterase                                                            BRAFLDRAFT_89067       492    Branchiostoma floridae                          metazoa                              hypothetical protein BRAFLDRAFT_89067 [Branchiostoma floridae].  
219487806         SIG+ig_filamin+PC-Esterase                                                        BRAFLDRAFT_103188      441    Branchiostoma floridae                          metazoa                              hypothetical protein BRAFLDRAFT_103188 [Branchiostoma floridae].  
219492117         ig_filamin+PC-Esterase                                                            BRAFLDRAFT_247115      444    Branchiostoma floridae                          metazoa                              hypothetical protein BRAFLDRAFT_247115 [Branchiostoma floridae].  
219488951         ig_filamin+PC-Esterase                                                            BRAFLDRAFT_244532      485    Branchiostoma floridae                          metazoa                              hypothetical protein BRAFLDRAFT_244532 [Branchiostoma floridae].  
210097423         ig_filamin+PC-Esterase                                                            BRAFLDRAFT_233553      457    Branchiostoma floridae                          metazoa                              hypothetical protein BRAFLDRAFT_233553 [Branchiostoma floridae].  
210123210         SIG+ig_filamin+PC-Esterase                                                        BRAFLDRAFT_72328       483    Branchiostoma floridae                          metazoa                              hypothetical protein BRAFLDRAFT_72328 [Branchiostoma floridae].  
210086420         ig_filamin+PC-Esterase                                                            BRAFLDRAFT_251829      482    Branchiostoma floridae                          metazoa                              hypothetical protein BRAFLDRAFT_251829 [Branchiostoma floridae].  
210095360         ig_filamin+PC-Esterase                                                            BRAFLDRAFT_237164      444    Branchiostoma floridae                          metazoa                              hypothetical protein BRAFLDRAFT_237164 [Branchiostoma floridae].  
219407681         SIG+ig_filamin+PC-Esterase                                                        BRAFLDRAFT_199653      476    Branchiostoma floridae                          metazoa                              hypothetical protein BRAFLDRAFT_199653 [Branchiostoma floridae].  
210081309         ig_filamin+PC-Esterase                                                            BRAFLDRAFT_112279      475    Branchiostoma floridae                          metazoa                              hypothetical protein BRAFLDRAFT_112279 [Branchiostoma floridae].  
210085052         ig_filamin+PC-Esterase                                                            BRAFLDRAFT_253963      475    Branchiostoma floridae                          metazoa                              hypothetical protein BRAFLDRAFT_253963 [Branchiostoma floridae].  
219408326         ig_filamin+PC-Esterase                                                            BRAFLDRAFT_200118      474    Branchiostoma floridae                          metazoa                              hypothetical protein BRAFLDRAFT_200118 [Branchiostoma floridae].  
210090583         ig_filamin+PC-Esterase                                                            BRAFLDRAFT_245008      472    Branchiostoma floridae                          metazoa                              hypothetical protein BRAFLDRAFT_245008 [Branchiostoma floridae].  
210125281         SIG+PC-Esterase                                                                   BRAFLDRAFT_119289      469    Branchiostoma floridae                          metazoa                              hypothetical protein BRAFLDRAFT_119289 [Branchiostoma floridae].  
219467637         ig_filamin+PC-Esterase                                                            BRAFLDRAFT_227654      469    Branchiostoma floridae                          metazoa                              hypothetical protein BRAFLDRAFT_227654 [Branchiostoma floridae].  
219499632         ig_filamin+PC-Esterase                                                            BRAFLDRAFT_252623      467    Branchiostoma floridae                          metazoa                              hypothetical protein BRAFLDRAFT_252623 [Branchiostoma floridae].  
219426209         ig_filamin+PC-Esterase                                                            BRAFLDRAFT_211130      448    Branchiostoma floridae                          metazoa                              hypothetical protein BRAFLDRAFT_211130 [Branchiostoma floridae].  
219457742         ig_filamin+PC-Esterase                                                            BRAFLDRAFT_125414      465    Branchiostoma floridae                          metazoa                              hypothetical protein BRAFLDRAFT_125414 [Branchiostoma floridae].  
219476361         ig_filamin+PC-Esterase                                                            BRAFLDRAFT_234339      465    Branchiostoma floridae                          metazoa                              hypothetical protein BRAFLDRAFT_234339 [Branchiostoma floridae].  
210088800         ig_filamin+PC-Esterase                                                            BRAFLDRAFT_247888      464    Branchiostoma floridae                          metazoa                              hypothetical protein BRAFLDRAFT_247888 [Branchiostoma floridae].  
210117398         ig_filamin+PC-Esterase                                                            BRAFLDRAFT_279896      463    Branchiostoma floridae                          metazoa                              hypothetical protein BRAFLDRAFT_279896 [Branchiostoma floridae].  
219435150         ig_filamin+PC-Esterase                                                            BRAFLDRAFT_217744      463    Branchiostoma floridae                          metazoa                              hypothetical protein BRAFLDRAFT_217744 [Branchiostoma floridae].  
210096889         ig_filamin+PC-Esterase                                                            BRAFLDRAFT_234356      462    Branchiostoma floridae                          metazoa                              hypothetical protein BRAFLDRAFT_234356 [Branchiostoma floridae].  
210102381         ig_filamin+PC-Esterase                                                            BRAFLDRAFT_92735       462    Branchiostoma floridae                          metazoa                              hypothetical protein BRAFLDRAFT_92735 [Branchiostoma floridae].  
219444568         TM+ig_filamin+PC-Esterase                                                         BRAFLDRAFT_82210       462    Branchiostoma floridae                          metazoa                              hypothetical protein BRAFLDRAFT_82210 [Branchiostoma floridae].  
219406467         ig_filamin+PC-Esterase                                                            BRAFLDRAFT_198994      460    Branchiostoma floridae                          metazoa                              hypothetical protein BRAFLDRAFT_198994 [Branchiostoma floridae].  
219490744         ig_filamin+PC-Esterase                                                            BRAFLDRAFT_246052      460    Branchiostoma floridae                          metazoa                              hypothetical protein BRAFLDRAFT_246052 [Branchiostoma floridae].  
210090819         ig_filamin+PC-Esterase                                                            BRAFLDRAFT_244546      458    Branchiostoma floridae                          metazoa                              hypothetical protein BRAFLDRAFT_244546 [Branchiostoma floridae].  
219470181         ig_filamin+PC-Esterase                                                            BRAFLDRAFT_229577      458    Branchiostoma floridae                          metazoa                              hypothetical protein BRAFLDRAFT_229577 [Branchiostoma floridae].  
210130782         ig_filamin+PC-Esterase                                                            BRAFLDRAFT_200170      448    Branchiostoma floridae                          metazoa                              hypothetical protein BRAFLDRAFT_200170 [Branchiostoma floridae].  
219457734         SIG+ig_filamin+PC-Esterase                                                        BRAFLDRAFT_89061       1170   Branchiostoma floridae                          metazoa                              hypothetical protein BRAFLDRAFT_89061 [Branchiostoma floridae].  
219453624         SIG+ig_filamin+PC-Esterase                                                        BRAFLDRAFT_86869       573    Branchiostoma floridae                          metazoa                              hypothetical protein BRAFLDRAFT_86869 [Branchiostoma floridae].  
219424086         SIG+ig_filamin+PC-Esterase                                                        BRAFLDRAFT_72330       702    Branchiostoma floridae                          metazoa                              hypothetical protein BRAFLDRAFT_72330 [Branchiostoma floridae].  
219509902         PC-Esterase                                                                       BRAFLDRAFT_112651      166    Branchiostoma floridae                          metazoa                              hypothetical protein BRAFLDRAFT_112651 [Branchiostoma floridae].  
219457744         ig_filamin+PC-Esterase+PDZ                                                        BRAFLDRAFT_89069       1091   Branchiostoma floridae                          metazoa                              hypothetical protein BRAFLDRAFT_89069 [Branchiostoma floridae].  
198420517         SIG+ig_filamin+PC-Esterase                                                        LOC100179173           508    Ciona intestinalis                              metazoa                              PREDICTED: hypothetical protein [Ciona intestinalis].  
198436216         ig_filamin+PC-Esterase                                                            LOC100176809           558    Ciona intestinalis                              metazoa                              PREDICTED: similar to LOC495510 protein [Ciona intestinalis].  
198423167         SIG+ig_filamin+PC-Esterase                                                        LOC100179546           559    Ciona intestinalis                              metazoa                              PREDICTED: similar to Family with sequence similarity 55, member C  
198423237         SIG+ig_filamin+PC-Esterase                                                        LOC100187384           570    Ciona intestinalis                              metazoa                              PREDICTED: similar to Family with sequence similarity 55, member C  
198428269         SIG+ig_filamin+PC-Esterase                                                        LOC100181371           577    Ciona intestinalis                              metazoa                              PREDICTED: similar to Family with sequence similarity 55, member C  
198432323         SIG+ig_filamin+PC-Esterase                                                        LOC100182867           578    Ciona intestinalis                              metazoa                              PREDICTED: hypothetical protein [Ciona intestinalis].  
198421924         SIG+ig_filamin+PC-Esterase                                                        LOC100186962           582    Ciona intestinalis                              metazoa                              PREDICTED: similar to Family with sequence similarity 55, member C  
198421995         SIG+ig_filamin+PC-Esterase                                                        LOC100186126           583    Ciona intestinalis                              metazoa                              PREDICTED: hypothetical protein [Ciona intestinalis].  
198433436         ig_filamin+PC-Esterase                                                            LOC100183201           483    Ciona intestinalis                              metazoa                              PREDICTED: hypothetical protein [Ciona intestinalis].  
115813883         PC-Esterase                                                                       LOC583557              300    Strongylocentrotus purpuratus                   metazoa>echinodermata                PREDICTED: hypothetical protein [Strongylocentrotus purpuratus].  
115708977         ig_filamin+PC-Esterase                                                            LOC576074              486    Strongylocentrotus purpuratus                   metazoa>echinodermata                PREDICTED: hypothetical protein, partial [Strongylocentrotus  
115622005         ig_filamin+PC-Esterase                                                            LOC766048              486    Strongylocentrotus purpuratus                   metazoa>echinodermata                PREDICTED: hypothetical protein, partial [Strongylocentrotus  
115940606         SIG+ig_filamin+PC-Esterase                                                        LOC760795              596    Strongylocentrotus purpuratus                   metazoa>echinodermata                PREDICTED: hypothetical protein [Strongylocentrotus purpuratus].  
115896601         ig_filamin+PC-Esterase                                                            LOC585130              396    Strongylocentrotus purpuratus                   metazoa>echinodermata                PREDICTED: hypothetical protein, partial [Strongylocentrotus  
115924528         PC-Esterase                                                                       LOC757496              250    Strongylocentrotus purpuratus                   metazoa>echinodermata                PREDICTED: hypothetical protein [Strongylocentrotus purpuratus].  
115746524         ig_filamin+PC-Esterase                                                            LOC762511              434    Strongylocentrotus purpuratus                   metazoa>echinodermata                PREDICTED: hypothetical protein [Strongylocentrotus purpuratus].  
72112723          PC-Esterase                                                                       LOC577401              296    Strongylocentrotus purpuratus                   metazoa>echinodermata                PREDICTED: hypothetical protein isoform 1 [Strongylocentrotus  
115638839         ig_filamin+PC-Esterase                                                            LOC593965              500    Strongylocentrotus purpuratus                   metazoa>echinodermata                PREDICTED: hypothetical protein [Strongylocentrotus purpuratus].  
115774648         SIG+ig_filamin+PC-Esterase                                                        LOC590594              504    Strongylocentrotus purpuratus                   metazoa>echinodermata                PREDICTED: hypothetical protein [Strongylocentrotus purpuratus].  
115973317         ig_filamin+PC-Esterase                                                            LOC759291              521    Strongylocentrotus purpuratus                   metazoa>echinodermata                PREDICTED: hypothetical protein [Strongylocentrotus purpuratus].  
115746520         ig_filamin+PC-Esterase                                                            LOC589853              520    Strongylocentrotus purpuratus                   metazoa>echinodermata                PREDICTED: hypothetical protein [Strongylocentrotus purpuratus].  
115717927         ig_filamin+PC-Esterase                                                            LOC577194              519    Strongylocentrotus purpuratus                   metazoa>echinodermata                PREDICTED: hypothetical protein [Strongylocentrotus purpuratus].  
109495359         ig_filamin+PC-Esterase                                                            LOC680711              512    Rattus norvegicus                               metazoa>vertebrata                   PREDICTED: hypothetical protein [Rattus norvegicus].  
109484637         ig_filamin+PC-Esterase                                                            RGD1566243_predicted   511    Rattus norvegicus                               metazoa>vertebrata                   PREDICTED: hypothetical protein isoform 1 [Rattus norvegicus].  
109496770         ig_filamin+PC-Esterase                                                            RGD1562319_predicted   519    Rattus norvegicus                               metazoa>vertebrata                   PREDICTED: hypothetical protein [Rattus norvegicus].  
109484635         ig_filamin+PC-Esterase                                                            RGD1566243_predicted   510    Rattus norvegicus                               metazoa>vertebrata                   PREDICTED: hypothetical protein isoform 2 [Rattus norvegicus].  
109484633         ig_filamin+PC-Esterase                                                            RGD1566243_predicted   509    Rattus norvegicus                               metazoa>vertebrata                   PREDICTED: hypothetical protein isoform 3 [Rattus norvegicus].  
118083606         SIG+ig_filamin+PC-Esterase                                                        LOC418403              561    Gallus gallus                                   metazoa>vertebrata                   PREDICTED: hypothetical protein [Gallus gallus].  
149259926         SIG+ig_filamin+PC-Esterase                                                        EG640268               583    Mus musculus                                    metazoa>vertebrata                   PREDICTED: similar to protein of unknown function [Mus musculus].  
109495373         ig_filamin+PC-Esterase                                                            RGD1559954_predicted   527    Rattus norvegicus                               metazoa>vertebrata                   PREDICTED: hypothetical protein [Rattus norvegicus].  
109493073         SIG+ig_filamin+PC-Esterase                                                        LOC681096              559    Rattus norvegicus                               metazoa>vertebrata                   PREDICTED: hypothetical protein [Rattus norvegicus].  
149259611         SIG+ig_filamin+PC-Esterase                                                        EG640268               583    Mus musculus                                    metazoa>vertebrata                   PREDICTED: similar to protein of unknown function [Mus musculus].  
68163537          SIG+ig_filamin+PC-Esterase                                                        Fam55b                 542    Rattus norvegicus                               metazoa>vertebrata                   hypothetical protein LOC500991 [Rattus norvegicus].  
70778978          SIG+ig_filamin+PC-Esterase                                                        Fam55d                 543    Mus musculus                                    metazoa>vertebrata                   hypothetical protein LOC244853 [Mus musculus].  
117647230         SIG+ig_filamin+PC-Esterase                                                        FAM55D                 544    Homo sapiens                                    metazoa>vertebrata                   hypothetical protein LOC54827 isoform 1 [Homo sapiens].  
114640408         SIG+ig_filamin+PC-Esterase                                                        LOC740659              544    Pan troglodytes                                 metazoa>vertebrata                   PREDICTED: similar to brush border [Pan troglodytes].  
114640447         SIG+ig_filamin+PC-Esterase                                                        LOC741077              546    Pan troglodytes                                 metazoa>vertebrata                   PREDICTED: similar to protein of unknown function [Pan  
114588296         SIG+ig_filamin+PC-Esterase                                                        LOC740620              559    Pan troglodytes                                 metazoa>vertebrata                   PREDICTED: similar to Family with sequence similarity 55, member C  
110625603         ig_filamin+PC-Esterase                                                            BC055004               555    Mus musculus                                    metazoa>vertebrata                   hypothetical protein LOC381680 [Mus musculus].  
109484655         SIG+ig_filamin+PC-Esterase                                                        LOC684289              556    Rattus norvegicus                               metazoa>vertebrata                   PREDICTED: similar to Protein FAM55D precursor [Rattus norvegicus].  
21313426          SIG+ig_filamin+PC-Esterase                                                        4432416J03Rik          558    Mus musculus                                    metazoa>vertebrata                   hypothetical protein LOC78252 [Mus musculus].  
82965518          SIG+ig_filamin+PC-Esterase                                                        Gm1752                 559    Mus musculus                                    metazoa>vertebrata                   PREDICTED: hypothetical protein LOC385658 isoform 5 [Mus musculus].  
21450781          SIG+ig_filamin+PC-Esterase                                                        FAM55C                 559    Homo sapiens                                    metazoa>vertebrata                   hypothetical protein LOC91775 [Homo sapiens].  
169202030         SIG+ig_filamin+PC-Esterase                                                        FAM55B                 559    Homo sapiens                                    metazoa>vertebrata                   PREDICTED: hypothetical protein LOC120406 [Homo sapiens].  
109495375         ig_filamin+PC-Esterase                                                            RGD1562319_predicted   655    Rattus norvegicus                               metazoa>vertebrata                   PREDICTED: hypothetical protein [Rattus norvegicus].  
22748691          ig_filamin+PC-Esterase                                                            FAM55A                 405    Homo sapiens                                    metazoa>vertebrata                   hypothetical protein LOC120400 [Homo sapiens].  
114640410         SIG+ig_filamin+PC-Esterase                                                        LOC740719              559    Pan troglodytes                                 metazoa>vertebrata                   PREDICTED: hypothetical protein [Pan troglodytes].  
189528946         ig_filamin+PC-Esterase                                                            LOC100007345           361    Danio rerio                                     metazoa>vertebrata>actinopterygii    PREDICTED: similar to mCG130772 [Danio rerio].  
189528944         SIG+ig_filamin+PC-Esterase                                                        LOC100007333           536    Danio rerio                                     metazoa>vertebrata>actinopterygii    PREDICTED: hypothetical protein [Danio rerio].  
47213943          SIG+ig_filamin+PC-Esterase                                                        GSTEN:00010567:G:001   525    Tetraodon nigroviridis                          metazoa>vertebrata>actinopterygii    unnamed protein product [Tetraodon nigroviridis].  
47226931          ig_filamin+PC-Esterase                                                            GSTEN:00025975:G:001   500    Tetraodon nigroviridis                          metazoa>vertebrata>actinopterygii    unnamed protein product [Tetraodon nigroviridis].  
116517256         SIG+ig_filamin+PC-Esterase                                                        zgc:153086             566    Danio rerio                                     metazoa>vertebrata>actinopterygii    hypothetical protein LOC564277 [Danio rerio].  
189536535         ig_filamin+PC-Esterase                                                            LOC100006583           493    Danio rerio                                     metazoa>vertebrata>actinopterygii    PREDICTED: hypothetical protein [Danio rerio].  
189536529         ig_filamin+PC-Esterase                                                            LOC570927              492    Danio rerio                                     metazoa>vertebrata>actinopterygii    PREDICTED: hypothetical protein [Danio rerio].  
189536539         ig_filamin+PC-Esterase                                                            LOC100006634           492    Danio rerio                                     metazoa>vertebrata>actinopterygii    PREDICTED: similar to mCG56186 [Danio rerio].  
125836783         ig_filamin+PC-Esterase                                                            LOC566330              481    Danio rerio                                     metazoa>vertebrata>actinopterygii    PREDICTED: hypothetical protein [Danio rerio].  
189528942         ig_filamin+PC-Esterase                                                            LOC100007318           465    Danio rerio                                     metazoa>vertebrata>actinopterygii    PREDICTED: hypothetical protein [Danio rerio].  
219450403         DISCOIDIN+PC-Esterase                                                             BRAFLDRAFT_85160       415    Branchiostoma floridae                          metazoa                              hypothetical protein BRAFLDRAFT_85160 [Branchiostoma floridae].  
# 10;  
15230646          SIG+ig_filamin+PC-Esterase                                                        AT3G06150              594    Arabidopsis thaliana                            viridiplantae                        unknown protein [Arabidopsis thaliana].  
162664180         TM+ig_filamin+PC-Esterase                                                         PHYPADRAFT_99781       593    Physcomitrella patens subsp. patens             viridiplantae                        predicted protein [Physcomitrella patens subsp. patens].  
15239640          SIG+ig_filamin+PC-Esterase                                                        AT5G19060              551    Arabidopsis thaliana                            viridiplantae                        unknown protein [Arabidopsis thaliana].  
Smoe1000010461    SIG+ig_filamin+PC-Esterase                                                        Smoe1000010461         528    Selaginella moellendorffii                      viridiplantae                        fgenesh2_pg.C_scaffold_11000100  
Smoe1000011336    SIG+ig_filamin+PC-Esterase                                                        Smoe1000011336         525    Selaginella moellendorffii                      viridiplantae                        e_gw1.10.792.1  
Smoe1000017658    ig_filamin+PC-Esterase                                                            Smoe1000017658         509    Selaginella moellendorffii                      viridiplantae                        e_gw1.23.692.1  
Smoe1000009250    ig_filamin+PC-Esterase                                                            Smoe1000009250         494    Selaginella moellendorffii                      viridiplantae                        e_gw1.55.332.1  
162693858         ig_filamin+PC-Esterase                                                            PHYPADRAFT_116042      468    Physcomitrella patens subsp. patens             viridiplantae                        predicted protein [Physcomitrella patens subsp. patens].  
Smoe1000017654    ig_filamin+PC-Esterase                                                            Smoe1000017654         437    Selaginella moellendorffii                      viridiplantae                        gw1.23.674.1  
Smoe1000017655    SIG+ig_filamin+PC-Esterase                                                        Smoe1000017655         432    Selaginella moellendorffii                      viridiplantae                        estExt_fgenesh2_pg.C_230135  
# 12;  
Lgig1000015087    ig_filamin+PC-Esterase                                                            Lgig1000015087         741    Lottia gigantea                                 metazoa>mollusca                     fgenesh2_pg.C_sca_143000002  
Lgig1000015088    ig_filamin+PC-Esterase                                                            Lgig1000015088         718    Lottia gigantea                                 metazoa>mollusca                     fgenesh2_pg.C_sca_143000005  
Lgig1000015005    TM+ig_filamin+PC-Esterase                                                         Lgig1000015005         641    Lottia gigantea                                 metazoa>mollusca                     fgenesh2_pg.C_sca_135000025  
Lgig1000012113    SIG+ig_filamin+PC-Esterase                                                        Lgig1000012113         616    Lottia gigantea                                 metazoa>mollusca                     fgenesh2_pg.C_sca_37000095  
Lgig1000015291    SIG+ig_filamin+PC-Esterase                                                        Lgig1000015291         614    Lottia gigantea                                 metazoa>mollusca                     fgenesh2_pg.C_sca_161000013  
Lgig1000015294    SIG+ig_filamin+PC-Esterase                                                        Lgig1000015294         614    Lottia gigantea                                 metazoa>mollusca                     fgenesh2_pg.C_sca_161000016  
Lgig1000015404    SIG+ig_filamin+PC-Esterase                                                        Lgig1000015404         613    Lottia gigantea                                 metazoa>mollusca                     fgenesh2_pg.C_sca_171000003  
Lgig1000012131    SIG+ig_filamin+PC-Esterase                                                        Lgig1000012131         605    Lottia gigantea                                 metazoa>mollusca                     fgenesh2_pg.C_sca_37000164  
Lgig1000013481    SIG+ig_filamin+PC-Esterase                                                        Lgig1000013481         602    Lottia gigantea                                 metazoa>mollusca                     fgenesh2_pg.C_sca_65000070  
Lgig1000023552    SIG+ig_filamin+PC-Esterase                                                        Lgig1000023552         598    Lottia gigantea                                 metazoa>mollusca                     estExt_fgenesh2_pg.C_sca_1720017  
Lgig1000009656    ig_filamin+PC-Esterase                                                            Lgig1000009656         589    Lottia gigantea                                 metazoa>mollusca                     fgenesh2_pg.C_sca_9000026  
Lgig1000013315    SIG+ig_filamin+PC-Esterase                                                        Lgig1000013315         579    Lottia gigantea                                 metazoa>mollusca                     fgenesh2_pg.C_sca_60000104  
Lgig1000015293    SIG+PC-Esterase                                                                   Lgig1000015293         297    Lottia gigantea                                 metazoa>mollusca                     fgenesh2_pg.C_sca_161000015  
Lgig1000012585    PC-Esterase                                                                       Lgig1000012585         293    Lottia gigantea                                 metazoa>mollusca                     fgenesh2_pg.C_sca_44000141  
Lgig1000016786    PC-Esterase                                                                       Lgig1000016786         282    Lottia gigantea                                 metazoa>mollusca                     fgenesh2_pg.C_sca_2352000001  
#  
Pbla1000008427    SIG+PC-Esterase                                                                   Pbla1000008427         511    Phycomyces blakesleeanus                        fungi>basal fungal lineages          fgeneshPB_pg.20__165  
Pbla1000012657    SIG+PC-Esterase                                                                   Pbla1000012657         509    Phycomyces blakesleeanus                        fungi>basal fungal lineages          estExt_fgeneshPB_pg.C_30509  
Pbla1000009791    SIG+PC-Esterase                                                                   Pbla1000009791         701    Phycomyces blakesleeanus                        fungi>basal fungal lineages          fgeneshPB_pg.34__126  
  
-  
####################################  
# 83;  PMR5  
42567700          SIG+PC-Esterase                                                                   AT5G06700              608    Arabidopsis thaliana                            viridiplantae                        unknown protein [Arabidopsis thaliana].  
15229945          SIG+PC-Esterase                                                                   AT3G12060              556    Arabidopsis thaliana                            viridiplantae                        unknown protein [Arabidopsis thaliana].  
18420285          SIG+PC-Esterase                                                                   AT5G20680              551    Arabidopsis thaliana                            viridiplantae                        unknown protein [Arabidopsis thaliana].  
22330347          SIG+PC-Esterase                                                                   AT1G60790              541    Arabidopsis thaliana                            viridiplantae                        unknown protein [Arabidopsis thaliana].  
22328922          SIG+PC-Esterase                                                                   AT4G25360              533    Arabidopsis thaliana                            viridiplantae                        unknown protein [Arabidopsis thaliana].  
15237211          SIG+PC-Esterase                                                                   AT5G15890              526    Arabidopsis thaliana                            viridiplantae                        unknown protein [Arabidopsis thaliana].  
15242138          SIG+PC-Esterase                                                                   YLS7                   501    Arabidopsis thaliana                            viridiplantae                        YLS7 (yellow-leaf-specific gene 7) [Arabidopsis thaliana].  
15228789          SIG+PC-Esterase                                                                   AT3G55990              487    Arabidopsis thaliana                            viridiplantae                        unknown protein [Arabidopsis thaliana].  
15242021          SIG+PC-Esterase                                                                   AT5G20590              485    Arabidopsis thaliana                            viridiplantae                        unknown protein [Arabidopsis thaliana].  
15224349          TM+PC-Esterase                                                                    AT2G37720              482    Arabidopsis thaliana                            viridiplantae                        unknown protein [Arabidopsis thaliana].  
15228743          SIG+PC-Esterase                                                                   AT3G62390              475    Arabidopsis thaliana                            viridiplantae                        unknown protein [Arabidopsis thaliana].  
42572279          TM+PC-Esterase                                                                    AT3G06080              469    Arabidopsis thaliana                            viridiplantae                        unknown protein [Arabidopsis thaliana].  
15239660          TM+PC-Esterase                                                                    AT5G19160              464    Arabidopsis thaliana                            viridiplantae                        unknown protein [Arabidopsis thaliana].  
15239856          SIG+PC-Esterase                                                                   AT5G49340              457    Arabidopsis thaliana                            viridiplantae                        unknown protein [Arabidopsis thaliana].  
30678135          PC-Esterase                                                                       AT1G01430              456    Arabidopsis thaliana                            viridiplantae                        unknown protein [Arabidopsis thaliana].  
15228429          SIG+PC-Esterase                                                                   AT3G11030              451    Arabidopsis thaliana                            viridiplantae                        unknown protein [Arabidopsis thaliana].  
18413698          SIG+PC-Esterase                                                                   AT5G01620              449    Arabidopsis thaliana                            viridiplantae                        unknown protein [Arabidopsis thaliana].  
79361624          SIG+TM+PC-Esterase                                                                AT1G48880              445    Arabidopsis thaliana                            viridiplantae                        unknown protein [Arabidopsis thaliana].  
15234058          TM+PC-Esterase                                                                    AT4G01080              442    Arabidopsis thaliana                            viridiplantae                        unknown protein [Arabidopsis thaliana].  
Smoe1000012431    SIG+PC-Esterase                                                                   Smoe1000012431         441    Selaginella moellendorffii                      viridiplantae                        fgenesh2_pg.C_scaffold_2000456  
18412850          SIG+PC-Esterase                                                                   AT5G01360              434    Arabidopsis thaliana                            viridiplantae                        unknown protein [Arabidopsis thaliana].  
15237044          SIG+PC-Esterase                                                                   AT4G11090              432    Arabidopsis thaliana                            viridiplantae                        unknown protein [Arabidopsis thaliana].  
30686312          SIG+PC-Esterase                                                                   AT4G23790              430    Arabidopsis thaliana                            viridiplantae                        unknown protein [Arabidopsis thaliana].  
15229797          SIG+PC-Esterase                                                                   AT3G11570              427    Arabidopsis thaliana                            viridiplantae                        unknown protein [Arabidopsis thaliana].  
18405285          SIG+PC-Esterase                                                                   AT2G40160              427    Arabidopsis thaliana                            viridiplantae                        unknown protein [Arabidopsis thaliana].  
15237213          SIG+PC-Esterase                                                                   AT5G15900              426    Arabidopsis thaliana                            viridiplantae                        unknown protein [Arabidopsis thaliana].  
42569796          SIG+PC-Esterase                                                                   AT2G40320              425    Arabidopsis thaliana                            viridiplantae                        unknown protein [Arabidopsis thaliana].  
18405283          SIG+PC-Esterase                                                                   AT2G40150              424    Arabidopsis thaliana                            viridiplantae                        unknown protein [Arabidopsis thaliana].  
Smoe1000004762    SIG+PC-Esterase                                                                   Smoe1000004762         425    Selaginella moellendorffii                      viridiplantae                        fgenesh2_pg.C_scaffold_40000042  
15223079          SIG+PC-Esterase                                                                   AT1G70230              416    Arabidopsis thaliana                            viridiplantae                        unknown protein [Arabidopsis thaliana].  
15232914          SIG+PC-Esterase                                                                   AT3G28150              414    Arabidopsis thaliana                            viridiplantae                        unknown protein [Arabidopsis thaliana].  
145337478         SIG+PC-Esterase                                                                   AT1G73140              413    Arabidopsis thaliana                            viridiplantae                        unknown protein [Arabidopsis thaliana].  
42567686          SIG+PC-Esterase                                                                   AT5G06230              413    Arabidopsis thaliana                            viridiplantae                        unknown protein [Arabidopsis thaliana].  
15225951          SIG+PC-Esterase                                                                   AT2G14530              412    Arabidopsis thaliana                            viridiplantae                        unknown protein [Arabidopsis thaliana].  
18404712          SIG+PC-Esterase                                                                   AT2G38320              410    Arabidopsis thaliana                            viridiplantae                        unknown protein [Arabidopsis thaliana].  
30697938          SIG+PC-Esterase                                                                   AT5G64020              408    Arabidopsis thaliana                            viridiplantae                        unknown protein [Arabidopsis thaliana].  
15237710          SIG+PC-Esterase                                                                   AT5G64470              407    Arabidopsis thaliana                            viridiplantae                        unknown protein [Arabidopsis thaliana].  
168058401         SIG+PC-Esterase                                                                   PHYPADRAFT_149451      406    Physcomitrella patens subsp. patens             viridiplantae                        predicted protein [Physcomitrella patens subsp. patens].  
15237711          SIG+PC-Esterase                                                                   PMR5                   402    Arabidopsis thaliana                            viridiplantae                        PMR5 (POWDERY MILDEW RESISTANT 5) [Arabidopsis thaliana].  
Smoe1000015181    SIG+PC-Esterase                                                                   Smoe1000015181         402    Selaginella moellendorffii                      viridiplantae                        e_gw1.3.1277.1  
18402220          SIG+PC-Esterase                                                                   AT2G30010              398    Arabidopsis thaliana                            viridiplantae                        unknown protein [Arabidopsis thaliana].  
168031529         PC-Esterase                                                                       PHYPADRAFT_187151      387    Physcomitrella patens subsp. patens             viridiplantae                        predicted protein [Physcomitrella patens subsp. patens].  
18403435          SIG+PC-Esterase                                                                   AT2G34070              385    Arabidopsis thaliana                            viridiplantae                        unknown protein [Arabidopsis thaliana].  
18396930          SIG+PC-Esterase                                                                   AT1G29050              380    Arabidopsis thaliana                            viridiplantae                        unknown protein [Arabidopsis thaliana].  
18410028          SIG+PC-Esterase                                                                   AT3G54260              379    Arabidopsis thaliana                            viridiplantae                        unknown protein [Arabidopsis thaliana].  
167997023         PC-Esterase                                                                       PHYPADRAFT_173777      378    Physcomitrella patens subsp. patens             viridiplantae                        predicted protein [Physcomitrella patens subsp. patens].  
162696780         PC-Esterase                                                                       PHYPADRAFT_64155       374    Physcomitrella patens subsp. patens             viridiplantae                        predicted protein [Physcomitrella patens subsp. patens].  
15232907          SIG+PC-Esterase                                                                   AT3G02440              373    Arabidopsis thaliana                            viridiplantae                        unknown protein [Arabidopsis thaliana].  
168001319         PC-Esterase                                                                       PHYPADRAFT_114063      373    Physcomitrella patens subsp. patens             viridiplantae                        predicted protein [Physcomitrella patens subsp. patens].  
162663624         SIG+PC-Esterase                                                                   PHYPADRAFT_154405      372    Physcomitrella patens subsp. patens             viridiplantae                        predicted protein [Physcomitrella patens subsp. patens].  
15224585          SIG+PC-Esterase                                                                   AT2G30900              367    Arabidopsis thaliana                            viridiplantae                        unknown protein [Arabidopsis thaliana].  
18406003          SIG+PC-Esterase                                                                   AT2G42570              367    Arabidopsis thaliana                            viridiplantae                        unknown protein [Arabidopsis thaliana].  
162664357         PC-Esterase                                                                       PHYPADRAFT_3108        366    Physcomitrella patens subsp. patens             viridiplantae                        predicted protein [Physcomitrella patens subsp. patens].  
145329999         SIG+PC-Esterase                                                                   AT2G31110              364    Arabidopsis thaliana                            viridiplantae                        unknown protein [Arabidopsis thaliana].  
Smoe1000007554    PC-Esterase                                                                       Smoe1000007554         363    Selaginella moellendorffii                      viridiplantae                        e_gw1.22.250.1  
15219157          SIG+PC-Esterase                                                                   AT1G78710              359    Arabidopsis thaliana                            viridiplantae                        unknown protein [Arabidopsis thaliana].  
Smoe1000003183    PC-Esterase                                                                       Smoe1000003183         360    Selaginella moellendorffii                      viridiplantae                        e_gw1.20.914.1  
168040504         PC-Esterase                                                                       PHYPADRAFT_139125      356    Physcomitrella patens subsp. patens             viridiplantae                        predicted protein [Physcomitrella patens subsp. patens].  
79607892          SIG+PC-Esterase                                                                   AT3G14850              356    Arabidopsis thaliana                            viridiplantae                        unknown protein [Arabidopsis thaliana].  
Smoe1000004758    PC-Esterase                                                                       Smoe1000004758         357    Selaginella moellendorffii                      viridiplantae                        e_gw1.40.446.1  
162679974         PC-Esterase                                                                       PHYPADRAFT_133790      355    Physcomitrella patens subsp. patens             viridiplantae                        predicted protein [Physcomitrella patens subsp. patens].  
168060613         PC-Esterase                                                                       PHYPADRAFT_151024      355    Physcomitrella patens subsp. patens             viridiplantae                        predicted protein [Physcomitrella patens subsp. patens].  
162692147         PC-Esterase                                                                       PHYPADRAFT_118212      354    Physcomitrella patens subsp. patens             viridiplantae                        predicted protein [Physcomitrella patens subsp. patens].  
Smoe1000003085    PC-Esterase                                                                       Smoe1000003085         355    Selaginella moellendorffii                      viridiplantae                        e_gw1.46.172.1  
168066018         PC-Esterase                                                                       PHYPADRAFT_3322        349    Physcomitrella patens subsp. patens             viridiplantae                        predicted protein [Physcomitrella patens subsp. patens].  
162666878         PC-Esterase                                                                       PHYPADRAFT_3045        348    Physcomitrella patens subsp. patens             viridiplantae                        predicted protein [Physcomitrella patens subsp. patens].  
168020753         PC-Esterase                                                                       PHYPADRAFT_13939       347    Physcomitrella patens subsp. patens             viridiplantae                        predicted protein [Physcomitrella patens subsp. patens].  
Smoe1000019021    PC-Esterase                                                                       Smoe1000019021         348    Selaginella moellendorffii                      viridiplantae                        e_gw1.0.2313.1  
167999356         PC-Esterase                                                                       PHYPADRAFT_22778       346    Physcomitrella patens subsp. patens             viridiplantae                        predicted protein [Physcomitrella patens subsp. patens].  
18397465          TM+PC-Esterase                                                                    AT3G06080              346    Arabidopsis thaliana                            viridiplantae                        unknown protein [Arabidopsis thaliana].  
Smoe1000012530    PC-Esterase                                                                       Smoe1000012530         346    Selaginella moellendorffii                      viridiplantae                        gw1.2.1319.1  
Smoe1000012898    PC-Esterase                                                                       Smoe1000012898         346    Selaginella moellendorffii                      viridiplantae                        gw1.2.1320.1  
162669960         PC-Esterase                                                                       PHYPADRAFT_3812        343    Physcomitrella patens subsp. patens             viridiplantae                        predicted protein [Physcomitrella patens subsp. patens].  
Smoe1000017519    PC-Esterase                                                                       Smoe1000017519         344    Selaginella moellendorffii                      viridiplantae                        gw1.140.6.1  
Smoe1000015575    PC-Esterase                                                                       Smoe1000015575         342    Selaginella moellendorffii                      viridiplantae                        e_gw1.3.678.1  
Smoe1000007765    PC-Esterase                                                                       Smoe1000007765         336    Selaginella moellendorffii                      viridiplantae                        gw1.22.382.1  
Smoe1000015715    PC-Esterase                                                                       Smoe1000015715         336    Selaginella moellendorffii                      viridiplantae                        gw1.3.1219.1  
Smoe1000019442    PC-Esterase                                                                       Smoe1000019442         330    Selaginella moellendorffii                      viridiplantae                        gw1.18.628.1  
Smoe1000009081    PC-Esterase                                                                       Smoe1000009081         328    Selaginella moellendorffii                      viridiplantae                        gw1.114.75.1  
30698028          SIG+PC-Esterase                                                                   AT5G64470              325    Arabidopsis thaliana                            viridiplantae                        unknown protein [Arabidopsis thaliana].  
168063364         PC-Esterase                                                                       PHYPADRAFT_32643       319    Physcomitrella patens subsp. patens             viridiplantae                        predicted protein [Physcomitrella patens subsp. patens].  
42573730          SIG+PC-Esterase                                                                   PMR5                   291    Arabidopsis thaliana                            viridiplantae                        PMR5 (POWDERY MILDEW RESISTANT 5) [Arabidopsis thaliana].  
42569501          PC-Esterase                                                                       AT2G31110              216    Arabidopsis thaliana                            viridiplantae                        unknown protein [Arabidopsis thaliana].  
# 4;  
Chlo1000003137    PC-Esterase                                                                       Chlo1000003137         495    Chlorella sp.                                   viridiplantae>chlorophyta            IGS.gm_2_00282  
Chlo1000005972    PC-Esterase                                                                       Chlo1000005972         324    Chlorella sp.                                   viridiplantae>chlorophyta            IGS.gm_7_00195  
Chlo1000005971    PC-Esterase                                                                       Chlo1000005971         295    Chlorella sp.                                   viridiplantae>chlorophyta            IGS.gm_7_00194  
Chlo1000003832    PC-Esterase                                                                       Chlo1000003832         278    Chlorella sp.                                   viridiplantae>chlorophyta            IGS.gm_4_00392  
Chlo1000005952    SIG+PC-Esterase                                                                   Chlo1000005952         902    Chlorella sp.                                   viridiplantae>chlorophyta            IGS.gm_7_00172  
Chlo1000006983    SIG+PC-Esterase                                                                   Chlo1000006983         1210   Chlorella sp.                                   viridiplantae>chlorophyta            fgenesh3_pg.C_scaffold_8000066  
Chlo1000003272    SIG+PC-Esterase                                                                   Chlo1000003272         822    Chlorella sp.                                   viridiplantae>chlorophyta            IGS.gm_2_00433  
Chlo1000003273    BetaPropeller+BetaPropeller+PC-Esterase                                           Chlo1000003273         1149   Chlorella sp.                                   viridiplantae>chlorophyta            IGS.gm_2_00434  
  
# 3;  
170102328         SIG+PC-Esterase                                                                   LACBIDRAFT_294579      434    Laccaria bicolor S238N-H82                      fungi>basidiomycota                  predicted protein [Laccaria bicolor S238N-H82].  
164646146         SIG+PC-Esterase                                                                   LACBIDRAFT_293629      420    Laccaria bicolor S238N-H82                      fungi>basidiomycota                  predicted protein [Laccaria bicolor S238N-H82].  
170102448         SIG+PC-Esterase                                                                   LACBIDRAFT_299437      415    Laccaria bicolor S238N-H82                      fungi>basidiomycota                  predicted protein [Laccaria bicolor S238N-H82].  
164643361         PC-Esterase                                                                       LACBIDRAFT_327997      559    Laccaria bicolor S238N-H82                      fungi>basidiomycota                  predicted protein [Laccaria bicolor S238N-H82].  
170101562         PC-Esterase                                                                       LACBIDRAFT_298714      254    Laccaria bicolor S238N-H82                      fungi>basidiomycota                  predicted protein [Laccaria bicolor S238N-H82].  
169850176         PC-Esterase                                                                       CC1G_05884             153    Coprinopsis cinerea okayama7#130                fungi>basidiomycota                  predicted protein [Coprinopsis cinerea okayama7#130].  
169866565         SIG+PC-Esterase                                                                   CC1G_06059             504    Coprinopsis cinerea okayama7#130                fungi>basidiomycota                  predicted protein [Coprinopsis cinerea okayama7#130].  
169850172         SIG+PC-Esterase                                                                   CC1G_05882             502    Coprinopsis cinerea okayama7#130                fungi>basidiomycota                  predicted protein [Coprinopsis cinerea okayama7#130].  
164642644         SIG+PC-Esterase                                                                   LACBIDRAFT_328244      276    Laccaria bicolor S238N-H82                      fungi>basidiomycota                  predicted protein [Laccaria bicolor S238N-H82].  
Pbla1000009355    SIG+PC-Esterase                                                                   Pbla1000009355         445    Phycomyces blakesleeanus                        fungi>basal fungal lineages          fgeneshPB_pg.29__90  
164643157         SIG+Cas1p                                                                         LACBIDRAFT_298567      119    Laccaria bicolor S238N-H82                      fungi>basidiomycota                  predicted protein [Laccaria bicolor S238N-H82].       
164633880         SIG+Cas1p                                                                         LACBIDRAFT_303639      330    Laccaria bicolor S238N-H82                      fungi>basidiomycota                  predicted protein [Laccaria bicolor S238N-H82].       
164646578         SIG+Cas1p                                                                         LACBIDRAFT_293315      439    Laccaria bicolor S238N-H82                      fungi>basidiomycota                  predicted protein [Laccaria bicolor S238N-H82].       
116507074         SIG+Cas1p                                                                         CC1G_05885             1380   Coprinopsis cinerea okayama7#130                fungi>basidiomycota                  predicted protein [Coprinopsis cinerea okayama7#130].  
217402674         SIG+Cas1p                                                                         PHATRDRAFT_bd1441      325    Phaeodactylum tricornutum CCAP 1055/1           stramenopiles                        predicted protein [Phaeodactylum tricornutum CCAP 1055/1].  
# 2;  
217402673         SIG+PC-Esterase                                                                   PHATRDRAFT_bd1732      483    Phaeodactylum tricornutum CCAP 1055/1           stramenopiles                        predicted protein [Phaeodactylum tricornutum CCAP 1055/1].  
217412034         SIG+PC-Esterase                                                                   PHATRDRAFT_43221       472    Phaeodactylum tricornutum CCAP 1055/1           stramenopiles                        predicted protein [Phaeodactylum tricornutum CCAP 1055/1].  
Ehux1000007894    SIG+PC-Esterase                                                                   Ehux1000007894         334    Emiliania huxleyi                               haptophyceae                         gm1.400020  
220968491         SIG+PC-Esterase                                                                   THAPSDRAFT_10529       374    Thalassiosira pseudonana CCMP1335               stramenopiles                        predicted protein [Thalassiosira pseudonana CCMP1335].  
Ehux1000011410    SIG+TM+PC-Esterase                                                                Ehux1000011410         641    Emiliania huxleyi                               haptophyceae                         fgeneshEH_pg.103__23  
Ehux1000014527    PC-Esterase                                                                       Ehux1000014527         458    Emiliania huxleyi                               haptophyceae                         gm1.2800068  
Ehux1000000874    PC-Esterase                                                                       Ehux1000000874         452    Emiliania huxleyi                               haptophyceae                         fgeneshEH_pg.72__27  
Ehux1000020171    PC-Esterase                                                                       Ehux1000020171         252    Emiliania huxleyi                               haptophyceae                         gm1.10900097  
  
  
##############################  
  
# 28; CASD1   ; PC-Esterase+ Acyltransferase TM region  
46103822          SIG+TM+TM+TM+TM+TM+TM+PC-Esterase+TM+TM+TM+TM+TM+TM+TM+TM+TM+TM                   FG00106.1              1232   Gibberella zeae PH-1                            fungi>ascomycota                     hypothetical protein FG00106.1 [Gibberella zeae PH-1].  
46114564          SIG+PC-Esterase+TM+TM+TM+TM+TM+TM+TM+TM+TM+TM                                     FG03124.1              851    Gibberella zeae PH-1                            fungi>ascomycota                     hypothetical protein FG03124.1 [Gibberella zeae PH-1].  
46138121          TM+TM+PC-Esterase+TM+TM+TM+TM+TM+TM+TM+TM+TM+TM+TM                                FG10575.1              934    Gibberella zeae PH-1                            fungi>ascomycota                     hypothetical protein FG10575.1 [Gibberella zeae PH-1].  
46115520          SIG+PC-Esterase+TM+TM+TM+TM+TM+TM+TM+TM+TM+TM+TM+TM+TM+TM+TM+TM+TM+TM+TM+TM+TM    FG03602.1              1454   Gibberella zeae PH-1                            fungi>ascomycota                     hypothetical protein FG03602.1 [Gibberella zeae PH-1].  
46127017          SIG+PC-Esterase+TM+TM+TM+TM+TM+TM+TM+TM+TM+TM                                     FG07886.1              861    Gibberella zeae PH-1                            fungi>ascomycota                     hypothetical protein FG07886.1 [Gibberella zeae PH-1].  
50547609          SIG+PC-Esterase+TM+TM+TM+TM+TM+TM+TM+TM+TM+TM+TM+TM+TM                            YALI0C00187g           834    Yarrowia lipolytica CLIB122                     fungi>ascomycota                     YALI0C00187p [Yarrowia lipolytica].  
Pbla1000006496    SIG+PC-Esterase+TM+TM+TM+TM+TM+TM+TM+TM+TM+TM+TM+TM                               Pbla1000006496         884    Phycomyces blakesleeanus                        fungi>basal fungal lineages          fgeneshPB_pg.8__377  
169843844         PC-Esterase+TM+TM+TM+TM+TM+TM+TM+TM+TM                                            CC1G_10518             782    Coprinopsis cinerea okayama7#130                fungi>basidiomycota                  hypothetical protein CC1G_10518 [Coprinopsis cinerea okayama7#130].  
58262436          SIG+PC-Esterase+TM+TM+TM+TM+TM+TM+TM+TM+TM+TM+TM                                  CNN01530               960    Cryptococcus neoformans var. neoformans JEC21   fungi>basidiomycota                  O-acetyltransferase [Cryptococcus neoformans var. neoformans  
Ppla1000007369    PC-Esterase+TM+TM+TM+TM+TM+TM+TM+TM+TM+TM+TM                                      Ppla1000007369         810    Postia placenta                                 fungi>basidiomycota                  estExt_Genewise1Plus.C_1110127  
Ppla1000003910    PC-Esterase+TM+TM+TM+TM+TM+TM+TM+TM+TM+TM                                         Ppla1000003910         1350   Postia placenta                                 fungi>basidiomycota                  estExt_fgenesh3_pg.C_1530005  
210128365         PC-Esterase+TM+TM+TM+TM+TM+TM+TM+TM+TM+TM+TM+TM                                   BRAFLDRAFT_66940       633    Branchiostoma floridae                          metazoa                              hypothetical protein BRAFLDRAFT_66940 [Branchiostoma floridae].  
219498463         PC-Esterase+TM+TM+TM+TM+TM+TM+TM                                                  BRAFLDRAFT_107904      571    Branchiostoma floridae                          metazoa                              hypothetical protein BRAFLDRAFT_107904 [Branchiostoma floridae].  
198430770         SIG+PC-Esterase+TM+TM+TM+TM+TM+TM+TM+TM+TM+TM+TM+TM+TM                            LOC100177911           803    Ciona intestinalis                              metazoa                              PREDICTED: similar to CAS1 domain-containing protein 1 [Ciona  
Caps1000021394    TM+PC-Esterase+TM+TM+TM+TM+TM+TM+TM+TM+TM+TM+TM+TM+TM                             Caps1000021394         790    Capitella spI                                   metazoa>annelida                     estExt_Genewise1Plus.C_3810043  
Hrob1000020574    SIG+PC-Esterase+TM+TM+TM+TM+TM+TM+TM+TM+TM+TM+TM+TM                               Hrob1000020574         741    Helobdella robusta                              metazoa>annelida                     89851  
156406044         TM+PC-Esterase+TM+TM+TM+TM+TM+TM+TM+TM+TM+TM                                      NEMVEDRAFT_v1g238466   725    Nematostella vectensis                          metazoa>cnidaria                     predicted protein [Nematostella vectensis].  
Dpul1000014677    TM+PC-Esterase+TM+TM+TM+TM+TM+TM+TM+TM+TM+TM+TM+TM                                Dpul1000014677         820    Daphnia pulex                                   metazoa>crustacea                    e_gw1.17.170.1  
158302105         TM+PC-Esterase+TM+TM+TM+TM+TM+TM+TM+TM+TM+TM+TM+TM+TM                             AgaP_AGAP001402        791    Anopheles gambiae str. PEST                     metazoa>hexapoda                     AGAP001402-PA [Anopheles gambiae str. PEST].  
66549058          SIG+PC-Esterase+TM+TM+TM+TM+TM+TM+TM+TM+TM+TM+TM+TM                               LOC411555              791    Apis mellifera                                  metazoa>hexapoda                     PREDICTED: similar to CG2938-PB [Apis mellifera].  
189235703         PC-Esterase+TM+TM+TM+TM+TM+TM+TM+TM+TM+TM+TM+TM+TM                                LOC655926              796    Tribolium castaneum                             metazoa>hexapoda                     PREDICTED: similar to CG2938 CG2938-PB [Tribolium castaneum].  
18543323          PC-Esterase+TM+TM+TM+TM+TM+TM+TM+TM+TM+TM+TM+TM+TM                                CG2938                 862    Drosophila melanogaster                         metazoa>hexapoda                     CG2938 [Drosophila melanogaster].  
Lgig1000008388    TM+PC-Esterase+TM+TM+TM+TM+TM+TM+TM+TM+TM+TM+TM+TM+TM                             Lgig1000008388         723    Lottia gigantea                                 metazoa>mollusca                     fgenesh2_pm.C_sca_107000001  
118085840         PC-Esterase+TM+TM+TM+TM+TM+TM+TM+TM+TM+TM+TM+TM                                   CASD1                  881    Gallus gallus                                   metazoa>vertebrata                   PREDICTED: CAS1 domain containing 1 [Gallus gallus].  
40255037          SIG+PC-Esterase+TM+TM+TM+TM+TM+TM+TM+TM+TM+TM+TM+TM                               CASD1                  797    Homo sapiens                                    metazoa>vertebrata                   CAS1 domain containing 1 [Homo sapiens].  
149254923         SIG+PC-Esterase+TM+TM+TM+TM+TM+TM+TM+TM+TM+TM+TM+TM                               LOC100045658           906    Mus musculus                                    metazoa>vertebrata                   PREDICTED: similar to O-acetyltransferase [Mus musculus].  
37620157          SIG+PC-Esterase+TM+TM+TM+TM+TM+TM+TM+TM+TM+TM+TM+TM                               Casd1                  797    Mus musculus                                    metazoa>vertebrata                   CAS1 domain containing 1 [Mus musculus].  
109473087         PC-Esterase+TM+TM+TM+TM+TM+TM+TM+TM+TM+TM+TM+TM                                   LOC678772              715    Rattus norvegicus                               metazoa>vertebrata                   PREDICTED: similar to O-acetyltransferase [Rattus norvegicus].  
114614562         SIG+PC-Esterase+TM+TM+TM+TM+TM+TM+TM+TM+TM+TM+TM+TM                               CASD1                  797    Pan troglodytes                                 metazoa>vertebrata                   PREDICTED: CAS1 domain containing 1 isoform 3 [Pan troglodytes].  
109473084         SIG+PC-Esterase                                                                   LOC684310              257    Rattus norvegicus                               metazoa>vertebrata                   PREDICTED: similar to O-acetyltransferase [Rattus norvegicus].  
113675490         SIG+PC-Esterase+TM                                                                casd1                  417    Danio rerio                                     metazoa>vertebrata>actinopterygii    CAS1 domain containing 1 [Danio rerio].  
Aano1000005821    SIG+TM+TM+PC-Esterase+TM+TM+TM+TM+TM+TM+TM+TM+TM                                  Aano1000005821         1935   Aureococcus anophagefferens                     stramenopiles                        67109  
Aano1000006974    EP1+PC-Esterase+CYSARC+TM+TM+TM+TM+TM+TM+TM+TM                                    Aano1000006974         1682   Aureococcus anophagefferens                     stramenopiles                        65873  
220975182         SIG+TM+TM+TM+TM+TM+TM+TM+TM+TM+PC-Esterase+TM                                     THAPSDRAFT_4842        1171   Thalassiosira pseudonana CCMP1335               stramenopiles                        predicted protein [Thalassiosira pseudonana CCMP1335].  
221117967         PIPSQUEAK+PC-Esterase+TM+TM+TM+TM+TM+TM+EGF+EGF+EGF+EGF+EGF+EGF                   LOC100206436           1389   Hydra magnipapillata                            metazoa>cnidaria                     PREDICTED: similar to predicted protein, partial [Hydra  
# 16;  
Dpul1000018099    SIG+PC-Esterase                                                                   Dpul1000018099         493    Daphnia pulex                                   metazoa>crustacea                    fgenesh1_pg.C_scaffold_18000354  
Dpul1000022492    SIG+PC-Esterase                                                                   Dpul1000022492         477    Daphnia pulex                                   metazoa>crustacea                    fgenesh1_pg.C_scaffold_91000061  
Dpul1000022491    PC-Esterase                                                                       Dpul1000022491         429    Daphnia pulex                                   metazoa>crustacea                    fgenesh1_pg.C_scaffold_91000060  
Dpul1000008143    SIG+TM+PC-Esterase                                                                Dpul1000008143         407    Daphnia pulex                                   metazoa>crustacea                    SNAP_00021797  
Dpul1000011583    TM+PC-Esterase                                                                    Dpul1000011583         385    Daphnia pulex                                   metazoa>crustacea                    NCBI_GNO_3300100  
Dpul1000013547    SIG+PC-Esterase                                                                   Dpul1000013547         381    Daphnia pulex                                   metazoa>crustacea                    fgenesh1_pg.C_scaffold_14000173  
Dpul1000025636    TM+PC-Esterase                                                                    Dpul1000025636         365    Daphnia pulex                                   metazoa>crustacea                    fgenesh1_pg.C_scaffold_202000002  
Dpul1000016338    PC-Esterase                                                                       Dpul1000016338         362    Daphnia pulex                                   metazoa>crustacea                    NCBI_GNO_3600107  
Dpul1000008083    PC-Esterase                                                                       Dpul1000008083         357    Daphnia pulex                                   metazoa>crustacea                    NCBI_GNO_7200001  
Dpul1000013294    SIG+PC-Esterase                                                                   Dpul1000013294         327    Daphnia pulex                                   metazoa>crustacea                    SNAP_00018351  
Dpul1000023224    PC-Esterase                                                                       Dpul1000023224         322    Daphnia pulex                                   metazoa>crustacea                    SNAP_00030716  
Dpul1000016869    PC-Esterase                                                                       Dpul1000016869         308    Daphnia pulex                                   metazoa>crustacea                    SNAP_00028986  
Dpul1000016874    PC-Esterase                                                                       Dpul1000016874         302    Daphnia pulex                                   metazoa>crustacea                    SNAP_00028991  
Dpul1000025643    PC-Esterase                                                                       Dpul1000025643         292    Daphnia pulex                                   metazoa>crustacea                    NCBI_GNO_59400001  
Dpul1000029909    PC-Esterase                                                                       Dpul1000029909         291    Daphnia pulex                                   metazoa>crustacea                    NCBI_GNO_537600001  
Dpul1000008133    SIG+PC-Esterase                                                                   Dpul1000008133         240    Daphnia pulex                                   metazoa>crustacea                    SNAP_00021789  
Dpul1000016870    PC-Esterase+PC-Esterase                                                           Dpul1000016870         695    Daphnia pulex                                   metazoa>crustacea                    NCBI_GNO_14500019  
Dpul1000008125    PC-Esterase                                                                       Dpul1000008125         627    Daphnia pulex                                   metazoa>crustacea                    NCBI_GNO_7200048  
  
  
# 3; AT3G03210 close to both casD and  plant group  
Chlo1000005955    SIG+PC-Esterase                                                                   Chlo1000005955         329    Chlorella sp.                                   viridiplantae>chlorophyta            IGS.gm_7_00176  
Chlo1000005973    PC-Esterase                                                                       Chlo1000005973         304    Chlorella sp.                                   viridiplantae>chlorophyta            IGS.gm_7_00196  
15228492          SIG+PC-Esterase                                                                   AT3G03210              368    Arabidopsis thaliana                            viridiplantae                        unknown protein [Arabidopsis thaliana].  
162673705         SIG+PC-Esterase                                                                   PHYPADRAFT_192656      356    Physcomitrella patens subsp. patens             viridiplantae                        predicted protein [Physcomitrella patens subsp. patens].  
Smoe1000013052    SIG+PC-Esterase                                                                   Smoe1000013052         351    Selaginella moellendorffii                      viridiplantae                        fgenesh2_pg.C_scaffold_26000257  
Mpus1000005283    PC-Esterase                                                                       Mpus1000005283         431    Micromonas pusilla                              viridiplantae>chlorophyta            50466  
  
  
# 28;lineage specific expansion weakly related to CASD1  
Caps1000008608    TM+PC-Esterase                                                                    Caps1000008608         537    Capitella spI                                   metazoa>annelida                     fgenesh1_pg.C_scaffold_511000023  
Caps1000002416    SIG+PC-Esterase                                                                   Caps1000002416         534    Capitella spI                                   metazoa>annelida                     fgenesh1_pg.C_scaffold_86000057  
Caps1000022838    SIG+PC-Esterase                                                                   Caps1000022838         520    Capitella spI                                   metazoa>annelida                     fgenesh1_pg.C_scaffold_90000032  
Caps1000022265    TM+PC-Esterase                                                                    Caps1000022265         465    Capitella spI                                   metazoa>annelida                     fgenesh1_pg.C_scaffold_119000023  
Caps1000024579    TM+PC-Esterase                                                                    Caps1000024579         458    Capitella spI                                   metazoa>annelida                     fgenesh1_pg.C_scaffold_808000006  
Caps1000005556    SIG+PC-Esterase                                                                   Caps1000005556         454    Capitella spI                                   metazoa>annelida                     fgenesh1_pg.C_scaffold_416000028  
Caps1000022609    TM+PC-Esterase                                                                    Caps1000022609         440    Capitella spI                                   metazoa>annelida                     fgenesh1_pg.C_scaffold_319000007  
Caps1000021759    PC-Esterase                                                                       Caps1000021759         430    Capitella spI                                   metazoa>annelida                     fgenesh1_pg.C_scaffold_132000027  
Caps1000022328    SIG+PC-Esterase                                                                   Caps1000022328         409    Capitella spI                                   metazoa>annelida                     fgenesh1_pg.C_scaffold_457000008  
Caps1000009109    SIG+PC-Esterase                                                                   Caps1000009109         407    Capitella spI                                   metazoa>annelida                     fgenesh1_pg.C_scaffold_148000007  
Caps1000009397    SIG+PC-Esterase                                                                   Caps1000009397         405    Capitella spI                                   metazoa>annelida                     fgenesh1_pg.C_scaffold_153000040  
Caps1000017976    SIG+PC-Esterase                                                                   Caps1000017976         405    Capitella spI                                   metazoa>annelida                     fgenesh1_pg.C_scaffold_64000045  
Caps1000009171    TM+PC-Esterase                                                                    Caps1000009171         397    Capitella spI                                   metazoa>annelida                     fgenesh1_pg.C_scaffold_369000011  
Caps1000031591    SIG+PC-Esterase                                                                   Caps1000031591         392    Capitella spI                                   metazoa>annelida                     fgenesh1_pg.C_scaffold_2894000001  
Caps1000019102    SIG+PC-Esterase                                                                   Caps1000019102         388    Capitella spI                                   metazoa>annelida                     fgenesh1_pg.C_scaffold_290000019  
Caps1000032411    SIG+PC-Esterase                                                                   Caps1000032411         387    Capitella spI                                   metazoa>annelida                     fgenesh1_pg.C_scaffold_2016000001  
Caps1000028887    SIG+PC-Esterase                                                                   Caps1000028887         385    Capitella spI                                   metazoa>annelida                     fgenesh1_pg.C_scaffold_1385000001  
Caps1000015517    SIG+PC-Esterase                                                                   Caps1000015517         383    Capitella spI                                   metazoa>annelida                     fgenesh1_pg.C_scaffold_209000001  
Caps1000006425    PC-Esterase                                                                       Caps1000006425         379    Capitella spI                                   metazoa>annelida                     fgenesh1_pg.C_scaffold_15000013  
Caps1000016805    PC-Esterase                                                                       Caps1000016805         377    Capitella spI                                   metazoa>annelida                     fgenesh1_pg.C_scaffold_989000004  
Caps1000004655    SIG+PC-Esterase                                                                   Caps1000004655         375    Capitella spI                                   metazoa>annelida                     fgenesh1_pg.C_scaffold_412000024  
Caps1000005306    SIG+PC-Esterase                                                                   Caps1000005306         373    Capitella spI                                   metazoa>annelida                     estExt_fgenesh1_pg.C_3660020  
Caps1000012525    SIG+PC-Esterase                                                                   Caps1000012525         352    Capitella spI                                   metazoa>annelida                     fgenesh1_pg.C_scaffold_1162000002  
Caps1000027478    PC-Esterase                                                                       Caps1000027478         351    Capitella spI                                   metazoa>annelida                     fgenesh1_pg.C_scaffold_970000005  
Caps1000011075    SIG+PC-Esterase                                                                   Caps1000011075         348    Capitella spI                                   metazoa>annelida                     fgenesh1_pg.C_scaffold_196000001  
Caps1000008731    PC-Esterase                                                                       Caps1000008731         336    Capitella spI                                   metazoa>annelida                     fgenesh1_pg.C_scaffold_112000041  
Caps1000031747    SIG+PC-Esterase                                                                   Caps1000031747         302    Capitella spI                                   metazoa>annelida                     fgenesh1_pg.C_scaffold_24598000001  
Caps1000028836    PC-Esterase                                                                       Caps1000028836         218    Capitella spI                                   metazoa>annelida                     fgenesh1_pg.C_scaffold_960000001  
Caps1000006140    SIG+PC-Esterase                                                                   Caps1000006140         431    Capitella spI                                   metazoa>annelida                     fgenesh1_pg.C_scaffold_529000017  
Caps1000003886    PC-Esterase                                                                       Caps1000003886         397    Capitella spI                                   metazoa>annelida                     fgenesh1_pg.C_scaffold_264000025  
Caps1000002929    SIG+PC-Esterase                                                                   Caps1000002929         384    Capitella spI                                   metazoa>annelida                     fgenesh1_pg.C_scaffold_339000005  
Caps1000006137    SIG+PC-Esterase                                                                   Caps1000006137         381    Capitella spI                                   metazoa>annelida                     fgenesh1_pg.C_scaffold_529000013  
Caps1000019155    SIG+PC-Esterase                                                                   Caps1000019155         366    Capitella spI                                   metazoa>annelida                     fgenesh1_pg.C_scaffold_168000013  
Caps1000016408    TM+PC-Esterase                                                                    Caps1000016408         365    Capitella spI                                   metazoa>annelida                     fgenesh1_pg.C_scaffold_175000014  
Caps1000000360    SIG+PC-Esterase                                                                   Caps1000000360         369    Capitella spI                                   metazoa>annelida                     fgenesh1_pg.C_scaffold_22000027  
Caps1000016114    SIG+PC-Esterase                                                                   Caps1000016114         365    Capitella spI                                   metazoa>annelida                     fgenesh1_pg.C_scaffold_494000002  
Caps1000016122    SIG+PC-Esterase                                                                   Caps1000016122         893    Capitella spI                                   metazoa>annelida                     fgenesh1_pg.C_scaffold_494000010  
  
  
# 7; TTL C7orf58  FLJ21986  
109471780         SIG+AALigase+CadL+PC-Esterase                                                     LOC500046              1030   Rattus norvegicus                               metazoa>vertebrata                   PREDICTED: hypothetical protein LOC500046 [Rattus norvegicus].  
109473163         SIG+AALigase+CadL+PC-Esterase                                                     LOC500046              1026   Rattus norvegicus                               metazoa>vertebrata                   PREDICTED: hypothetical protein LOC500046 [Rattus norvegicus].  
114615647         SIG+AALigase+CadL+PC-Esterase                                                     LOC472491              1026   Pan troglodytes                                 metazoa>vertebrata                   PREDICTED: hypothetical protein isoform 2 [Pan troglodytes].  
124486851         SIG+AALigase+CadL+PC-Esterase                                                     A430107O13Rik          1026   Mus musculus                                    metazoa>vertebrata                   hypothetical protein LOC214642 [Mus musculus].  
157671945         SIG+AALigase+CadL+PC-Esterase                                                     C7orf58                1026   Homo sapiens                                    metazoa>vertebrata                   hypothetical protein LOC79974 isoform 1 [Homo sapiens].  
Lgig1000015543    AALigase+CadL+PC-Esterase                                                         Lgig1000015543         1005   Lottia gigantea                                 metazoa>mollusca                     fgenesh2_pg.C_sca_186000009  
125814246         SIG+AALigase+CadL+PC-Esterase                                                     si:ch211-152m4.1       962    Danio rerio                                     metazoa>vertebrata>actinopterygii    PREDICTED: hypothetical protein [Danio rerio].  
219461483         SIG+AALigase+CadL+PC-Esterase                                                     BRAFLDRAFT_91002       1242   Branchiostoma floridae                          metazoa                              hypothetical protein BRAFLDRAFT_91002 [Branchiostoma floridae].  
Dpul1000011236    CadL+PC-Esterase                                                                  Dpul1000011236         546    Daphnia pulex                                   metazoa>crustacea                    SNAP_00008543  
118082244         SIG+PC-Esterase                                                                   LOC770375              419    Gallus gallus                                   metazoa>vertebrata                   PREDICTED: hypothetical protein [Gallus gallus].  
Caps1000000871    CadL+PC-Esterase                                                                  Caps1000000871         725    Capitella spI                                   metazoa>annelida                     fgenesh1_pg.C_scaffold_113000017  
  
  
# 3; close to both CASD  and plants  
Chlo1000004187    SIG+PC-Esterase                                                                   Chlo1000004187         396    Chlorella sp.                                   viridiplantae>chlorophyta            IGS.gm_20_00197  
Chlo1000006644    PC-Esterase                                                                       Chlo1000006644         339    Chlorella sp.                                   viridiplantae>chlorophyta            IGS.gm_5_00440  
Chlo1000001667    PC-Esterase                                                                       Chlo1000001667         290    Chlorella sp.                                   viridiplantae>chlorophyta            IGS.gm_3_00656  
Ehux1000003059    SIG+PC-Esterase                                                                   Ehux1000003059         352    Emiliania huxleyi                               haptophyceae                         gm1.4700003  
159470759         PC-Esterase                                                                       CHLREDRAFT_168604      305    Chlamydomonas reinhardtii                       viridiplantae>chlorophyta            predicted protein [Chlamydomonas reinhardtii].  
159470757         PC-Esterase                                                                       CHLREDRAFT_187971      158    Chlamydomonas reinhardtii                       viridiplantae>chlorophyta            predicted protein [Chlamydomonas reinhardtii].  
217410569         PC-Esterase                                                                       PHATRDRAFT_32775       354    Phaeodactylum tricornutum CCAP 1055/1           stramenopiles                        predicted protein [Phaeodactylum tricornutum CCAP 1055/1].  
217410090         SIG+PC-Esterase                                                                   PHATRDRAFT_44612       353    Phaeodactylum tricornutum CCAP 1055/1           stramenopiles                        predicted protein [Phaeodactylum tricornutum CCAP 1055/1].  
158283025         SIG+PC-Esterase                                                                   CHLREDRAFT_187970      636    Chlamydomonas reinhardtii                       viridiplantae>chlorophyta            predicted protein [Chlamydomonas reinhardtii].  
158283028         SIG+PC-Esterase                                                                   CHLREDRAFT_187972      239    Chlamydomonas reinhardtii                       viridiplantae>chlorophyta            predicted protein [Chlamydomonas reinhardtii].  
  
  
  
##########  
# 28; Fam113   ; closest to bacterial GDSL and brings Cas1 first   ; called sgnh-hydrolase in psiblast  
198425831         PC-Esterase                                                                       LOC100187110           427    Ciona intestinalis                              metazoa                              PREDICTED: similar to Protein FAM113B [Ciona intestinalis].  
210101990         PC-Esterase                                                                       BRAFLDRAFT_126870      422    Branchiostoma floridae                          metazoa                              hypothetical protein BRAFLDRAFT_126870 [Branchiostoma floridae].  
219465461         PC-Esterase                                                                       BRAFLDRAFT_126881      422    Branchiostoma floridae                          metazoa                              hypothetical protein BRAFLDRAFT_126881 [Branchiostoma floridae].  
Caps1000014196    PC-Esterase                                                                       Caps1000014196         255    Capitella spI                                   metazoa>annelida                     gw1.95.80.1  
Hrob1000012151    PC-Esterase                                                                       Hrob1000012151         272    Helobdella robusta                              metazoa>annelida                     78799  
156217549         PC-Esterase                                                                       NEMVEDRAFT_v1g113662   267    Nematostella vectensis                          metazoa>cnidaria                     predicted protein [Nematostella vectensis].  
156228303         PC-Esterase                                                                       NEMVEDRAFT_v1g81187    191    Nematostella vectensis                          metazoa>cnidaria                     predicted protein [Nematostella vectensis].  
115637265         PC-Esterase                                                                       LOC581481              464    Strongylocentrotus purpuratus                   metazoa>echinodermata                PREDICTED: similar to A930025D01Rik protein [Strongylocentrotus  
Lgig1000005008    PC-Esterase                                                                       Lgig1000005008         252    Lottia gigantea                                 metazoa>mollusca                     e_gw1.47.73.1  
Lgig1000017428    PC-Esterase                                                                       Lgig1000017428         270    Lottia gigantea                                 metazoa>mollusca                     estExt_Genewise1.C_sca_210185  
17506393          PC-Esterase                                                                       D2030.8                550    Caenorhabditis elegans                          metazoa>nematoda                     D2030.8 [Caenorhabditis elegans].  
30520223          PC-Esterase                                                                       A930025D01Rik          296    Mus musculus                                    metazoa>vertebrata                   hypothetical protein LOC319513 [Mus musculus].  
114583141         PC-Esterase                                                                       LOC739478              404    Pan troglodytes                                 metazoa>vertebrata                   PREDICTED: similar to A930025D01Rik protein [Pan troglodytes].  
114680621         PC-Esterase                                                                       LOC458050              403    Pan troglodytes                                 metazoa>vertebrata                   PREDICTED: similar to OTTHUMP00000030057 isoform 6 [Pan  
86515434          PC-Esterase                                                                       Fam113b                432    Rattus norvegicus                               metazoa>vertebrata                   hypothetical protein LOC315283 [Rattus norvegicus].  
82879339          PC-Esterase                                                                       EG241084               402    Mus musculus                                    metazoa>vertebrata                   PREDICTED: similar to hCG1645016 [Mus musculus].  
114645086         PC-Esterase                                                                       LOC452421              399    Pan troglodytes                                 metazoa>vertebrata                   PREDICTED: similar to Family with sequence similarity 113, member B  
114680627         PC-Esterase                                                                       LOC458050              396    Pan troglodytes                                 metazoa>vertebrata                   PREDICTED: similar to OTTHUMP00000030059 isoform 3 [Pan  
114680625         PC-Esterase                                                                       LOC458050              317    Pan troglodytes                                 metazoa>vertebrata                   PREDICTED: similar to OTTHUMP00000030060 isoform 2 [Pan  
114680623         PC-Esterase                                                                       LOC458050              312    Pan troglodytes                                 metazoa>vertebrata                   PREDICTED: similar to OTTHUMP00000030060 isoform 1 [Pan  
62655312          PC-Esterase                                                                       RGD1562431_predicted   402    Rattus norvegicus                               metazoa>vertebrata                   PREDICTED: similar to Protein FAM113A [Rattus norvegicus].  
59891421          PC-Esterase                                                                       Fam113a                296    Rattus norvegicus                               metazoa>vertebrata                   hypothetical protein LOC296158 [Rattus norvegicus].  
19923901          PC-Esterase                                                                       FAM113B                432    Homo sapiens                                    metazoa>vertebrata                   hypothetical protein LOC91523 [Homo sapiens].  
114645092         PC-Esterase                                                                       LOC452421              432    Pan troglodytes                                 metazoa>vertebrata                   PREDICTED: similar to Family with sequence similarity 113, member B  
26986609          PC-Esterase                                                                       Fam113b                433    Mus musculus                                    metazoa>vertebrata                   hypothetical protein LOC239647 [Mus musculus].  
114680617         PC-Esterase                                                                       LOC458050              450    Pan troglodytes                                 metazoa>vertebrata                   PREDICTED: similar to OTTHUMP00000030059 isoform 7 [Pan  
21362096          PC-Esterase                                                                       FAM113A                454    Homo sapiens                                    metazoa>vertebrata                   hypothetical protein LOC64773 [Homo sapiens].  
114680611         PC-Esterase                                                                       LOC458050              454    Pan troglodytes                                 metazoa>vertebrata                   PREDICTED: similar to OTTHUMP00000030059 isoform 4 [Pan  
56693271          PC-Esterase                                                                       zgc:103558             415    Danio rerio                                     metazoa>vertebrata>actinopterygii    hypothetical protein LOC494057 [Danio rerio].  
  
# 2;  
46109690          SIG+PC-Esterase                                                                   FG01727.1              468    Gibberella zeae PH-1                            fungi>ascomycota                     hypothetical protein FG01727.1 [Gibberella zeae PH-1].  
50552069          SIG+PC-Esterase                                                                   YALI0E03696g           411    Yarrowia lipolytica CLIB122                     fungi>ascomycota                     YALI0E03696p [Yarrowia lipolytica].  
71021513          SIG+PC-Esterase                                                                   UM04840.1              451    Ustilago maydis 521                             fungi>basidiomycota                  hypothetical protein UM04840.1 [Ustilago maydis 521].  
46126319          SIG+PC-Esterase                                                                   FG07537.1              450    Gibberella zeae PH-1                            fungi>ascomycota                     hypothetical protein FG07537.1 [Gibberella zeae PH-1].  
  
######  
# 20;AFUA_2G11920  
71001686          SIG+TM+TM+PC-Esterase                                                             AFUA_2G11920           438    Aspergillus fumigatus Af293                     fungi>ascomycota                     conserved hypothetical protein [Aspergillus fumigatus Af293].  
67540076          SIG+TM+PC-Esterase                                                                AN6208.2               407    Aspergillus nidulans FGSC A4                    fungi>ascomycota                     hypothetical protein AN6208.2 [Aspergillus nidulans FGSC A4].  
198419756         EGF2+PC-Esterase                                                                  LOC100182421           407    Ciona intestinalis                              metazoa                              PREDICTED: similar to predicted protein [Ciona intestinalis].  
198431391         SIG+EGF2+PC-Esterase                                                              LOC100175255           424    Ciona intestinalis                              metazoa                              PREDICTED: similar to predicted protein [Ciona intestinalis].  
198413458         SIG+EGF2+PC-Esterase                                                              LOC100185235           451    Ciona intestinalis                              metazoa                              PREDICTED: hypothetical protein [Ciona intestinalis].  
198437264         PC-Esterase                                                                       LOC494393              246    Ciona intestinalis                              metazoa                              PREDICTED: hypothetical protein LOC494393 [Ciona intestinalis].  
198413476         EGF2+PC-Esterase                                                                  LOC100180470           297    Ciona intestinalis                              metazoa                              PREDICTED: hypothetical protein [Ciona intestinalis].  
198431393         SIG+EGF2+PC-Esterase                                                              LOC100176800           426    Ciona intestinalis                              metazoa                              PREDICTED: similar to predicted protein [Ciona intestinalis].  
221106242         EGF2+PC-Esterase                                                                  LOC100215631           376    Hydra magnipapillata                            metazoa>cnidaria                     PREDICTED: similar to predicted protein [Hydra magnipapillata].  
Lgig1000013638    SIG+EGF2+PC-Esterase                                                              Lgig1000013638         416    Lottia gigantea                                 metazoa>mollusca                     fgenesh2_pg.C_sca_71000104  
Lgig1000020382    SIG+EGF2+PC-Esterase                                                              Lgig1000020382         413    Lottia gigantea                                 metazoa>mollusca                     estExt_fgenesh2_pg.C_sca_30249  
Lgig1000015812    TM+EGF2+PC-Esterase                                                               Lgig1000015812         530    Lottia gigantea                                 metazoa>mollusca                     fgenesh2_pg.C_sca_226000003  
Lgig1000008571    EGF2+PC-Esterase                                                                  Lgig1000008571         379    Lottia gigantea                                 metazoa>mollusca                     fgenesh2_pg.C_sca_1000612  
Lgig1000021783    EGF2+PC-Esterase                                                                  Lgig1000021783         345    Lottia gigantea                                 metazoa>mollusca                     estExt_fgenesh2_pg.C_sca_340025  
Lgig1000022293    SIG+EGF2+PC-Esterase                                                              Lgig1000022293         477    Lottia gigantea                                 metazoa>mollusca                     estExt_fgenesh2_pg.C_sca_540087  
Lgig1000020212    SIG+EGF2+PC-Esterase                                                              Lgig1000020212         372    Lottia gigantea                                 metazoa>mollusca                     estExt_fgenesh2_pg.C_sca_10586  
Lgig1000013597    SIG+EGF2+PC-Esterase+TM                                                           Lgig1000013597         590    Lottia gigantea                                 metazoa>mollusca                     fgenesh2_pg.C_sca_70000015  
156223878         TM+EGF2+PC-Esterase                                                               NEMVEDRAFT_v1g240880   428    Nematostella vectensis                          metazoa>cnidaria                     predicted protein [Nematostella vectensis].  
156216361         TM+EGF2+PC-Esterase                                                               NEMVEDRAFT_v1g211833   435    Nematostella vectensis                          metazoa>cnidaria                     predicted protein [Nematostella vectensis].  
156208791         EGF2+PC-Esterase                                                                  NEMVEDRAFT_v1g220927   568    Nematostella vectensis                          metazoa>cnidaria                     predicted protein [Nematostella vectensis].  
156228297         SIG+EGF2+PC-Esterase                                                              NEMVEDRAFT_v1g197481   338    Nematostella vectensis                          metazoa>cnidaria                     predicted protein [Nematostella vectensis].  
156219926         SIG+EGF2+PC-Esterase                                                              NEMVEDRAFT_v1g207406   419    Nematostella vectensis                          metazoa>cnidaria                     predicted protein [Nematostella vectensis].  
# 3;  
Ehux1000003316    SIG+PC-Esterase                                                                   Ehux1000003316         395    Emiliania huxleyi                               haptophyceae                         fgeneshEH_pg.340__2  
Ehux1000008654    SIG+PC-Esterase                                                                   Ehux1000008654         395    Emiliania huxleyi                               haptophyceae                         fgeneshEH_pg.437__15  
Ehux1000022107    PC-Esterase                                                                       Ehux1000022107         389    Emiliania huxleyi                               haptophyceae                         fgeneshEH_pg.92__47  
220969263         SIG+PC-Esterase                                                                   THAPSDRAFT_11718       723    Thalassiosira pseudonana CCMP1335               stramenopiles                        predicted protein [Thalassiosira pseudonana CCMP1335].  
220968600         PC-Esterase                                                                       THAPSDRAFT_10472       448    Thalassiosira pseudonana CCMP1335               stramenopiles                        predicted protein [Thalassiosira pseudonana CCMP1335].  
Aano1000005846    SIG+Cas1p                                                                         Aano1000005846         1979   Aureococcus anophagefferens                     stramenopiles  
Mpus1000007031    SIG+PC-Esterase                                                                   Mpus1000007031         355    Micromonas pusilla                              viridiplantae>chlorophyta            54883  
  
  
# 2;N terminal IG fold [close to fam55] + PC-Esterase domain close to above group 156223878  
156227833         SIG+ig_filamin+PC-Esterase                                                        NEMVEDRAFT_v1g197924   521    Nematostella vectensis                          metazoa>cnidaria                     predicted protein [Nematostella vectensis].  
156214610         SIG+ig_filamin+PC-Esterase                                                        NEMVEDRAFT_v1g213968   492    Nematostella vectensis                          metazoa>cnidaria                     predicted protein [Nematostella vectensis].  
221127246         ig_filamin+PC-Esterase                                                            LOC100208485           409    Hydra magnipapillata                            metazoa>cnidaria                     PREDICTED: similar to predicted protein [Hydra magnipapillata].  
156405266         SIG+ig_filamin+PC-Esterase                                                        NEMVEDRAFT_v1g238639   448    Nematostella vectensis                          metazoa>cnidaria                     predicted protein [Nematostella vectensis].  
156219285         SIG+ig_filamin+PC-Esterase                                                        NEMVEDRAFT_v1g208207   937    Nematostella vectensis                          metazoa>cnidaria                     predicted protein [Nematostella vectensis].  
156341438         SIG+ig_filamin+PC-Esterase                                                        NEMVEDRAFT_v1g222736   548    Nematostella vectensis                          metazoa>cnidaria                     hypothetical protein NEMVEDRAFT_v1g222736 [Nematostella vectensis].  
156219312         SIG+ig_filamin+PC-Esterase                                                        NEMVEDRAFT_v1g208161   457    Nematostella vectensis                          metazoa>cnidaria                     predicted protein [Nematostella vectensis].  
  
  
  
  
  
######  
  
#Tail  
  
  
  
# 3;  
29345979          SIG+PC-Esterase                                                                   BT_0569                236    Bacteroides thetaiotaomicron VPI-5482           bacteroidetes/chlorobi               hypothetical protein BT_0569 [Bacteroides thetaiotaomicron  
53715273          SIG+PC-Esterase                                                                   BF3989                 235    Bacteroides fragilis YCH46                      bacteroidetes/chlorobi               hypothetical protein BF3989 [Bacteroides fragilis YCH46].  
60683209          SIG+PC-Esterase                                                                   BF3764                 235    Bacteroides fragilis NCTC 9343                  bacteroidetes/chlorobi               putative acylhydrolase [Bacteroides fragilis NCTC 9343].  
# 2; Acyltransferase TM region +GDSL hydrolase huge family  
120405825         SIG+TM+TM+TM+TM+TM+TM+TM+TM+TM+TM+PC-Esterase                                     Mvan_4875              671    Mycobacterium vanbaalenii PYR-1                 actinobacteria                       acyltransferase 3 [Mycobacterium vanbaalenii PYR-1].  
118467801         SIG+TM+TM+TM+TM+TM+TM+TM+TM+TM+TM+PC-Esterase                                     MSMEG_5537             669    Mycobacterium smegmatis str. MC2 155            actinobacteria                       integral membrane protein [Mycobacterium smegmatis str. MC2 155].  
# 2;  
Ehux1000025433    PC-Esterase                                                                       Ehux1000025433         629    Emiliania huxleyi                               haptophyceae                         fgeneshEH_pg.73__118  
Ehux1000008326    PC-Esterase                                                                       Ehux1000008326         452    Emiliania huxleyi                               haptophyceae                         gm1.36400025  
  
  
# 2;  
Ehux1000026725    SIG+PC-Esterase                                                                   Ehux1000026725         426    Emiliania huxleyi                               haptophyceae                         fgeneshEH_pg.62__107  
Ehux1000016382    SIG+PC-Esterase                                                                   Ehux1000016382         401    Emiliania huxleyi                               haptophyceae                         gm1.5500080  
# 2;  
Ppla1000007194    PC-Esterase                                                                       Ppla1000007194         379    Postia placenta                                 fungi>basidiomycota                  e_gw1.9.37.1  
Ppla1000013272    PC-Esterase                                                                       Ppla1000013272         379    Postia placenta                                 fungi>basidiomycota                  estExt_Genewise1Plus.C_2250021  
  
# 2;  
210081384         ig_filamin+PC-Esterase+ig_filamin+PC-Esterase                                           BRAFLDRAFT_112234      932    Branchiostoma floridae                          metazoa                              hypothetical protein BRAFLDRAFT_112234 [Branchiostoma floridae].  
219457802         ig_filamin+PC-Esterase+ig_filamin+PC-Esterase                                           BRAFLDRAFT_89063       918    Branchiostoma floridae                          metazoa                              hypothetical protein BRAFLDRAFT_89063 [Branchiostoma floridae].  
#  
Ehux1000007696    SIG+ig_filamin+PC-Esterase                                                        Ehux1000007696         590    Emiliania huxleyi                               haptophyceae                         fgeneshEH_pg.347__8  
210109857         TM+ig_filamin+RICIN+PC-Esterase                                                   BRAFLDRAFT_85021       834    Branchiostoma floridae                          metazoa                              hypothetical protein BRAFLDRAFT_85021 [Branchiostoma floridae].  
198418464         SIG+ig_filamin+PC-Esterase+TM+ig_filamin+PC-Esterase                                    LOC100185934           1122   Ciona intestinalis                              metazoa                              PREDICTED: similar to Protein FAM55A [Ciona intestinalis].  
115641139         ig_filamin+PC-Esterase                                                            LOC585234              508    Strongylocentrotus purpuratus                   metazoa>echinodermata                PREDICTED: similar to protein of unknown function, partial  
115973862         SIG+ig_filamin+PC-Esterase                                                        LOC759252              494    Strongylocentrotus purpuratus                   metazoa>echinodermata                PREDICTED: similar to protein of unknown function, partial  
115903751         ig_filamin+PC-Esterase+IG+IG+TM                                                   LOC753163              909    Strongylocentrotus purpuratus                   metazoa>echinodermata                PREDICTED: hypothetical protein, partial [Strongylocentrotus  
189536541         ig_filamin+PC-Esterase+ANK+TM+TM+TM+TM+TM+TM+TM+TM                                trpc1                  1119   Danio rerio                                     metazoa>vertebrata>actinopterygii    PREDICTED: transient receptor potential cation channel, subfamily  
Mpus1000009923    SIG+ig_filamin+PC-Esterase                                                        Mpus1000009923         510    Micromonas pusilla                              viridiplantae>chlorophyta            66048  
  
  
# 1;  
  
116626424         PC-Esterase                                                                       Acid_7386              179    Solibacter usitatus Ellin6076                   fibrobacteres/acidobacteria          putative transmembrane protein [Solibacter usitatus Ellin6076].  
42781593          PC-Esterase                                                                       BCE_2532               197    Bacillus cereus ATCC 10987                      firmicutes                           lipase/acylhydrolase, putative [Bacillus cereus ATCC 10987].  
90023634          SIG+PC-Esterase                                                                   Sde_3994               256    Saccharophagus degradans 2-40                   proteobacteria>gammaproteobacteria   hypothetical protein Sde_3994 [Saccharophagus degradans 2-40].  
Ehux1000019754    PC-Esterase                                                                       Ehux1000019754         724    Emiliania huxleyi                               haptophyceae                         fgeneshEH_pg.26__53  
Ehux1000023490    PC-Esterase                                                                       Ehux1000023490         854    Emiliania huxleyi                               haptophyceae                         gm1.1800171  
Ehux1000008237    PC-Esterase+TM                                                                    Ehux1000008237         636    Emiliania huxleyi                               haptophyceae                         gm1.18900012  
Ehux1000002595    PC-Esterase                                                                       Ehux1000002595         528    Emiliania huxleyi                               haptophyceae                         fgeneshEH_pg.292__3  
Ehux1000002851    PC-Esterase                                                                       Ehux1000002851         978    Emiliania huxleyi                               haptophyceae                         gm1.7200003  
Ngru1000005053    SIG+PC-Esterase                                                                   Ngru1000005053         714    Naegleria gruberi                               heterolobosea                        estExt_fgeneshHS_pg.C_480001  
219425448         PC-Esterase                                                                       BRAFLDRAFT_72966       537    Branchiostoma floridae                          metazoa                              hypothetical protein BRAFLDRAFT_72966 [Branchiostoma floridae].  
210113856         PC-Esterase                                                                       BRAFLDRAFT_81003       220    Branchiostoma floridae                          metazoa                              hypothetical protein BRAFLDRAFT_81003 [Branchiostoma floridae].  
210110880         PC-Esterase+TM                                                                    BRAFLDRAFT_84021       978    Branchiostoma floridae                          metazoa                              hypothetical protein BRAFLDRAFT_84021 [Branchiostoma floridae].  
Caps1000000923    PC-Esterase                                                                       Caps1000000923         233    Capitella spI                                   metazoa>annelida                     fgenesh1_pg.C_scaffold_286000022  
221129927         PC-Esterase                                                                       LOC100211039           1003   Hydra magnipapillata                            metazoa>cnidaria                     PREDICTED: similar to Protein FAM113B [Hydra magnipapillata].  
Dpul1000016873    PC-Esterase                                                                       Dpul1000016873         425    Daphnia pulex                                   metazoa>crustacea                    NCBI_GNO_14500022  
115680340         PC-Esterase                                                                       LOC577736              260    Strongylocentrotus purpuratus                   metazoa>echinodermata                PREDICTED: hypothetical protein, partial [Strongylocentrotus  
109505801         PC-Esterase+H1                                                                    LOC684681              318    Rattus norvegicus                               metazoa>vertebrata                   PREDICTED: similar to Histone H1.2 (H1 VAR.1) (H1c) [Rattus  
Aano1000004415    PC-Esterase                                                                       Aano1000004415         495    Aureococcus anophagefferens                     stramenopiles                        63775  
Aano1000005269    SIG+PC-Esterase                                                                   Aano1000005269         476    Aureococcus anophagefferens                     stramenopiles                        62992  
Aano1000010689    SIG+PC-Esterase                                                                   Aano1000010689         476    Aureococcus anophagefferens                     stramenopiles                        68766  
Aano1000005550    SIG+PC-Esterase                                                                   Aano1000005550         459    Aureococcus anophagefferens                     stramenopiles                        63201  
Aano1000003295    PC-Esterase                                                                       Aano1000003295         1784   Aureococcus anophagefferens                     stramenopiles                        71237  
Aano1000003714    SIG+PC-Esterase                                                                   Aano1000003714         601    Aureococcus anophagefferens                     stramenopiles                        72339  
Aano1000001844    SIG+PC-Esterase+PH+C2+C2+TM+EFHAND+TM+TM+TM                                       Aano1000001844         2411   Aureococcus anophagefferens                     stramenopiles                        62341  
Aano1000004418    PC-Esterase                                                                       Aano1000004418         1760   Aureococcus anophagefferens                     stramenopiles                        71531  
Aano1000004637    SIG+PC-Esterase+PEP-utilisers                                                     Aano1000004637         2336   Aureococcus anophagefferens                     stramenopiles                        71577  
Aano1000005182    SIG+PC-Esterase                                                                   Aano1000005182         917    Aureococcus anophagefferens                     stramenopiles                        62926  
Aano1000002618    PC-Esterase+TM                                                                    Aano1000002618         1424   Aureococcus anophagefferens                     stramenopiles                        70752  
Psoj1000010319    SIG+PC-Esterase                                                                   Psoj1000010319         866    Phytophthora sojae                              stramenopiles                        137517  
220976969         SIG+PC-Esterase                                                                   THAPSDRAFT_2497        396    Thalassiosira pseudonana CCMP1335               stramenopiles                        predicted protein [Thalassiosira pseudonana CCMP1335].  
220969965         SIG+PC-Esterase                                                                   THAPSDRAFT_25239       659    Thalassiosira pseudonana CCMP1335               stramenopiles                        predicted protein [Thalassiosira pseudonana CCMP1335].  
209586057         SIG+PC-Esterase                                                                   THAPS_6661             740    Thalassiosira pseudonana CCMP1335               stramenopiles                        predicted protein [Thalassiosira pseudonana CCMP1335].  
18404664          PC-Esterase                                                                       AT2G38180              312    Arabidopsis thaliana                            viridiplantae                        GDSL-motif lipase/hydrolase family protein [Arabidopsis thaliana].  
159467311         PC-Esterase                                                                       CHLREDRAFT_145397      293    Chlamydomonas reinhardtii                       viridiplantae>chlorophyta            predicted protein [Chlamydomonas reinhardtii].  
116000292         PC-Esterase                                                                       Ot01g00610             319    Ostreococcus tauri                              viridiplantae>chlorophyta            Isoamyl acetate-hydrolyzing esterase (ISS) [Ostreococcus tauri].  
Vcar1000015234    SIG+PC-Esterase                                                                   Vcar1000015234         603    Volvox carteri                                  viridiplantae>chlorophyta            fgenesh4_pg.C_scaffold_925000001  
  
Top
```

---

Family
clusters of 10TM Acyltransferases with
domain
architecture  
  

```
# 535; 10TMAct+GDSL oatA; B and Ce exp  
Ehux1000010509    10TMAct+TM+GDSL                                                                            Ehux1000010509           799    Emiliania huxleyi                                                     haptophyceae                              gm1.22800027  
Caps1000031763    10TMAct                                                                                    Caps1000031763           319    Capitella spI                                                         metazoa>annelida                          fgenesh1_pg.C_scaffold_35363000001  
17559158          10TMAct+TM+GDSL                                                                            oac-12                   643    Caenorhabditis elegans                                                metazoa>nematoda                          O-ACyltransferase homolog family member (oac-12) [Caenorhabditis  
17508997          10TMAct+GDSL                                                                               T09E11.5                 578    Caenorhabditis elegans                                                metazoa>nematoda                          T09E11.5 [Caenorhabditis elegans].  
17543924          SIG+10TMAct+TM+GDSL                                                                        oac-56                   644    Caenorhabditis elegans                                                metazoa>nematoda                          O-ACyltransferase homolog family member (oac-56) [Caenorhabditis  
17509169          10TMAct+TM+GDSL                                                                            T22H2.2                  527    Caenorhabditis elegans                                                metazoa>nematoda                          T22H2.2 [Caenorhabditis elegans].  
17532481          10TMAct+TM+GDSL                                                                            oac-11                   662    Caenorhabditis elegans                                                metazoa>nematoda                          O-ACyltransferase homolog family member (oac-11) [Caenorhabditis  
71990417          SIG+10TMAct+TM+GDSL                                                                        oac-37                   659    Caenorhabditis elegans                                                metazoa>nematoda                          O-ACyltransferase homolog family member (oac-37) [Caenorhabditis  
17509001          10TMAct+TM+GDSL                                                                            oac-45                   663    Caenorhabditis elegans                                                metazoa>nematoda                          O-ACyltransferase homolog family member (oac-45) [Caenorhabditis  
17507733          10TMAct+TM+TM+GDSL                                                                         oac-35                   663    Caenorhabditis elegans                                                metazoa>nematoda                          O-ACyltransferase homolog family member (oac-35) [Caenorhabditis  
17562856          SIG+10TMAct+GDSL                                                                           oac-40                   642    Caenorhabditis elegans                                                metazoa>nematoda                          O-ACyltransferase homolog family member (oac-40) [Caenorhabditis  
17562864          SIG+10TMAct+TM+GDSL                                                                        oac-41                   645    Caenorhabditis elegans                                                metazoa>nematoda                          O-ACyltransferase homolog family member (oac-41) [Caenorhabditis  
17507169          10TMAct+TM+GDSL                                                                            F35E2.7                  633    Caenorhabditis elegans                                                metazoa>nematoda                          F35E2.7 [Caenorhabditis elegans].  
17507735          10TMAct+TM+GDSL                                                                            oac-36                   668    Caenorhabditis elegans                                                metazoa>nematoda                          O-ACyltransferase homolog family member (oac-36) [Caenorhabditis  
17563972          10TMAct+TM+GDSL                                                                            T06C12.8                 669    Caenorhabditis elegans                                                metazoa>nematoda                          T06C12.8 [Caenorhabditis elegans].  
17562866          SIG+10TMAct+GDSL                                                                           R03H4.6                  646    Caenorhabditis elegans                                                metazoa>nematoda                          hypothetical protein R03H4.6 [Caenorhabditis elegans].  
86562056          SIG+10TMAct+TM+GDSL                                                                        oac-30                   647    Caenorhabditis elegans                                                metazoa>nematoda                          O-ACyltransferase homolog family member (oac-30) [Caenorhabditis  
32567150          SIG+10TMAct+TM+TM                                                                          oac-24                   617    Caenorhabditis elegans                                                metazoa>nematoda                          O-ACyltransferase homolog family member (oac-24) [Caenorhabditis  
71990679          10TMAct+TM+GDSL                                                                            T09E11.4                 671    Caenorhabditis elegans                                                metazoa>nematoda                          T09E11.4 [Caenorhabditis elegans].  
71983744          10TMAct+TM+GDSL                                                                            oac-16                   672    Caenorhabditis elegans                                                metazoa>nematoda                          O-ACyltransferase homolog family member (oac-16) [Caenorhabditis  
17507299          10TMAct+TM+GDSL                                                                            oac-25                   673    Caenorhabditis elegans                                                metazoa>nematoda                          O-ACyltransferase homolog family member (oac-25) [Caenorhabditis  
71984481          10TMAct+TM+TM+GDSL                                                                         oac-8                    679    Caenorhabditis elegans                                                metazoa>nematoda                          O-ACyltransferase homolog family member (oac-8) [Caenorhabditis  
17507283          10TMAct+TM+GDSL                                                                            oac-26                   682    Caenorhabditis elegans                                                metazoa>nematoda                          O-ACyltransferase homolog family member (oac-26) [Caenorhabditis  
17540232          10TMAct+TM+GDSL                                                                            oac-23                   685    Caenorhabditis elegans                                                metazoa>nematoda                          O-ACyltransferase homolog family member (oac-23) [Caenorhabditis  
71988046          10TMAct+TM+GDSL                                                                            oac-22                   686    Caenorhabditis elegans                                                metazoa>nematoda                          O-ACyltransferase homolog family member (oac-22) [Caenorhabditis  
17506479          10TMAct+TM+GDSL                                                                            E03H4.7                  624    Caenorhabditis elegans                                                metazoa>nematoda                          E03H4.7 [Caenorhabditis elegans].  
17542538          10TMAct+TM+GDSL                                                                            oac-48                   689    Caenorhabditis elegans                                                metazoa>nematoda                          O-ACyltransferase homolog family member (oac-48) [Caenorhabditis  
17541412          10TMAct+TM+GDSL                                                                            oac-57                   692    Caenorhabditis elegans                                                metazoa>nematoda                          O-ACyltransferase homolog family member (oac-57) [Caenorhabditis  
17541414          10TMAct+TM+GDSL                                                                            oac-58                   695    Caenorhabditis elegans                                                metazoa>nematoda                          O-ACyltransferase homolog family member (oac-58) [Caenorhabditis  
17560876          SIG+10TMAct+TM+GDSL                                                                        oac-29                   656    Caenorhabditis elegans                                                metazoa>nematoda                          O-ACyltransferase homolog family member (oac-29) [Caenorhabditis  
17559544          SIG+10TMAct+TM+GDSL                                                                        oac-15                   659    Caenorhabditis elegans                                                metazoa>nematoda                          O-ACyltransferase homolog family member (oac-15) [Caenorhabditis  
17507167          10TMAct+TM+GDSL                                                                            oac-19                   698    Caenorhabditis elegans                                                metazoa>nematoda                          O-ACyltransferase homolog family member (oac-19) [Caenorhabditis  
17542762          10TMAct+TM+GDSL                                                                            oac-52                   705    Caenorhabditis elegans                                                metazoa>nematoda                          O-ACyltransferase homolog family member (oac-52) [Caenorhabditis  
17539118          10TMAct+TM+GDSL                                                                            C42C1.7                  710    Caenorhabditis elegans                                                metazoa>nematoda                          hypothetical protein C42C1.7 [Caenorhabditis elegans].  
17541016          10TMAct+GDSL                                                                               oac-38                   607    Caenorhabditis elegans                                                metazoa>nematoda                          O-ACyltransferase homolog family member (oac-38) [Caenorhabditis  
17531875          SIG+10TMAct+TM+GDSL                                                                        oac-4                    731    Caenorhabditis elegans                                                metazoa>nematoda                          O-ACyltransferase homolog family member (oac-4) [Caenorhabditis  
17507287          10TMAct+GDSL                                                                               oac-27                   669    Caenorhabditis elegans                                                metazoa>nematoda                          O-ACyltransferase homolog family member (oac-27) [Caenorhabditis  
17564748          10TMAct+TM+GDSL                                                                            T26H2.7                  848    Caenorhabditis elegans                                                metazoa>nematoda                          T26H2.7 [Caenorhabditis elegans].  
71981930          SIG+10TMAct+GDSL+TM                                                                        B0399.2                  890    Caenorhabditis elegans                                                metazoa>nematoda                          B0399.2 [Caenorhabditis elegans].  
71982248          SIG+10TMAct+TM+GDSL                                                                        oac-2                    658    Caenorhabditis elegans                                                metazoa>nematoda                          O-ACyltransferase homolog family member (oac-2) [Caenorhabditis  
17506687          10TMAct+TM+GDSL+TM+CLECTIN                                                                 oac-17                   919    Caenorhabditis elegans                                                metazoa>nematoda                          O-ACyltransferase homolog family member (oac-17) [Caenorhabditis  
17507697          SIG+10TMAct+TM+GDSL+TM+TM+TM+TM+TM+TM+TM                                                   F56G4.1                  975    Caenorhabditis elegans                                                metazoa>nematoda                          F56G4.1 [Caenorhabditis elegans].  
17541014          10TMAct+GDSL                                                                               oac-38                   596    Caenorhabditis elegans                                                metazoa>nematoda                          O-ACyltransferase homolog family member (oac-38) [Caenorhabditis  
117928664         10TMAct+TM+GDSL                                                                            Acel_1457                645    Acidothermus cellulolyticus 11B                                       actinobacteria                            acyltransferase 3 [Acidothermus cellulolyticus 11B].  
119962466         10TMAct+TM+GDSL                                                                            AAur_2868                694    Arthrobacter aurescens TC1                                            actinobacteria                            acyltransferase domain-containing protein [Arthrobacter aurescens  
119962008         10TMAct+TM+GDSL                                                                            AAur_2869                700    Arthrobacter aurescens TC1                                            actinobacteria                            acyltransferase domain-containing protein [Arthrobacter aurescens  
119961022         SIG+10TMAct+TM+GDSL                                                                        AAur_2425                694    Arthrobacter aurescens TC1                                            actinobacteria                            acyltransferase family protein [Arthrobacter aurescens TC1].  
116671426         SIG+10TMAct+GDSL                                                                           Arth_2880                681    Arthrobacter sp. FB24                                                 actinobacteria                            acyltransferase 3 [Arthrobacter sp. FB24].  
116671735         10TMAct                                                                                    Arth_3190                357    Arthrobacter sp. FB24                                                 actinobacteria                            acyltransferase 3 [Arthrobacter sp. FB24].  
116671236         10TMAct+TM+GDSL                                                                            Arth_2690                715    Arthrobacter sp. FB24                                                 actinobacteria                            acyltransferase 3 [Arthrobacter sp. FB24].  
116671233         SIG+10TMAct+TM+GDSL                                                                        Arth_2687                734    Arthrobacter sp. FB24                                                 actinobacteria                            acyltransferase 3 [Arthrobacter sp. FB24].  
116671734         10TMAct+TM+GDSL                                                                            Arth_3189                688    Arthrobacter sp. FB24                                                 actinobacteria                            acyltransferase 3 [Arthrobacter sp. FB24].  
116670550         10TMAct                                                                                    Arth_2002                382    Arthrobacter sp. FB24                                                 actinobacteria                            acyltransferase 3 [Arthrobacter sp. FB24].  
116671427         SIG+10TMAct+TM+GDSL                                                                        Arth_2881                684    Arthrobacter sp. FB24                                                 actinobacteria                            acyltransferase 3 [Arthrobacter sp. FB24].  
119025708         10TMAct+GDSL                                                                               BAD_0690                 606    Bifidobacterium adolescentis ATCC 15703                               actinobacteria                            membrane protein with acetylase function [Bifidobacterium  
189439579         10TMAct+TM+GDSL                                                                            BLD_0716                 606    Bifidobacterium longum DJO10A                                         actinobacteria                            Putative acyltransferase [Bifidobacterium longum DJO10A].  
189439483         SIG+10TMAct+TM+TM                                                                          BLD_0620                 264    Bifidobacterium longum DJO10A                                         actinobacteria                            Putative acyltransferase [Bifidobacterium longum DJO10A].  
23465459          SIG+10TMAct+TM+GDSL                                                                        BL0885                   619    Bifidobacterium longum NCC2705                                        actinobacteria                            hypothetical protein BL0885 [Bifidobacterium longum NCC2705].  
23465534          SIG+10TMAct+TM+GDSL                                                                        BL0962                   624    Bifidobacterium longum NCC2705                                        actinobacteria                            membrane protein with acetylase function [Bifidobacterium longum  
148272779         SIG+10TMAct+TM+GDSL                                                                        wcmG                     730    Clavibacter michiganensis subsp. michiganensis NCPPB 382              actinobacteria                            putative membrane-bound acyltranferase [Clavibacter michiganensis  
148271902         10TMAct+TM+GDSL                                                                            wcnB                     757    Clavibacter michiganensis subsp. michiganensis NCPPB 382              actinobacteria                            putative acyltransferase [Clavibacter michiganensis subsp.  
170782598         10TMAct+TM+GDSL                                                                            CMS_2255                 759    Clavibacter michiganensis subsp. sepedonicus                          actinobacteria                            putative acyltransferase [Clavibacter michiganensis subsp.  
170781971         10TMAct+TM+GDSL                                                                            CMS_1579                 811    Clavibacter michiganensis subsp. sepedonicus                          actinobacteria                            putative acyltransferase [Clavibacter michiganensis subsp.  
38233259          10TMAct+TM+GDSL                                                                            DIP0652                  705    Corynebacterium diphtheriae NCTC 13129                                actinobacteria                            hypothetical protein DIP0652 [Corynebacterium diphtheriae NCTC  
38234788          SIG+10TMAct+TM+GDSL                                                                        DIP2252                  597    Corynebacterium diphtheriae NCTC 13129                                actinobacteria                            putative integral membrane protein [Corynebacterium diphtheriae  
38234718          10TMAct                                                                                    DIP2175                  379    Corynebacterium diphtheriae NCTC 13129                                actinobacteria                            putative integral membrane acyltransferase [Corynebacterium  
38234260          10TMAct+GDSL                                                                               DIP1689                  748    Corynebacterium diphtheriae NCTC 13129                                actinobacteria                            hypothetical protein DIP1689 [Corynebacterium diphtheriae NCTC  
25027212          SIG+10TMAct+TM                                                                             CE0656                   417    Corynebacterium efficiens YS-314                                      actinobacteria                            hypothetical protein CE0656 [Corynebacterium efficiens YS-314].  
25027289          10TMAct+TM+GDSL                                                                            CE0733                   712    Corynebacterium efficiens YS-314                                      actinobacteria                            hypothetical protein CE0733 [Corynebacterium efficiens YS-314].  
25029236          10TMAct                                                                                    CE2680                   384    Corynebacterium efficiens YS-314                                      actinobacteria                            hypothetical protein CE2680 [Corynebacterium efficiens YS-314].  
62391696          SIG+10TMAct                                                                                cg3163                   356    Corynebacterium glutamicum ATCC 13032                                 actinobacteria                            acyltransferase [Corynebacterium glutamicum ATCC 13032].  
19551607          10TMAct                                                                                    NCgl0350                 331    Corynebacterium glutamicum ATCC 13032                                 actinobacteria                            acyltransferase [Corynebacterium glutamicum ATCC 13032].  
62389593          10TMAct+TM+GDSL                                                                            wbpC                     716    Corynebacterium glutamicum ATCC 13032                                 actinobacteria                            lipopolysaccharide biosynthesis acyltransferase, m [Corynebacterium  
145294877         10TMAct+TM+GDSL                                                                            cgR_0824                 716    Corynebacterium glutamicum R                                          actinobacteria                            hypothetical protein cgR_0824 [Corynebacterium glutamicum R].  
145296845         SIG+10TMAct                                                                                cgR_2745                 356    Corynebacterium glutamicum R                                          actinobacteria                            hypothetical protein cgR_2745 [Corynebacterium glutamicum R].  
68536583          SIG+10TMAct+TM+GDSL                                                                        jk1497                   977    Corynebacterium jeikeium K411                                         actinobacteria                            hypothetical protein jk1497 [Corynebacterium jeikeium K411].  
68535128          10TMAct+TM+GDSL                                                                            jk0066                   798    Corynebacterium jeikeium K411                                         actinobacteria                            hypothetical protein jk0066 [Corynebacterium jeikeium K411].  
68536563          10TMAct+GDSL                                                                               jk1477                   694    Corynebacterium jeikeium K411                                         actinobacteria                            putative acyltransferase [Corynebacterium jeikeium K411].  
68535230          SIG+10TMAct                                                                                jk0165                   419    Corynebacterium jeikeium K411                                         actinobacteria                            hypothetical protein jk0165 [Corynebacterium jeikeium K411].  
172039765         10TMAct+TM+GDSL                                                                            cur_0085                 825    Corynebacterium urealyticum DSM 7109                                  actinobacteria                            hypothetical protein cur_0085 [Corynebacterium urealyticum DSM  
172039986         10TMAct+TM+GDSL                                                                            cur_0306                 641    Corynebacterium urealyticum DSM 7109                                  actinobacteria                            hypothetical protein cur_0306 [Corynebacterium urealyticum DSM  
172039864         10TMAct                                                                                    cur_0184                 430    Corynebacterium urealyticum DSM 7109                                  actinobacteria                            hypothetical protein cur_0184 [Corynebacterium urealyticum DSM  
111221223         SIG+10TMAct                                                                                FRAAL1779                629    Frankia alni ACN14a                                                   actinobacteria                            putative integral membrane acyltransferase [Frankia alni ACN14a].  
111221372         10TMAct                                                                                    FRAAL1934                443    Frankia alni ACN14a                                                   actinobacteria                            putative membrane-bound transacylase [Frankia alni ACN14a].  
111224170         SIG+10TMAct                                                                                FRAAL4780                412    Frankia alni ACN14a                                                   actinobacteria                            putative membrane-bound acyl-transferase [Frankia alni ACN14a].  
111223824         10TMAct                                                                                    FRAAL4428                489    Frankia alni ACN14a                                                   actinobacteria                            putative transmembrane acyltransferase [Frankia alni ACN14a].  
111220440         10TMAct                                                                                    FRAAL0973                933    Frankia alni ACN14a                                                   actinobacteria                            hypothetical protein FRAAL0973 [Frankia alni ACN14a].  
111221221         SIG+10TMAct+TM+GDSL                                                                        FRAAL1777                835    Frankia alni ACN14a                                                   actinobacteria                            hypothetical protein FRAAL1777 [Frankia alni ACN14a].  
86739765          10TMAct+TM+GDSL                                                                            Francci3_1054            671    Frankia sp. CcI3                                                      actinobacteria                            acyltransferase 3 [Frankia sp. CcI3].  
86739767          SIG+TM+10TMAct                                                                             Francci3_1056            561    Frankia sp. CcI3                                                      actinobacteria                            acyltransferase 3 [Frankia sp. CcI3].  
86739190          10TMAct                                                                                    Francci3_0475            713    Frankia sp. CcI3                                                      actinobacteria                            acyltransferase 3 [Frankia sp. CcI3].  
86741944          10TMAct+GDSL                                                                               Francci3_3258            725    Frankia sp. CcI3                                                      actinobacteria                            acyltransferase 3 [Frankia sp. CcI3].  
158317248         10TMAct                                                                                    Franean1_5495            587    Frankia sp. EAN1pec                                                   actinobacteria                            acyltransferase 3 [Frankia sp. EAN1pec].  
158317616         10TMAct                                                                                    Franean1_5872            435    Frankia sp. EAN1pec                                                   actinobacteria                            acyltransferase 3 [Frankia sp. EAN1pec].  
158317883         10TMAct                                                                                    Franean1_6141            799    Frankia sp. EAN1pec                                                   actinobacteria                            acyltransferase 3 [Frankia sp. EAN1pec].  
158317250         10TMAct+GDSL                                                                               Franean1_5497            977    Frankia sp. EAN1pec                                                   actinobacteria                            acyltransferase 3 [Frankia sp. EAN1pec].  
158318409         10TMAct+TM+GDSL                                                                            Franean1_6674            720    Frankia sp. EAN1pec                                                   actinobacteria                            acyltransferase 3 [Frankia sp. EAN1pec].  
157283899         10TMAct                                                                                    Krad_4584                406    Kineococcus radiotolerans SRS30216                                    actinobacteria                            acyltransferase 3 [Kineococcus radiotolerans SRS30216].  
152967603         10TMAct                                                                                    Krad_3660                387    Kineococcus radiotolerans SRS30216                                    actinobacteria                            acyltransferase 3 [Kineococcus radiotolerans SRS30216].  
184200323         10TMAct+TM+TM+GDSL                                                                         KRH_06770                692    Kocuria rhizophila DC2201                                             actinobacteria                            hypothetical protein KRH_06770 [Kocuria rhizophila DC2201].  
50954068          10TMAct+GDSL                                                                               Lxx01930                 679    Leifsonia xyli subsp. xyli str. CTCB07                                actinobacteria                            acetyltransferase [Leifsonia xyli subsp. xyli str. CTCB07].  
50955537          10TMAct+TM+GDSL                                                                            yrhL                     647    Leifsonia xyli subsp. xyli str. CTCB07                                actinobacteria                            lipopolysaccharide modification acyltransferase [Leifsonia xyli  
169629774         10TMAct+TM+GDSL                                                                            MAB_2689                 726    Mycobacterium abscessus                                               actinobacteria                            acyltransferase [Mycobacterium abscessus].  
169628495         SIG+10TMAct                                                                                MAB_1404                 398    Mycobacterium abscessus                                               actinobacteria                            acyltransferase [Mycobacterium abscessus].  
169631547         10TMAct                                                                                    MAB_4473c                392    Mycobacterium abscessus                                               actinobacteria                            acyltransferase [Mycobacterium abscessus].  
118464790         10TMAct                                                                                    MAV_4941                 393    Mycobacterium avium 104                                               actinobacteria                            acyltransferase [Mycobacterium avium 104].  
118466781         SIG+10TMAct+TM+GDSL                                                                        MAV_3209                 720    Mycobacterium avium 104                                               actinobacteria                            acyltransferase domain-containing protein [Mycobacterium avium  
118464481         10TMAct                                                                                    MAV_1402                 379    Mycobacterium avium 104                                               actinobacteria                            putative acyltransferase, putative [Mycobacterium avium 104].  
118464611         10TMAct+TM+GDSL                                                                            MAV_5201                 664    Mycobacterium avium 104                                               actinobacteria                            putative acyltransferase [Mycobacterium avium 104].  
118465263         10TMAct+GDSL                                                                               MAV_4630                 706    Mycobacterium avium 104                                               actinobacteria                            acyltransferase domain-containing protein [Mycobacterium avium  
41409765          10TMAct                                                                                    MAP3667                  408    Mycobacterium avium subsp. paratuberculosis K-10                      actinobacteria                            hypothetical protein MAP3667 [Mycobacterium avium subsp.  
41410108          10TMAct+GDSL                                                                               MAP4010c                 720    Mycobacterium avium subsp. paratuberculosis K-10                      actinobacteria                            hypothetical protein MAP4010c [Mycobacterium avium subsp.  
41408618          10TMAct                                                                                    MAP2520c                 379    Mycobacterium avium subsp. paratuberculosis K-10                      actinobacteria                            hypothetical protein MAP2520c [Mycobacterium avium subsp.  
41409612          10TMAct+TM+GDSL                                                                            MAP3514                  689    Mycobacterium avium subsp. paratuberculosis K-10                      actinobacteria                            hypothetical protein MAP3514 [Mycobacterium avium subsp.  
41407369          SIG+10TMAct+TM+GDSL                                                                        MAP1271c                 721    Mycobacterium avium subsp. paratuberculosis K-10                      actinobacteria                            hypothetical protein MAP1271c [Mycobacterium avium subsp.  
31792447          SIG+10TMAct                                                                                Mb1286                   383    Mycobacterium bovis AF2122/97                                         actinobacteria                            acyltransferase [Mycobacterium bovis AF2122/97].  
31792751          10TMAct+TM+GDSL                                                                            Mb1592c                  729    Mycobacterium bovis AF2122/97                                         actinobacteria                            hypothetical protein Mb1592c [Mycobacterium bovis AF2122/97].  
121636140         10TMAct                                                                                    BCG_0265                 407    Mycobacterium bovis BCG str. Pasteur 1173P2                           actinobacteria                            putative integral membrane acyltransferase [Mycobacterium bovis BCG  
121636024         SIG+10TMAct+TM+GDSL                                                                        BCG_0144                 685    Mycobacterium bovis BCG str. Pasteur 1173P2                           actinobacteria                            putative transmembrane acyltransferase [Mycobacterium bovis BCG  
145224444         10TMAct+TM+GDSL                                                                            Mflv_3862                694    Mycobacterium gilvum PYR-GCK                                          actinobacteria                            acyltransferase 3 [Mycobacterium gilvum PYR-GCK].  
145225357         10TMAct+TM                                                                                 Mflv_4779                428    Mycobacterium gilvum PYR-GCK                                          actinobacteria                            acyltransferase 3 [Mycobacterium gilvum PYR-GCK].  
145222449         SIG+10TMAct+TM+GDSL                                                                        Mflv_1859                671    Mycobacterium gilvum PYR-GCK                                          actinobacteria                            acyltransferase 3 [Mycobacterium gilvum PYR-GCK].  
145224214         SIG+10TMAct+TM+GDSL                                                                        Mflv_3630                731    Mycobacterium gilvum PYR-GCK                                          actinobacteria                            acyltransferase 3 [Mycobacterium gilvum PYR-GCK].  
145223408         10TMAct+TM+GDSL                                                                            Mflv_2821                728    Mycobacterium gilvum PYR-GCK                                          actinobacteria                            acyltransferase 3 [Mycobacterium gilvum PYR-GCK].  
145225935         10TMAct+TM+GDSL                                                                            Mflv_5338                728    Mycobacterium gilvum PYR-GCK                                          actinobacteria                            acyltransferase 3 [Mycobacterium gilvum PYR-GCK].  
145221024         10TMAct                                                                                    Mflv_0420                401    Mycobacterium gilvum PYR-GCK                                          actinobacteria                            acyltransferase 3 [Mycobacterium gilvum PYR-GCK].  
145222814         SIG+10TMAct                                                                                Mflv_2226                396    Mycobacterium gilvum PYR-GCK                                          actinobacteria                            acyltransferase 3 [Mycobacterium gilvum PYR-GCK].  
15828386          10TMAct                                                                                    ML2580                   384    Mycobacterium leprae TN                                               actinobacteria                            acyltransferase [Mycobacterium leprae TN].  
15827543          10TMAct                                                                                    ML1101                   379    Mycobacterium leprae TN                                               actinobacteria                            acyltransferase [Mycobacterium leprae TN].  
15828440          10TMAct+TM+GDSL                                                                            ML2670                   666    Mycobacterium leprae TN                                               actinobacteria                            integral membrane protein [Mycobacterium leprae TN].  
15827615          SIG+10TMAct+TM+GDSL                                                                        ML1213                   733    Mycobacterium leprae TN                                               actinobacteria                            hypothetical protein ML1213 [Mycobacterium leprae TN].  
183982393         SIG+10TMAct+TM+GDSL                                                                        MMAR_2380                721    Mycobacterium marinum M                                               actinobacteria                            hypothetical protein MMAR_2380 [Mycobacterium marinum M].  
183980343         10TMAct+TM+GDSL                                                                            MMAR_0312                670    Mycobacterium marinum M                                               actinobacteria                            transmembrane acyltransferase [Mycobacterium marinum M].  
183984160         10TMAct                                                                                    MMAR_4188                380    Mycobacterium marinum M                                               actinobacteria                            integral membrane acyltransferase [Mycobacterium marinum M].  
183980506         10TMAct                                                                                    MMAR_0477                388    Mycobacterium marinum M                                               actinobacteria                            integral membrane acyltransferase [Mycobacterium marinum M].  
183980879         SIG+10TMAct+TM+GDSL                                                                        MMAR_0857                720    Mycobacterium marinum M                                               actinobacteria                            transmembrane acyltransferase [Mycobacterium marinum M].  
118471295         10TMAct+TM+GDSL                                                                            MSMEG_3187               719    Mycobacterium smegmatis str. MC2 155                                  actinobacteria                            acyltransferase domain-containing protein [Mycobacterium smegmatis  
118473045         10TMAct                                                                                    MSMEG_6230               383    Mycobacterium smegmatis str. MC2 155                                  actinobacteria                            acyltransferase [Mycobacterium smegmatis str. MC2 155].  
118473105         10TMAct                                                                                    MSMEG_0319               397    Mycobacterium smegmatis str. MC2 155                                  actinobacteria                            acyltransferase [Mycobacterium smegmatis str. MC2 155].  
118468380         10TMAct+TM+GDSL                                                                            MSMEG_2021               676    Mycobacterium smegmatis str. MC2 155                                  actinobacteria                            lipopolysaccharide biosynthesis acyltransferase, M [Mycobacterium  
118470113         10TMAct+GDSL                                                                               MSMEG_0206               711    Mycobacterium smegmatis str. MC2 155                                  actinobacteria                            acyltransferase 3 [Mycobacterium smegmatis str. MC2 155].  
118467801         SIG+10TMAct+TM+GDSL                                                                        MSMEG_5537               669    Mycobacterium smegmatis str. MC2 155                                  actinobacteria                            integral membrane protein [Mycobacterium smegmatis str. MC2 155].  
118472153         10TMAct                                                                                    MSMEG_5041               382    Mycobacterium smegmatis str. MC2 155                                  actinobacteria                            acyltransferase [Mycobacterium smegmatis str. MC2 155].  
126435675         10TMAct+TM+GDSL                                                                            Mjls_3096                716    Mycobacterium sp. JLS                                                 actinobacteria                            acyltransferase 3 [Mycobacterium sp. JLS].  
126433585         10TMAct+TM                                                                                 Mjls_0976                402    Mycobacterium sp. JLS                                                 actinobacteria                            acyltransferase 3 [Mycobacterium sp. JLS].  
126432825         10TMAct                                                                                    Mjls_0212                392    Mycobacterium sp. JLS                                                 actinobacteria                            acyltransferase 3 [Mycobacterium sp. JLS].  
126433851         SIG+10TMAct                                                                                Mjls_1249                404    Mycobacterium sp. JLS                                                 actinobacteria                            acyltransferase 3 [Mycobacterium sp. JLS].  
126436555         10TMAct                                                                                    Mjls_3980                387    Mycobacterium sp. JLS                                                 actinobacteria                            acyltransferase 3 [Mycobacterium sp. JLS].  
126432734         10TMAct+TM+GDSL                                                                            Mjls_0121                726    Mycobacterium sp. JLS                                                 actinobacteria                            acyltransferase 3 [Mycobacterium sp. JLS].  
126437277         SIG+10TMAct+TM+GDSL                                                                        Mjls_4712                680    Mycobacterium sp. JLS                                                 actinobacteria                            acyltransferase 3 [Mycobacterium sp. JLS].  
119866287         10TMAct                                                                                    Mkms_0232                392    Mycobacterium sp. KMS                                                 actinobacteria                            acyltransferase 3 [Mycobacterium sp. KMS].  
119870070         10TMAct                                                                                    Mkms_4040                387    Mycobacterium sp. KMS                                                 actinobacteria                            acyltransferase 3 [Mycobacterium sp. KMS].  
119870447         SIG+10TMAct+TM+GDSL                                                                        Mkms_4418                681    Mycobacterium sp. KMS                                                 actinobacteria                            acyltransferase 3 [Mycobacterium sp. KMS].  
119855062         10TMAct+TM+GDSL                                                                            Mkms_5673                728    Mycobacterium sp. KMS                                                 actinobacteria                            acyltransferase 3 [Mycobacterium sp. KMS].  
119866196         10TMAct+TM+GDSL                                                                            Mkms_0140                726    Mycobacterium sp. KMS                                                 actinobacteria                            acyltransferase 3 [Mycobacterium sp. KMS].  
119867025         10TMAct+TM                                                                                 Mkms_0973                402    Mycobacterium sp. KMS                                                 actinobacteria                            acyltransferase 3 [Mycobacterium sp. KMS].  
119869171         10TMAct+TM+GDSL                                                                            Mkms_3139                716    Mycobacterium sp. KMS                                                 actinobacteria                            acyltransferase 3 [Mycobacterium sp. KMS].  
119867289         SIG+10TMAct                                                                                Mkms_1239                396    Mycobacterium sp. KMS                                                 actinobacteria                            acyltransferase 3 [Mycobacterium sp. KMS].  
119867007         10TMAct+TM+GDSL                                                                            Mkms_0955                710    Mycobacterium sp. KMS                                                 actinobacteria                            acyltransferase 3 [Mycobacterium sp. KMS].  
108797202         10TMAct                                                                                    Mmcs_0222                392    Mycobacterium sp. MCS                                                 actinobacteria                            acyltransferase 3 [Mycobacterium sp. MCS].  
108800930         10TMAct                                                                                    Mmcs_3966                387    Mycobacterium sp. MCS                                                 actinobacteria                            acyltransferase 3 [Mycobacterium sp. MCS].  
108798193         SIG+10TMAct                                                                                Mmcs_1222                396    Mycobacterium sp. MCS                                                 actinobacteria                            acyltransferase 3 [Mycobacterium sp. MCS].  
108801296         SIG+10TMAct+TM+GDSL                                                                        Mmcs_4332                681    Mycobacterium sp. MCS                                                 actinobacteria                            acyltransferase 3 [Mycobacterium sp. MCS].  
108797929         10TMAct+TM                                                                                 Mmcs_0955                402    Mycobacterium sp. MCS                                                 actinobacteria                            acyltransferase 3 [Mycobacterium sp. MCS].  
108797111         10TMAct+TM+GDSL                                                                            Mmcs_0131                726    Mycobacterium sp. MCS                                                 actinobacteria                            acyltransferase 3 [Mycobacterium sp. MCS].  
108797912         10TMAct+TM+GDSL                                                                            Mmcs_0938                710    Mycobacterium sp. MCS                                                 actinobacteria                            acyltransferase 3 [Mycobacterium sp. MCS].  
108800045         10TMAct+TM+GDSL                                                                            Mmcs_3079                716    Mycobacterium sp. MCS                                                 actinobacteria                            acyltransferase 3 [Mycobacterium sp. MCS].  
15840700          SIG+10TMAct                                                                                MT1293                   383    Mycobacterium tuberculosis CDC1551                                    actinobacteria                            acyltransferase, putative [Mycobacterium tuberculosis CDC1551].  
15839608          10TMAct                                                                                    MT0238                   379    Mycobacterium tuberculosis CDC1551                                    actinobacteria                            acyltransferase [Mycobacterium tuberculosis CDC1551].  
15839492          SIG+10TMAct+TM+GDSL                                                                        MT0120                   685    Mycobacterium tuberculosis CDC1551                                    actinobacteria                            acetyltransferase, putative [Mycobacterium tuberculosis CDC1551].  
148822788         10TMAct+TM+GDSL                                                                            TBFG_11597               729    Mycobacterium tuberculosis F11                                        actinobacteria                            hypothetical protein TBFG_11597 [Mycobacterium tuberculosis F11].  
148659874         SIG+10TMAct+TM+GDSL                                                                        MRA_0117                 685    Mycobacterium tuberculosis H37Ra                                      actinobacteria                            putative transmembrane acyltransferase [Mycobacterium tuberculosis  
15607369          10TMAct                                                                                    Rv0228                   407    Mycobacterium tuberculosis H37Rv                                      actinobacteria                            integral membrane acyltransferase [Mycobacterium tuberculosis  
118619860         10TMAct+TM+GDSL                                                                            MUL_4810                 670    Mycobacterium ulcerans Agy99                                          actinobacteria                            transmembrane acyltransferase [Mycobacterium ulcerans Agy99].  
118616420         SIG+10TMAct+TM+GDSL                                                                        MUL_0609                 681    Mycobacterium ulcerans Agy99                                          actinobacteria                            transmembrane acyltransferase [Mycobacterium ulcerans Agy99].  
118617206         SIG+10TMAct+TM+GDSL                                                                        MUL_1556                 721    Mycobacterium ulcerans Agy99                                          actinobacteria                            hypothetical protein MUL_1556 [Mycobacterium ulcerans Agy99].  
118619599         10TMAct                                                                                    MUL_4492                 380    Mycobacterium ulcerans Agy99                                          actinobacteria                            integral membrane acyltransferase [Mycobacterium ulcerans Agy99].  
118616849         10TMAct                                                                                    MUL_1127                 402    Mycobacterium ulcerans Agy99                                          actinobacteria                            integral membrane acyltransferase [Mycobacterium ulcerans Agy99].  
120405239         SIG+10TMAct                                                                                Mvan_4285                421    Mycobacterium vanbaalenii PYR-1                                       actinobacteria                            acyltransferase 3 [Mycobacterium vanbaalenii PYR-1].  
120405825         SIG+10TMAct+TM+GDSL                                                                        Mvan_4875                671    Mycobacterium vanbaalenii PYR-1                                       actinobacteria                            acyltransferase 3 [Mycobacterium vanbaalenii PYR-1].  
120405421         10TMAct                                                                                    Mvan_4469                387    Mycobacterium vanbaalenii PYR-1                                       actinobacteria                            acyltransferase 3 [Mycobacterium vanbaalenii PYR-1].  
120401277         10TMAct                                                                                    Mvan_0251                394    Mycobacterium vanbaalenii PYR-1                                       actinobacteria                            acyltransferase 3 [Mycobacterium vanbaalenii PYR-1].  
120403771         SIG+10TMAct+TM+GDSL                                                                        Mvan_2787                731    Mycobacterium vanbaalenii PYR-1                                       actinobacteria                            acyltransferase 3 [Mycobacterium vanbaalenii PYR-1].  
120404663         10TMAct+TM+GDSL                                                                            Mvan_3701                728    Mycobacterium vanbaalenii PYR-1                                       actinobacteria                            acyltransferase 3 [Mycobacterium vanbaalenii PYR-1].  
120402676         TM+10TMAct+TM                                                                              Mvan_1670                438    Mycobacterium vanbaalenii PYR-1                                       actinobacteria                            acyltransferase 3 [Mycobacterium vanbaalenii PYR-1].  
54026897          10TMAct+TM+GDSL                                                                            nfa49230                 693    Nocardia farcinica IFM 10152                                          actinobacteria                            putative acyltransferase [Nocardia farcinica IFM 10152].  
54027445          SIG+10TMAct                                                                                nfa54710                 398    Nocardia farcinica IFM 10152                                          actinobacteria                            putative acyltransferase [Nocardia farcinica IFM 10152].  
119717221         10TMAct                                                                                    Noca_2997                409    Nocardioides sp. JS614                                                actinobacteria                            acyltransferase 3 [Nocardioides sp. JS614].  
163840511         10TMAct+TM+GDSL                                                                            RSal33209_1767           687    Renibacterium salmoninarum ATCC 33209                                 actinobacteria                            putative acyltransferase [Renibacterium salmoninarum ATCC 33209].  
163840359         10TMAct+TM+GDSL                                                                            RSal33209_1614           736    Renibacterium salmoninarum ATCC 33209                                 actinobacteria                            acyltransferase domain-containing protein [Renibacterium  
111022158         SIG+10TMAct                                                                                RHA1_ro05191             399    Rhodococcus jostii RHA1                                               actinobacteria                            acyltransferase [Rhodococcus jostii RHA1].  
111017883         10TMAct+GDSL                                                                               RHA1_ro00864             728    Rhodococcus jostii RHA1                                               actinobacteria                            acyltransferase [Rhodococcus jostii RHA1].  
111019532         SIG+10TMAct+GDSL                                                                           RHA1_ro02541             687    Rhodococcus jostii RHA1                                               actinobacteria                            acyltransferase [Rhodococcus jostii RHA1].  
108804886         10TMAct+TM+GDSL                                                                            Rxyl_2066                656    Rubrobacter xylanophilus DSM 9941                                     actinobacteria                            acyltransferase 3 [Rubrobacter xylanophilus DSM 9941].  
159035885         SIG+10TMAct                                                                                Sare_0212                403    Salinispora arenicola CNS-205                                         actinobacteria                            acyltransferase 3 [Salinispora arenicola CNS-205].  
145592760         SIG+10TMAct                                                                                Strop_0194               403    Salinispora tropica CNB-440                                           actinobacteria                            acyltransferase 3 [Salinispora tropica CNB-440].  
21225796          SIG+10TMAct+TM                                                                             SCO7531                  418    Streptomyces coelicolor A3(2)                                         actinobacteria                            integral membrane protein [Streptomyces coelicolor A3(2)].  
182438557         10TMAct+TM+GDSL                                                                            SGR_4764                 1029   Streptomyces griseus subsp. griseus NBRC 13350                        actinobacteria                            acyltransferase [Streptomyces griseus subsp. griseus NBRC 13350].  
72162857          SIG+10TMAct                                                                                Tfu_2458                 424    Thermobifida fusca YX                                                 actinobacteria                            putative acyltransferase [Thermobifida fusca YX].  
72162102          SIG+10TMAct+TM+GDSL                                                                        Tfu_1701                 690    Thermobifida fusca YX                                                 actinobacteria                            hypothetical protein Tfu_1701 [Thermobifida fusca YX].  
188997166         10TMAct+TM+GDSL                                                                            SYO3AOP1_1252            673    Sulfurihydrogenibium sp. YO3AOP1                                      aquificae                                 acyltransferase 3 [Sulfurihydrogenibium sp. YO3AOP1].  
188997316         10TMAct+TM+GDSL                                                                            SYO3AOP1_1409            695    Sulfurihydrogenibium sp. YO3AOP1                                      aquificae                                 acyltransferase 3 [Sulfurihydrogenibium sp. YO3AOP1].  
110637312         SIG+10TMAct                                                                                CHU_0898                 367    Cytophaga hutchinsonii ATCC 33406                                     bacteroidetes/chlorobi                    acyltransferase family protein [Cytophaga hutchinsonii ATCC 33406].  
194337575         SIG+10TMAct                                                                                Ppha_2590                360    Pelodictyon phaeoclathratiforme BU-1                                  bacteroidetes/chlorobi                    acyltransferase 3 [Pelodictyon phaeoclathratiforme BU-1].  
110638582         SIG+10TMAct                                                                                CHU_2186                 369    Cytophaga hutchinsonii ATCC 33406                                     bacteroidetes/chlorobi                    acyltransferase [Cytophaga hutchinsonii ATCC 33406].  
119358374         10TMAct+TM+GDSL                                                                            Cpha266_2606             603    Chlorobium phaeobacteroides DSM 266                                   bacteroidetes/chlorobi                    acyltransferase 3 [Chlorobium phaeobacteroides DSM 266].  
150024854         10TMAct                                                                                    FP0761                   377    Flavobacterium psychrophilum JIP02/86                                 bacteroidetes/chlorobi                    acyltransferase [Flavobacterium psychrophilum JIP02/86].  
78189231          10TMAct+TM+GDSL                                                                            Cag_1265                 660    Chlorobium chlorochromatii CaD3                                       bacteroidetes/chlorobi                    putative membrane-located cell surface saccharide saccharide  
146298754         10TMAct                                                                                    Fjoh_0993                371    Flavobacterium johnsoniae UW101                                       bacteroidetes/chlorobi                    acyltransferase 3 [Flavobacterium johnsoniae UW101].  
182414569         SIG+10TMAct                                                                                Oter_2754                362    Opitutus terrae PB90-1                                                chlamydiae/verrucomicrobia                acyltransferase 3 [Opitutus terrae PB90-1].  
182414321         10TMAct+TM+GDSL                                                                            Oter_2505                665    Opitutus terrae PB90-1                                                chlamydiae/verrucomicrobia                acyltransferase 3 [Opitutus terrae PB90-1].  
187735266         10TMAct+GDSL                                                                               Amuc_0761                690    Akkermansia muciniphila ATCC BAA-835                                  chlamydiae/verrucomicrobia                acyltransferase 3 [Akkermansia muciniphila ATCC BAA-835].  
186686201         SIG+10TMAct                                                                                Npun_R6168               339    Nostoc punctiforme PCC 73102                                          cyanobacteria                             acyltransferase 3 [Nostoc punctiforme PCC 73102].  
159902581         10TMAct+TM+GDSL                                                                            P9211_00401              696    Prochlorococcus marinus str. MIT 9211                                 cyanobacteria                             acyltransferase [Prochlorococcus marinus str. MIT 9211].  
123965276         10TMAct+TM+GDSL                                                                            P9515_00411              622    Prochlorococcus marinus str. MIT 9515                                 cyanobacteria                             hypothetical protein P9515_00411 [Prochlorococcus marinus str. MIT  
33240711          10TMAct+TM+GDSL                                                                            Pro1262                  696    Prochlorococcus marinus subsp. marinus str. CCMP1375                  cyanobacteria                             membrane associated acyltransferase [Prochlorococcus marinus subsp.  
33240985          10TMAct+TM+GDSL                                                                            Pro1536                  704    Prochlorococcus marinus subsp. marinus str. CCMP1375                  cyanobacteria                             membrane associated acyltransferase [Prochlorococcus marinus subsp.  
33861682          10TMAct+TM+GDSL                                                                            PMM1126                  671    Prochlorococcus marinus subsp. pastoris str. CCMP1986                 cyanobacteria                             hypothetical protein PMM1126 [Prochlorococcus marinus subsp.  
113954871         10TMAct+GDSL                                                                               sync_2921                707    Synechococcus sp. CC9311                                              cyanobacteria                             putative acyltransferase [Synechococcus sp. CC9311].  
148241244         10TMAct+GDSL                                                                               SynRCC307_0145           694    Synechococcus sp. RCC307                                              cyanobacteria                             membrane associated acyltransferase [Synechococcus sp. RCC307].  
116624308         SIG+10TMAct                                                                                Acid_5226                422    Solibacter usitatus Ellin6076                                         fibrobacteres/acidobacteria               acyltransferase 3 [Solibacter usitatus Ellin6076].  
116621370         SIG+10TMAct                                                                                Acid_2251                387    Solibacter usitatus Ellin6076                                         fibrobacteres/acidobacteria               acyltransferase 3 [Solibacter usitatus Ellin6076].  
94969765          SIG+10TMAct                                                                                Acid345_2738             362    Acidobacteria bacterium Ellin345                                      fibrobacteres/acidobacteria               acyltransferase 3 [Acidobacteria bacterium Ellin345].  
94967101          10TMAct                                                                                    Acid345_0070             392    Acidobacteria bacterium Ellin345                                      fibrobacteres/acidobacteria               acyltransferase 3 [Acidobacteria bacterium Ellin345].  
94968810          SIG+10TMAct                                                                                Acid345_1783             346    Acidobacteria bacterium Ellin345                                      fibrobacteres/acidobacteria               acyltransferase 3 [Acidobacteria bacterium Ellin345].  
116620311         10TMAct+TM+GDSL                                                                            Acid_1188                694    Solibacter usitatus Ellin6076                                         fibrobacteres/acidobacteria               acyltransferase 3 [Solibacter usitatus Ellin6076].  
154685117         10TMAct+TM+GDSL                                                                            yrhL                     643    Bacillus amyloliquefaciens FZB42                                      firmicutes                                YrhL [Bacillus amyloliquefaciens FZB42].  
52079038          10TMAct+TM+GDSL                                                                            yrhL                     626    Bacillus licheniformis ATCC 14580                                     firmicutes                                ABC transporter [Bacillus licheniformis ATCC 14580].  
157694332         10TMAct+TM+GDSL                                                                            yrhL                     607    Bacillus pumilus SAFR-032                                             firmicutes                                acyltransferase [Bacillus pumilus SAFR-032].  
16079768          10TMAct+TM+GDSL                                                                            yrhL                     634    Bacillus subtilis subsp. subtilis str. 168                            firmicutes                                hypothetical protein BSU27140 [Bacillus subtilis subsp. subtilis  
163943246         10TMAct+TM+GDSL                                                                            BcerKBAB4_5518           604    Bacillus weihenstephanensis KBAB4                                     firmicutes                                acyltransferase 3 [Bacillus weihenstephanensis KBAB4].  
170759263         10TMAct+TM+GDSL                                                                            CLK_0257                 586    Clostridium botulinum A3 str. Loch Maree                              firmicutes                                O-acetyltransferase OatA [Clostridium botulinum A3 str. Loch  
89893851          SIG+10TMAct+TM+GDSL                                                                        DSY1105                  625    Desulfitobacterium hafniense Y51                                      firmicutes                                hypothetical protein DSY1105 [Desulfitobacterium hafniense Y51].  
29375374          10TMAct+TM+GDSL                                                                            EF0783                   625    Enterococcus faecalis V583                                            firmicutes                                acyltransferase, putative [Enterococcus faecalis V583].  
172056333         SIG+10TMAct+TM+GDSL                                                                        Exig_0290                596    Exiguobacterium sibiricum 255-15                                      firmicutes                                acyltransferase 3 [Exiguobacterium sibiricum 255-15].  
172058764         10TMAct+TM+GDSL                                                                            Exig_2760                677    Exiguobacterium sibiricum 255-15                                      firmicutes                                acyltransferase 3 [Exiguobacterium sibiricum 255-15].  
58337246          10TMAct+TM+GDSL                                                                            LBA0948                  597    Lactobacillus acidophilus NCFM                                        firmicutes                                putative inner membrane trans-acylase protein [Lactobacillus  
116332949         10TMAct+TM+GDSL                                                                            LVIS_0279                646    Lactobacillus brevis ATCC 367                                         firmicutes                                acyltransferase [Lactobacillus brevis ATCC 367].  
116494838         SIG+10TMAct+TM+GDSL                                                                        LSEI_1351                664    Lactobacillus casei ATCC 334                                          firmicutes                                acyltransferase [Lactobacillus casei ATCC 334].  
191638346         10TMAct+TM+GDSL                                                                            LCABL_15730              659    Lactobacillus casei BL23                                              firmicutes                                Acyltransferase 3 [Lactobacillus casei BL23].  
116513894         10TMAct+TM+GDSL                                                                            LBUL_0748                647    Lactobacillus delbrueckii subsp. bulgaricus ATCC BAA-365              firmicutes                                acyltransferase [Lactobacillus delbrueckii subsp. bulgaricus ATCC  
116629528         10TMAct+TM+GDSL                                                                            LGAS_0873                646    Lactobacillus gasseri ATCC 33323                                      firmicutes                                acyltransferase [Lactobacillus gasseri ATCC 33323].  
161507440         SIG+10TMAct+TM+GDSL                                                                        lhv_1042                 641    Lactobacillus helveticus DPC 4571                                     firmicutes                                acyltransferase family protein [Lactobacillus helveticus DPC 4571].  
42518997          10TMAct+TM+GDSL                                                                            LJ1072                   641    Lactobacillus johnsonii NCC 533                                       firmicutes                                hypothetical protein LJ1072 [Lactobacillus johnsonii NCC 533].  
28377697          10TMAct+TM+GDSL                                                                            lp_0856                  660    Lactobacillus plantarum WCFS1                                         firmicutes                                acyltransferase (putative) [Lactobacillus plantarum WCFS1].  
28377753          SIG+10TMAct+TM+GDSL                                                                        lp_0925                  615    Lactobacillus plantarum WCFS1                                         firmicutes                                acyltransferase [Lactobacillus plantarum WCFS1].  
184153062         10TMAct+TM+GDSL                                                                            LAR_0407                 621    Lactobacillus reuteri JCM 1112                                        firmicutes                                putative acyltransferase [Lactobacillus reuteri JCM 1112].  
81428655          10TMAct+TM+GDSL                                                                            LSA1044                  642    Lactobacillus sakei subsp. sakei 23K                                  firmicutes                                putative acyltransferase [Lactobacillus sakei subsp. sakei 23K].  
81428261          SIG+10TMAct+TM+GDSL                                                                        LSA0646                  605    Lactobacillus sakei subsp. sakei 23K                                  firmicutes                                acyltransferase family 3 protein [Lactobacillus sakei subsp. sakei  
90962071          SIG+10TMAct+TM+GDSL                                                                        LSL_1096                 617    Lactobacillus salivarius UCC118                                       firmicutes                                acyltransferase [Lactobacillus salivarius UCC118].  
125625151         SIG+10TMAct+TM+GDSL                                                                        llmg_2391                605    Lactococcus lactis subsp. cremoris MG1363                             firmicutes                                hypothetical protein llmg_2391 [Lactococcus lactis subsp. cremoris  
116513061         SIG+10TMAct+TM+GDSL                                                                        LACR_2409                605    Lactococcus lactis subsp. cremoris SK11                               firmicutes                                hypothetical protein LACR_2409 [Lactococcus lactis subsp. cremoris  
15674089          SIG+10TMAct+TM+GDSL                                                                        yvhB                     605    Lactococcus lactis subsp. lactis Il1403                               firmicutes                                hypothetical protein L158566 [Lactococcus lactis subsp. lactis  
170016816         SIG+10TMAct+TM+GDSL                                                                        LCK_00458                618    Leuconostoc citreum KM20                                              firmicutes                                putative acyltransferase [Leuconostoc citreum KM20].  
116617661         SIG+10TMAct+TM+GDSL                                                                        LEUM_0545                621    Leuconostoc mesenteroides subsp. mesenteroides ATCC 8293              firmicutes                                acyltransferase [Leuconostoc mesenteroides subsp. mesenteroides  
16800397          SIG+10TMAct+TM+GDSL                                                                        lin1329                  633    Listeria innocua Clip11262                                            firmicutes                                hypothetical protein lin1329 [Listeria innocua Clip11262].  
16803331          SIG+10TMAct+TM+GDSL                                                                        lmo1291                  622    Listeria monocytogenes EGD-e                                          firmicutes                                hypothetical protein lmo1291 [Listeria monocytogenes EGD-e].  
46907517          SIG+10TMAct+TM+GDSL                                                                        LMOf2365_1308            628    Listeria monocytogenes str. 4b F2365                                  firmicutes                                acyltransferase family protein [Listeria monocytogenes str. 4b  
116872722         SIG+10TMAct+TM+GDSL                                                                        lwe1306                  603    Listeria welshimeri serovar 6b str. SLCC5334                          firmicutes                                acyltransferase family protein [Listeria welshimeri serovar 6b str.  
169829284         10TMAct+TM+GDSL                                                                            Bsph_3834                627    Lysinibacillus sphaericus C3-41                                       firmicutes                                O-acetyltransferase oatA [Lysinibacillus sphaericus C3-41].  
116493468         10TMAct+TM+GDSL                                                                            PEPE_1740                644    Pediococcus pentosaceus ATCC 25745                                    firmicutes                                acyltransferase [Pediococcus pentosaceus ATCC 25745].  
82752149          10TMAct+TM+GDSL                                                                            SAB2441c                 603    Staphylococcus aureus RF122                                           firmicutes                                membrane-embedded acyltransferase [Staphylococcus aureus RF122].  
148268999         10TMAct+TM+GDSL                                                                            SaurJH9_2589             603    Staphylococcus aureus subsp. aureus JH9                               firmicutes                                acyltransferase 3 [Staphylococcus aureus subsp. aureus JH9].  
49484767          10TMAct+TM+GDSL                                                                            SAR2649                  603    Staphylococcus aureus subsp. aureus MRSA252                           firmicutes                                hypothetical protein SAR2649 [Staphylococcus aureus subsp. aureus  
49483135          10TMAct+TM+GDSL                                                                            SAR0937                  604    Staphylococcus aureus subsp. aureus MRSA252                           firmicutes                                hypothetical protein SAR0937 [Staphylococcus aureus subsp. aureus  
156979300         10TMAct+TM+GDSL                                                                            SAHV_0969                604    Staphylococcus aureus subsp. aureus Mu3                               firmicutes                                hypothetical protein SAHV_0969 [Staphylococcus aureus subsp. aureus  
88194668          10TMAct+TM+GDSL                                                                            SAOUHSC_00911            604    Staphylococcus aureus subsp. aureus NCTC 8325                         firmicutes                                hypothetical protein SAOUHSC_00911 [Staphylococcus aureus subsp.  
27467591          10TMAct+TM+GDSL                                                                            SE0673                   607    Staphylococcus epidermidis ATCC 12228                                 firmicutes                                lipopolysaccharide modification acyltransferase [Staphylococcus  
27469043          10TMAct+TM+GDSL                                                                            SE2125                   602    Staphylococcus epidermidis ATCC 12228                                 firmicutes                                acyltransferase [Staphylococcus epidermidis ATCC 12228].  
57865591          10TMAct+TM+GDSL                                                                            SERP2137                 602    Staphylococcus epidermidis RP62A                                      firmicutes                                hypothetical protein SERP2137 [Staphylococcus epidermidis RP62A].  
70726977          10TMAct+TM+GDSL                                                                            SH1976                   608    Staphylococcus haemolyticus JCSC1435                                  firmicutes                                hypothetical protein SH1976 [Staphylococcus haemolyticus JCSC1435].  
73663110          10TMAct+TM+GDSL                                                                            SSP1801                  610    Staphylococcus saprophyticus subsp. saprophyticus ATCC 15305          firmicutes                                putative acyltransferase [Staphylococcus saprophyticus subsp.  
22536237          SIG+10TMAct+TM+GDSL                                                                        SAG0052                  592    Streptococcus agalactiae 2603V/R                                      firmicutes                                hypothetical protein SAG0052 [Streptococcus agalactiae 2603V/R].  
76787884          SIG+10TMAct+TM+GDSL                                                                        SAK_0085                 592    Streptococcus agalactiae A909                                         firmicutes                                hypothetical protein SAK_0085 [Streptococcus agalactiae A909].  
25010127          SIG+10TMAct+TM+GDSL                                                                        gbs0052                  592    Streptococcus agalactiae NEM316                                       firmicutes                                hypothetical protein gbs0052 [Streptococcus agalactiae NEM316].  
157151084         SIG+10TMAct+TM+GDSL                                                                        SGO_0112                 604    Streptococcus gordonii str. Challis substr. CH1                       firmicutes                                putative acyltransferase [Streptococcus gordonii str. Challis  
24378593          SIG+10TMAct+TM+GDSL                                                                        SMU.67                   596    Streptococcus mutans UA159                                            firmicutes                                putative acyltransferase [Streptococcus mutans UA159].  
182684994         SIG+10TMAct+TM+GDSL                                                                        SPCG_2024                605    Streptococcus pneumoniae CGSP14                                       firmicutes                                hypothetical protein SPCG_2024 [Streptococcus pneumoniae CGSP14].  
116516353         SIG+10TMAct+TM+GDSL                                                                        SPD_1867                 605    Streptococcus pneumoniae D39                                          firmicutes                                hypothetical protein SPD_1867 [Streptococcus pneumoniae D39].  
194396662         SIG+10TMAct+TM+GDSL                                                                        SPG_1972                 605    Streptococcus pneumoniae G54                                          firmicutes                                putative acyltransferase [Streptococcus pneumoniae G54].  
169833229         SIG+10TMAct+TM+GDSL                                                                        SPH_2212                 605    Streptococcus pneumoniae Hungary19A-6                                 firmicutes                                acyltransferase [Streptococcus pneumoniae Hungary19A-6].  
94989549          SIG+10TMAct+TM+GDSL                                                                        MGAS10270_Spy0040        473    Streptococcus pyogenes MGAS10270                                      firmicutes                                acyltransferase [Streptococcus pyogenes MGAS10270].  
50913433          SIG+10TMAct+TM+GDSL                                                                        M6_Spy0087               591    Streptococcus pyogenes MGAS10394                                      firmicutes                                acyltransferase [Streptococcus pyogenes MGAS10394].  
94993436          SIG+10TMAct+TM+GDSL                                                                        MGAS10750_Spy0040        591    Streptococcus pyogenes MGAS10750                                      firmicutes                                Acyltransferase family [Streptococcus pyogenes MGAS10750].  
71909852          SIG+10TMAct+TM+GDSL                                                                        M5005_Spy_0038           591    Streptococcus pyogenes MGAS5005                                       firmicutes                                acyltransferase [Streptococcus pyogenes MGAS5005].  
71902704          SIG+10TMAct+TM+GDSL                                                                        M28_Spy0038              591    Streptococcus pyogenes MGAS6180                                       firmicutes                                acyltransferase [Streptococcus pyogenes MGAS6180].  
19745236          SIG+10TMAct+TM+GDSL                                                                        spyM18_0042              591    Streptococcus pyogenes MGAS8232                                       firmicutes                                putative acyltransferase [Streptococcus pyogenes MGAS8232].  
94987670          SIG+10TMAct+TM+GDSL                                                                        MGAS9429_Spy0039         591    Streptococcus pyogenes MGAS9429                                       firmicutes                                acyltransferase [Streptococcus pyogenes MGAS9429].  
209558622         SIG+10TMAct+TM+GDSL                                                                        Spy49_0037               591    Streptococcus pyogenes NZ131                                          firmicutes                                Putative acyltransferase [Streptococcus pyogenes NZ131].  
28894948          SIG+10TMAct+TM+GDSL                                                                        SPs0036                  591    Streptococcus pyogenes SSI-1                                          firmicutes                                putative acyltransferase [Streptococcus pyogenes SSI-1].  
139472922         SIG+10TMAct+TM+GDSL                                                                        SpyM50038                591    Streptococcus pyogenes str. Manfredo                                  firmicutes                                acyltransferase family protein [Streptococcus pyogenes str.  
125716948         SIG+10TMAct+TM+GDSL                                                                        SSA_0067                 601    Streptococcus sanguinis SK36                                          firmicutes                                acyltransferase [Streptococcus sanguinis SK36].  
146319347         SIG+10TMAct+TM+GDSL                                                                        SSU05_1693               599    Streptococcus suis 05ZYH33                                            firmicutes                                acyltransferase [Streptococcus suis 05ZYH33].  
146321551         SIG+10TMAct+TM+TM                                                                          SSU98_1704               469    Streptococcus suis 98HAH33                                            firmicutes                                acyltransferase [Streptococcus suis 98HAH33].  
55823824          SIG+10TMAct+TM+GDSL                                                                        str1937                  607    Streptococcus thermophilus CNRZ1066                                   firmicutes                                acyltransferase [Streptococcus thermophilus CNRZ1066].  
55821908          SIG+10TMAct+TM+GDSL                                                                        stu1937                  607    Streptococcus thermophilus LMG 18311                                  firmicutes                                hypothetical protein stu1937 [Streptococcus thermophilus LMG  
19705320          10TMAct+TM+TM+GDSL                                                                         FN2029                   604    Fusobacterium nucleatum subsp. nucleatum ATCC 25586                   fusobacteria                              O-antigen acetylase [Fusobacterium nucleatum subsp. nucleatum ATCC  
117924439         SIG+10TMAct+TM+GDSL                                                                        Mmc1_1138                629    Magnetococcus sp. MC-1                                                proteobacteria                            acyltransferase 3 [Magnetococcus sp. MC-1].  
158425140         10TMAct+TM+GDSL                                                                            AZC_3516                 655    Azorhizobium caulinodans ORS 571                                      proteobacteria>alphaproteobacteria        putative O-antigen acetylase [Azorhizobium caulinodans ORS 571].  
158425628         10TMAct+TM+GDSL                                                                            AZC_4004                 633    Azorhizobium caulinodans ORS 571                                      proteobacteria>alphaproteobacteria        O-antigen acetylase [Azorhizobium caulinodans ORS 571].  
158425138         SIG+10TMAct+TM+GDSL                                                                        AZC_3514                 644    Azorhizobium caulinodans ORS 571                                      proteobacteria>alphaproteobacteria        putative O-antigen acetylase [Azorhizobium caulinodans ORS 571].  
182679087         10TMAct                                                                                    Bind_2124                350    Beijerinckia indica subsp. indica ATCC 9039                           proteobacteria>alphaproteobacteria        acyltransferase 3 [Beijerinckia indica subsp. indica ATCC 9039].  
27380051          10TMAct+TM+GDSL                                                                            bll4940                  651    Bradyrhizobium japonicum USDA 110                                     proteobacteria>alphaproteobacteria        putative putative membrane-located cell surface saccharide  
27377471          10TMAct                                                                                    blr2360                  393    Bradyrhizobium japonicum USDA 110                                     proteobacteria>alphaproteobacteria        hypothetical protein blr2360 [Bradyrhizobium japonicum USDA 110].  
148256448         10TMAct+TM+GDSL                                                                            BBta_5130                647    Bradyrhizobium sp. BTAi1                                              proteobacteria>alphaproteobacteria        putative acyltransferase 3 [Bradyrhizobium sp. BTAi1].  
148256587         10TMAct+TM+GDSL                                                                            BBta_5281                644    Bradyrhizobium sp. BTAi1                                              proteobacteria>alphaproteobacteria        hypothetical protein BBta_5281 [Bradyrhizobium sp. BTAi1].  
148255295         10TMAct+GDSL                                                                               BBta_3904                678    Bradyrhizobium sp. BTAi1                                              proteobacteria>alphaproteobacteria        putative acyltransferase [Bradyrhizobium sp. BTAi1].  
148256924         10TMAct+GDSL                                                                               BBta_5645                634    Bradyrhizobium sp. BTAi1                                              proteobacteria>alphaproteobacteria        putative acyltransferase, group 3 [Bradyrhizobium sp. BTAi1].  
148256450         10TMAct+TM+GDSL                                                                            BBta_5132                638    Bradyrhizobium sp. BTAi1                                              proteobacteria>alphaproteobacteria        putative acyltransferase [Bradyrhizobium sp. BTAi1].  
148256926         10TMAct+GDSL                                                                               BBta_5647                654    Bradyrhizobium sp. BTAi1                                              proteobacteria>alphaproteobacteria        putative acyltransferase, group 3 [Bradyrhizobium sp. BTAi1].  
148256449         SIG+10TMAct+TM+GDSL                                                                        BBta_5131                632    Bradyrhizobium sp. BTAi1                                              proteobacteria>alphaproteobacteria        putative acyltransferase [Bradyrhizobium sp. BTAi1].  
146342020         10TMAct+TM+GDSL                                                                            BRADO5157                677    Bradyrhizobium sp. ORS278                                             proteobacteria>alphaproteobacteria        putative acyltransferase, group 3; putative O-antigen acetylase  
146342022         10TMAct+GDSL                                                                               BRADO5159                684    Bradyrhizobium sp. ORS278                                             proteobacteria>alphaproteobacteria        putative acyltransferase, group 3; putative O-antigen acetylase  
146341713         SIG+10TMAct                                                                                BRADO4818                418    Bradyrhizobium sp. ORS278                                             proteobacteria>alphaproteobacteria        putative acyltransferase; putative membrane protein [Bradyrhizobium  
146339895         10TMAct+TM+GDSL                                                                            BRADO2897                644    Bradyrhizobium sp. ORS278                                             proteobacteria>alphaproteobacteria        hypothetical protein BRADO2897 [Bradyrhizobium sp. ORS278].  
146340004         10TMAct+GDSL                                                                               BRADO3009                632    Bradyrhizobium sp. ORS278                                             proteobacteria>alphaproteobacteria        putative acyltransferase [Bradyrhizobium sp. ORS278].  
85375718          10TMAct+TM+GDSL                                                                            ELI_14455                638    Erythrobacter litoralis HTCC2594                                      proteobacteria>alphaproteobacteria        putative membrane-located cell surface saccharide  
58040283          10TMAct                                                                                    GOX1853                  377    Gluconobacter oxydans 621H                                            proteobacteria>alphaproteobacteria        putative lipopolysaccharide modification acyltransferase  
114327406         10TMAct                                                                                    GbCGDNIH1_0742           369    Granulibacter bethesdensis CGDNIH1                                    proteobacteria>alphaproteobacteria        acyltransferase [Granulibacter bethesdensis CGDNIH1].  
114800474         10TMAct+GDSL                                                                               HNE_2463                 658    Hyphomonas neptunium ATCC 15444                                       proteobacteria>alphaproteobacteria        acyltransferase family protein [Hyphomonas neptunium ATCC 15444].  
114798513         10TMAct+TM+GDSL                                                                            HNE_0039                 659    Hyphomonas neptunium ATCC 15444                                       proteobacteria>alphaproteobacteria        acyltransferase family protein [Hyphomonas neptunium ATCC 15444].  
13471978          10TMAct+TM+GDSL                                                                            mlr2124                  658    Mesorhizobium loti MAFF303099                                         proteobacteria>alphaproteobacteria        lipopolysaccharide modification acyltransferase [Mesorhizobium loti  
13471839          SIG+10TMAct                                                                                mlr1941                  388    Mesorhizobium loti MAFF303099                                         proteobacteria>alphaproteobacteria        hypothetical protein mlr1941 [Mesorhizobium loti MAFF303099].  
13474718          SIG+10TMAct+GDSL                                                                           mll5669                  628    Mesorhizobium loti MAFF303099                                         proteobacteria>alphaproteobacteria        O-antigen acetylase [Mesorhizobium loti MAFF303099].  
13470881          10TMAct+TM+GDSL                                                                            mlr0695                  688    Mesorhizobium loti MAFF303099                                         proteobacteria>alphaproteobacteria        O-antigen acetylase [Mesorhizobium loti MAFF303099].  
110632846         SIG+10TMAct+GDSL                                                                           Meso_0485                777    Mesorhizobium sp. BNC1                                                proteobacteria>alphaproteobacteria        acyltransferase 3 [Mesorhizobium sp. BNC1].  
170750984         SIG+10TMAct                                                                                Mrad2831_4595            358    Methylobacterium radiotolerans JCM 2831                               proteobacteria>alphaproteobacteria        acyltransferase 3 [Methylobacterium radiotolerans JCM 2831].  
170750305         10TMAct                                                                                    Mrad2831_3908            372    Methylobacterium radiotolerans JCM 2831                               proteobacteria>alphaproteobacteria        acyltransferase 3 [Methylobacterium radiotolerans JCM 2831].  
170742705         10TMAct                                                                                    M446_4586                385    Methylobacterium sp. 4-46                                             proteobacteria>alphaproteobacteria        acyltransferase 3 [Methylobacterium sp. 4-46].  
87199331          SIG+10TMAct+TM+GDSL                                                                        Saro_1310                621    Novosphingobium aromaticivorans DSM 12444                             proteobacteria>alphaproteobacteria        acyltransferase 3 [Novosphingobium aromaticivorans DSM 12444].  
87201239          10TMAct                                                                                    Saro_3227                368    Novosphingobium aromaticivorans DSM 12444                             proteobacteria>alphaproteobacteria        acyltransferase 3 [Novosphingobium aromaticivorans DSM 12444].  
209885012         10TMAct+GDSL                                                                               OCAR_5879                645    Oligotropha carboxidovorans OM5                                       proteobacteria>alphaproteobacteria        acyltransferase 3 [Oligotropha carboxidovorans OM5].  
154253741         SIG+10TMAct                                                                                Plav_3303                389    Parvibaculum lavamentivorans DS-1                                     proteobacteria>alphaproteobacteria        acyltransferase 3 [Parvibaculum lavamentivorans DS-1].  
154252357         10TMAct+TM+GDSL                                                                            Plav_1909                675    Parvibaculum lavamentivorans DS-1                                     proteobacteria>alphaproteobacteria        acyltransferase 3 [Parvibaculum lavamentivorans DS-1].  
154252394         10TMAct+TM+GDSL                                                                            Plav_1946                632    Parvibaculum lavamentivorans DS-1                                     proteobacteria>alphaproteobacteria        acyltransferase 3 [Parvibaculum lavamentivorans DS-1].  
154252395         SIG+10TMAct+TM+GDSL                                                                        Plav_1947                642    Parvibaculum lavamentivorans DS-1                                     proteobacteria>alphaproteobacteria        acyltransferase 3 [Parvibaculum lavamentivorans DS-1].  
154252870         10TMAct+GDSL                                                                               Plav_2428                637    Parvibaculum lavamentivorans DS-1                                     proteobacteria>alphaproteobacteria        acyltransferase 3 [Parvibaculum lavamentivorans DS-1].  
154252933         10TMAct                                                                                    Plav_2491                396    Parvibaculum lavamentivorans DS-1                                     proteobacteria>alphaproteobacteria        acyltransferase 3 [Parvibaculum lavamentivorans DS-1].  
86358781          10TMAct+TM+GDSL                                                                            RHE_CH03181              645    Rhizobium etli CFN 42                                                 proteobacteria>alphaproteobacteria        cell surface saccharide saccharide acetylase [Rhizobium etli CFN  
190893677         10TMAct+TM                                                                                 RHECIAT_CH0004112        393    Rhizobium etli CIAT 652                                               proteobacteria>alphaproteobacteria        putative acyltransferase protein [Rhizobium etli CIAT 652].  
190893007         10TMAct+GDSL                                                                               RHECIAT_CH0003424        645    Rhizobium etli CIAT 652                                               proteobacteria>alphaproteobacteria        putative membrane-located cell surface saccharide saccharide  
209548350         10TMAct                                                                                    Rleg2_0745               381    Rhizobium leguminosarum bv. trifolii WSM2304                          proteobacteria>alphaproteobacteria        acyltransferase 3 [Rhizobium leguminosarum bv. trifolii WSM2304].  
209548836         SIG+10TMAct+TM+GDSL                                                                        Rleg2_1233               656    Rhizobium leguminosarum bv. trifolii WSM2304                          proteobacteria>alphaproteobacteria        acyltransferase 3 [Rhizobium leguminosarum bv. trifolii WSM2304].  
77463243          SIG+10TMAct+TM+GDSL                                                                        RSP_2692                 647    Rhodobacter sphaeroides 2.4.1                                         proteobacteria>alphaproteobacteria        acyltransferase domain-containing protein [Rhodobacter sphaeroides  
146277909         SIG+10TMAct+GDSL                                                                           Rsph17025_1872           629    Rhodobacter sphaeroides ATCC 17025                                    proteobacteria>alphaproteobacteria        acyltransferase 3 [Rhodobacter sphaeroides ATCC 17025].  
126462117         SIG+10TMAct+GDSL                                                                           Rsph17029_1349           629    Rhodobacter sphaeroides ATCC 17029                                    proteobacteria>alphaproteobacteria        acyltransferase 3 [Rhodobacter sphaeroides ATCC 17029].  
115523159         10TMAct+GDSL                                                                               RPE_1138                 665    Rhodopseudomonas palustris BisA53                                     proteobacteria>alphaproteobacteria        hypothetical protein RPE_1138 [Rhodopseudomonas palustris BisA53].  
91974944          10TMAct+GDSL                                                                               RPD_0464                 660    Rhodopseudomonas palustris BisB5                                      proteobacteria>alphaproteobacteria        acyltransferase 3 [Rhodopseudomonas palustris BisB5].  
39933341          10TMAct+TM+GDSL                                                                            RPA0264                  645    Rhodopseudomonas palustris CGA009                                     proteobacteria>alphaproteobacteria        O-antigen acetylase [Rhodopseudomonas palustris CGA009].  
86747428          SIG+10TMAct+TM+GDSL                                                                        RPB_0302                 660    Rhodopseudomonas palustris HaA2                                       proteobacteria>alphaproteobacteria        acyltransferase 3 [Rhodopseudomonas palustris HaA2].  
192288696         10TMAct+TM+GDSL                                                                            Rpal_0265                645    Rhodopseudomonas palustris TIE-1                                      proteobacteria>alphaproteobacteria        acyltransferase 3 [Rhodopseudomonas palustris TIE-1].  
115345690         10TMAct+TM+GDSL                                                                            RD1_B0055                645    Roseobacter denitrificans OCh 114                                     proteobacteria>alphaproteobacteria        acyltransferase family protein [Roseobacter denitrificans OCh 114].  
110680561         SIG+10TMAct+TM+GDSL                                                                        RD1_3390                 655    Roseobacter denitrificans OCh 114                                     proteobacteria>alphaproteobacteria        acyltransferase, putative [Roseobacter denitrificans OCh 114].  
99077995          10TMAct                                                                                    TM1040_3862              374    Ruegeria sp. TM1040                                                   proteobacteria>alphaproteobacteria        acyltransferase 3 [Silicibacter sp. TM1040].  
150396975         SIG+10TMAct+GDSL                                                                           Smed_1772                683    Sinorhizobium medicae WSM419                                          proteobacteria>alphaproteobacteria        acyltransferase 3 [Sinorhizobium medicae WSM419].  
150377132         10TMAct+TM+GDSL                                                                            Smed_5014                695    Sinorhizobium medicae WSM419                                          proteobacteria>alphaproteobacteria        acyltransferase 3 [Sinorhizobium medicae WSM419].  
150376813         10TMAct                                                                                    Smed_4679                377    Sinorhizobium medicae WSM419                                          proteobacteria>alphaproteobacteria        acyltransferase 3 [Sinorhizobium medicae WSM419].  
150397183         10TMAct+GDSL                                                                               Smed_1981                671    Sinorhizobium medicae WSM419                                          proteobacteria>alphaproteobacteria        acyltransferase 3 [Sinorhizobium medicae WSM419].  
16264602          10TMAct                                                                                    SM_b21188                377    Sinorhizobium meliloti 1021                                           proteobacteria>alphaproteobacteria        acyltransferase [Sinorhizobium meliloti 1021].  
16264298          10TMAct+TM+GDSL                                                                            SM_b20810                677    Sinorhizobium meliloti 1021                                           proteobacteria>alphaproteobacteria        putative membrane-located cell surface saccharide saccharide  
16263003          10TMAct                                                                                    SMa1016                  424    Sinorhizobium meliloti 1021                                           proteobacteria>alphaproteobacteria        acyltransferase [Sinorhizobium meliloti 1021].  
148557509         10TMAct+TM+GDSL                                                                            Swit_4615                630    Sphingomonas wittichii RW1                                            proteobacteria>alphaproteobacteria        acyltransferase 3 [Sphingomonas wittichii RW1].  
148552969         SIG+10TMAct+TM+GDSL                                                                        Swit_0040                635    Sphingomonas wittichii RW1                                            proteobacteria>alphaproteobacteria        acyltransferase 3 [Sphingomonas wittichii RW1].  
154248368         10TMAct+TM+GDSL                                                                            Xaut_4448                679    Xanthobacter autotrophicus Py2                                        proteobacteria>alphaproteobacteria        acyltransferase 3 [Xanthobacter autotrophicus Py2].  
154248367         10TMAct+GDSL                                                                               Xaut_4447                695    Xanthobacter autotrophicus Py2                                        proteobacteria>alphaproteobacteria        acyltransferase 3 [Xanthobacter autotrophicus Py2].  
121594668         10TMAct                                                                                    Ajs_2326                 402    Acidovorax sp. JS42                                                   proteobacteria>betaproteobacteria         acyltransferase 3 [Acidovorax sp. JS42].  
56476270          10TMAct+TM+GDSL                                                                            ebA1519                  637    Aromatoleum aromaticum EbN1                                           proteobacteria>betaproteobacteria         acetylase [Aromatoleum aromaticum EbN1].  
119899544         10TMAct+GDSL                                                                               azo3255                  673    Azoarcus sp. BH72                                                     proteobacteria>betaproteobacteria         acyltransferase [Azoarcus sp. BH72].  
119899869         10TMAct+TM+GDSL                                                                            azo3580                  687    Azoarcus sp. BH72                                                     proteobacteria>betaproteobacteria         putative acyltransferase family protein [Azoarcus sp. BH72].  
115358119         SIG+10TMAct                                                                                Bamb_3369                372    Burkholderia ambifaria AMMD                                           proteobacteria>betaproteobacteria         acyltransferase 3 [Burkholderia ambifaria AMMD].  
115358110         10TMAct                                                                                    Bamb_3359                354    Burkholderia ambifaria AMMD                                           proteobacteria>betaproteobacteria         acyltransferase 3 [Burkholderia ambifaria AMMD].  
115358277         10TMAct                                                                                    Bamb_3527                408    Burkholderia ambifaria AMMD                                           proteobacteria>betaproteobacteria         acyltransferase 3 [Burkholderia ambifaria AMMD].  
172062903         SIG+10TMAct                                                                                BamMC406_3871            372    Burkholderia ambifaria MC40-6                                         proteobacteria>betaproteobacteria         acyltransferase 3 [Burkholderia ambifaria MC40-6].  
172063040         SIG+10TMAct                                                                                BamMC406_4010            383    Burkholderia ambifaria MC40-6                                         proteobacteria>betaproteobacteria         acyltransferase 3 [Burkholderia ambifaria MC40-6].  
172062896         10TMAct                                                                                    BamMC406_3864            354    Burkholderia ambifaria MC40-6                                         proteobacteria>betaproteobacteria         acyltransferase 3 [Burkholderia ambifaria MC40-6].  
107026740         SIG+10TMAct                                                                                Bcen_4396                326    Burkholderia cenocepacia AU 1054                                      proteobacteria>betaproteobacteria         acyltransferase 3 [Burkholderia cenocepacia AU 1054].  
107026598         SIG+10TMAct                                                                                Bcen_4250                383    Burkholderia cenocepacia AU 1054                                      proteobacteria>betaproteobacteria         acyltransferase 3 [Burkholderia cenocepacia AU 1054].  
116692075         SIG+10TMAct                                                                                Bcen2424_3978            372    Burkholderia cenocepacia HI2424                                       proteobacteria>betaproteobacteria         acyltransferase 3 [Burkholderia cenocepacia HI2424].  
116692068         10TMAct                                                                                    Bcen2424_3971            354    Burkholderia cenocepacia HI2424                                       proteobacteria>betaproteobacteria         acyltransferase 3 [Burkholderia cenocepacia HI2424].  
206563093         SIG+10TMAct                                                                                BCAM1241                 383    Burkholderia cenocepacia J2315                                        proteobacteria>betaproteobacteria         putative acyltransferase [Burkholderia cenocepacia J2315].  
206562866         SIG+10TMAct                                                                                BCAM1011                 372    Burkholderia cenocepacia J2315                                        proteobacteria>betaproteobacteria         putative acetyltransferase [Burkholderia cenocepacia J2315].  
206560877         SIG+10TMAct+TM+GDSL                                                                        BCAL2519                 669    Burkholderia cenocepacia J2315                                        proteobacteria>betaproteobacteria         putative O-antigen acetylase [Burkholderia cenocepacia J2315].  
206562860         10TMAct                                                                                    BCAM1005                 354    Burkholderia cenocepacia J2315                                        proteobacteria>betaproteobacteria         putative acyltransferase [Burkholderia cenocepacia J2315].  
206559568         SIG+10TMAct+TM+GDSL                                                                        BCAL1191                 669    Burkholderia cenocepacia J2315                                        proteobacteria>betaproteobacteria         putative O-antigen acetylase [Burkholderia cenocepacia J2315].  
170735933         SIG+10TMAct                                                                                Bcenmc03_3551            372    Burkholderia cenocepacia MC0-3                                        proteobacteria>betaproteobacteria         acyltransferase 3 [Burkholderia cenocepacia MC0-3].  
170735790         SIG+10TMAct                                                                                Bcenmc03_3407            383    Burkholderia cenocepacia MC0-3                                        proteobacteria>betaproteobacteria         acyltransferase 3 [Burkholderia cenocepacia MC0-3].  
170735940         10TMAct+TM                                                                                 Bcenmc03_3558            354    Burkholderia cenocepacia MC0-3                                        proteobacteria>betaproteobacteria         acyltransferase 3 [Burkholderia cenocepacia MC0-3].  
53717303          10TMAct+TM+GDSL                                                                            BMAA1498                 699    Burkholderia mallei ATCC 23344                                        proteobacteria>betaproteobacteria         putative O-antigen acetylase [Burkholderia mallei ATCC 23344].  
124382800         10TMAct+TM+GDSL                                                                            BMA10229_2108            727    Burkholderia mallei NCTC 10229                                        proteobacteria>betaproteobacteria         putative O-antigen acetylase [Burkholderia mallei NCTC 10229].  
126447542         SIG+10TMAct                                                                                BMA10247_A0541           352    Burkholderia mallei NCTC 10247                                        proteobacteria>betaproteobacteria         acyltransferase family protein [Burkholderia mallei NCTC 10247].  
126446020         SIG+10TMAct                                                                                BMA10247_A0550           375    Burkholderia mallei NCTC 10247                                        proteobacteria>betaproteobacteria         acyltransferase family protein [Burkholderia mallei NCTC 10247].  
121597250         SIG+TM+TM+10TMAct                                                                          BMASAVP1_1670            585    Burkholderia mallei SAVP1                                             proteobacteria>betaproteobacteria         acyltransferase family protein [Burkholderia mallei SAVP1].  
189352676         SIG+10TMAct                                                                                BMULJ_03907              372    Burkholderia multivorans ATCC 17616                                   proteobacteria>betaproteobacteria         putative acyltransferase [Burkholderia multivorans ATCC 17616].  
161521154         SIG+10TMAct                                                                                Bmul_4611                364    Burkholderia multivorans ATCC 17616                                   proteobacteria>betaproteobacteria         acyltransferase 3 [Burkholderia multivorans ATCC 17616].  
186477211         10TMAct                                                                                    Bphy_2462                381    Burkholderia phymatum STM815                                          proteobacteria>betaproteobacteria         acyltransferase 3 [Burkholderia phymatum STM815].  
186475836         10TMAct                                                                                    Bphy_1071                366    Burkholderia phymatum STM815                                          proteobacteria>betaproteobacteria         acyltransferase 3 [Burkholderia phymatum STM815].  
187922834         10TMAct                                                                                    Bphyt_0830               384    Burkholderia phytofirmans PsJN                                        proteobacteria>betaproteobacteria         acyltransferase 3 [Burkholderia phytofirmans PsJN].  
187923957         SIG+10TMAct                                                                                Bphyt_1967               368    Burkholderia phytofirmans PsJN                                        proteobacteria>betaproteobacteria         acyltransferase 3 [Burkholderia phytofirmans PsJN].  
187920720         10TMAct                                                                                    Bphyt_6049               423    Burkholderia phytofirmans PsJN                                        proteobacteria>betaproteobacteria         acyltransferase 3 [Burkholderia phytofirmans PsJN].  
187923416         10TMAct                                                                                    Bphyt_1419               377    Burkholderia phytofirmans PsJN                                        proteobacteria>betaproteobacteria         acyltransferase 3 [Burkholderia phytofirmans PsJN].  
126456100         10TMAct+TM+GDSL                                                                            BURPS1106A_A0375         702    Burkholderia pseudomallei 1106a                                       proteobacteria>betaproteobacteria         putative acyltransferase [Burkholderia pseudomallei 1106a].  
126458413         10TMAct                                                                                    BURPS1106A_A2282         461    Burkholderia pseudomallei 1106a                                       proteobacteria>betaproteobacteria         putative acyltransferase [Burkholderia pseudomallei 1106a].  
126458565         SIG+10TMAct                                                                                BURPS1106A_A2294         352    Burkholderia pseudomallei 1106a                                       proteobacteria>betaproteobacteria         putative acyltransferase [Burkholderia pseudomallei 1106a].  
76817779          SIG+TM+TM+10TMAct                                                                          BURPS1710b_A0747         697    Burkholderia pseudomallei 1710b                                       proteobacteria>betaproteobacteria         acyltransferase family protein [Burkholderia pseudomallei 1710b].  
76819774          SIG+10TMAct                                                                                BURPS1710b_A0757         352    Burkholderia pseudomallei 1710b                                       proteobacteria>betaproteobacteria         acyltransferase family protein [Burkholderia pseudomallei 1710b].  
76817545          SIG+10TMAct+TM+GDSL                                                                        BURPS1710b_A1805         728    Burkholderia pseudomallei 1710b                                       proteobacteria>betaproteobacteria         O-antigen acetylase [Burkholderia pseudomallei 1710b].  
126444682         10TMAct+TM+GDSL                                                                            BURPS668_A0472           702    Burkholderia pseudomallei 668                                         proteobacteria>betaproteobacteria         putative acyltransferase [Burkholderia pseudomallei 668].  
126444068         SIG+10TMAct                                                                                BURPS668_A2433           352    Burkholderia pseudomallei 668                                         proteobacteria>betaproteobacteria         putative acyltransferase [Burkholderia pseudomallei 668].  
126442897         10TMAct                                                                                    BURPS668_A2420           467    Burkholderia pseudomallei 668                                         proteobacteria>betaproteobacteria         putative acyltransferase [Burkholderia pseudomallei 668].  
53721302          10TMAct+TM+GDSL                                                                            BPSS0268                 704    Burkholderia pseudomallei K96243                                      proteobacteria>betaproteobacteria         O-antigen acetylase [Burkholderia pseudomallei K96243].  
53722707          SIG+10TMAct                                                                                BPSS1687                 352    Burkholderia pseudomallei K96243                                      proteobacteria>betaproteobacteria         hypothetical protein BPSS1687 [Burkholderia pseudomallei K96243].  
78062770          SIG+10TMAct                                                                                Bcep18194_B1920          383    Burkholderia sp. 383                                                  proteobacteria>betaproteobacteria         acyltransferase 3 [Burkholderia sp. 383].  
78062961          10TMAct+TM                                                                                 Bcep18194_B2114          354    Burkholderia sp. 383                                                  proteobacteria>betaproteobacteria         acyltransferase 3 [Burkholderia sp. 383].  
78062954          SIG+10TMAct                                                                                Bcep18194_B2107          372    Burkholderia sp. 383                                                  proteobacteria>betaproteobacteria         acyltransferase 3 [Burkholderia sp. 383].  
83717571          SIG+10TMAct+TM+GDSL                                                                        BTH_II2132               760    Burkholderia thailandensis E264                                       proteobacteria>betaproteobacteria         O-antigen acetylase, putative [Burkholderia thailandensis E264].  
83720627          10TMAct+GDSL                                                                               BTH_I1858                636    Burkholderia thailandensis E264                                       proteobacteria>betaproteobacteria         acyltransferase family protein [Burkholderia thailandensis E264].  
83718391          10TMAct                                                                                    BTH_II0691               379    Burkholderia thailandensis E264                                       proteobacteria>betaproteobacteria         acyltransferase family protein [Burkholderia thailandensis E264].  
83718215          SIG+10TMAct                                                                                BTH_II0700               437    Burkholderia thailandensis E264                                       proteobacteria>betaproteobacteria         acyltransferase family protein [Burkholderia thailandensis E264].  
134288015         10TMAct+TM+GDSL                                                                            Bcep1808_7414            641    Burkholderia vietnamiensis G4                                         proteobacteria>betaproteobacteria         acyltransferase 3 [Burkholderia vietnamiensis G4].  
134293174         SIG+10TMAct                                                                                Bcep1808_4480            372    Burkholderia vietnamiensis G4                                         proteobacteria>betaproteobacteria         acyltransferase 3 [Burkholderia vietnamiensis G4].  
134293167         10TMAct                                                                                    Bcep1808_4473            354    Burkholderia vietnamiensis G4                                         proteobacteria>betaproteobacteria         acyltransferase 3 [Burkholderia vietnamiensis G4].  
91782793          10TMAct                                                                                    Bxe_A3032                377    Burkholderia xenovorans LB400                                         proteobacteria>betaproteobacteria         acyltransferase family protein [Burkholderia xenovorans LB400].  
91781990          10TMAct                                                                                    Bxe_A3854                383    Burkholderia xenovorans LB400                                         proteobacteria>betaproteobacteria         putative acyltransferase [Burkholderia xenovorans LB400].  
91783583          SIG+10TMAct+TM                                                                             Bxe_A2232                372    Burkholderia xenovorans LB400                                         proteobacteria>betaproteobacteria         putative membrane-located cell surface O-antigen acetylase  
91777846          SIG+10TMAct                                                                                Bxe_B2282                418    Burkholderia xenovorans LB400                                         proteobacteria>betaproteobacteria         putative acyltransferase [Burkholderia xenovorans LB400].  
160896350         10TMAct                                                                                    Daci_0901                382    Delftia acidovorans SPH-1                                             proteobacteria>betaproteobacteria         acyltransferase 3 [Delftia acidovorans SPH-1].  
152983083         SIG+10TMAct+TM+GDSL                                                                        mma_1708                 484    Janthinobacterium sp. Marseille                                       proteobacteria>betaproteobacteria         hypothetical protein mma_1708 [Janthinobacterium sp. Marseille].  
124267943         10TMAct+TM+GDSL                                                                            Mpe_A2758                691    Methylibium petroleiphilum PM1                                        proteobacteria>betaproteobacteria         O-antigen acetylase [Methylibium petroleiphilum PM1].  
91774790          10TMAct+GDSL                                                                               Mfla_0435                679    Methylobacillus flagellatus KT                                        proteobacteria>betaproteobacteria         acyltransferase 3 [Methylobacillus flagellatus KT].  
59802031          10TMAct+TM+GDSL                                                                            NGO1710                  622    Neisseria gonorrhoeae FA 1090                                         proteobacteria>betaproteobacteria         putative trans-acylase protein [Neisseria gonorrhoeae FA 1090].  
59800529          10TMAct+TM+GDSL                                                                            NGO0065                  624    Neisseria gonorrhoeae FA 1090                                         proteobacteria>betaproteobacteria         putative lipo-oligosaccharide acyltransferase [Neisseria  
194099602         10TMAct+TM+GDSL                                                                            NGK_2108                 622    Neisseria gonorrhoeae NCCP11945                                       proteobacteria>betaproteobacteria         putative trans-acylase protein [Neisseria gonorrhoeae NCCP11945].  
161869379         10TMAct+TM+GDSL                                                                            wbpC                     625    Neisseria meningitidis 053442                                         proteobacteria>betaproteobacteria         lipopolysaccharide biosynthesis protein WbpC, putative [Neisseria  
121634164         10TMAct+TM+GDSL                                                                            NMC0291                  622    Neisseria meningitidis FAM18                                          proteobacteria>betaproteobacteria         putative inner membrane trans-acylase protein [Neisseria  
121634246         10TMAct+TM+GDSL                                                                            NMC0380                  626    Neisseria meningitidis FAM18                                          proteobacteria>betaproteobacteria         putative lipopolysaccharide modification acyltransferase [Neisseria  
15677672          10TMAct+TM+GDSL                                                                            NMB1836                  623    Neisseria meningitidis MC58                                           proteobacteria>betaproteobacteria         putative lipopolysaccharide biosynthesis protein WbpC [Neisseria  
15793609          10TMAct+TM+GDSL                                                                            NMA0619                  622    Neisseria meningitidis Z2491                                          proteobacteria>betaproteobacteria         lipopolysaccharide modification acyltransferase [Neisseria  
15795071          10TMAct+TM+GDSL                                                                            NMA2202                  622    Neisseria meningitidis Z2491                                          proteobacteria>betaproteobacteria         inner membrane trans-acylase protein [Neisseria meningitidis  
145588524         10TMAct+TM+GDSL                                                                            Pnuc_0337                690    Polynucleobacter necessarius subsp. asymbioticus QLW-P1DMWA-1         proteobacteria>betaproteobacteria         acyltransferase 3 [Polynucleobacter necessarius subsp. asymbioticus  
187927727         SIG+10TMAct                                                                                Rpic_0631                360    Ralstonia pickettii 12J                                               proteobacteria>betaproteobacteria         acyltransferase 3 [Ralstonia pickettii 12J].  
89899496          10TMAct+TM+GDSL                                                                            Rfer_0685                658    Rhodoferax ferrireducens T118                                         proteobacteria>betaproteobacteria         acyltransferase 3 [Rhodoferax ferrireducens T118].  
78358595          SIG+10TMAct+TM+GDSL                                                                        Dde_3556                 658    Desulfovibrio desulfuricans subsp. desulfuricans str. G20             proteobacteria>deltaproteobacteria        putative O-antigen acetylase [Desulfovibrio desulfuricans subsp.  
86158491          10TMAct                                                                                    Adeh_2069                376    Anaeromyxobacter dehalogenans 2CP-C                                   proteobacteria>deltaproteobacteria        acyltransferase 3 [Anaeromyxobacter dehalogenans 2CP-C].  
162457240         SIG+10TMAct                                                                                sce8955                  373    Sorangium cellulosum 'So ce 56'                                       proteobacteria>deltaproteobacteria        hypothetical protein sce8955 [Sorangium cellulosum 'So ce 56'].  
108761336         10TMAct                                                                                    MXAN_3238                403    Myxococcus xanthus DK 1622                                            proteobacteria>deltaproteobacteria        acyltransferase family protein [Myxococcus xanthus DK 1622].  
78356288          SIG+10TMAct+TM+GDSL                                                                        Dde_1241                 675    Desulfovibrio desulfuricans subsp. desulfuricans str. G20             proteobacteria>deltaproteobacteria        hypothetical protein Dde_1241 [Desulfovibrio desulfuricans subsp.  
148262776         SIG+10TMAct                                                                                Gura_0699                371    Geobacter uraniireducens Rf4                                          proteobacteria>deltaproteobacteria        acyltransferase 3 [Geobacter uraniireducens Rf4].  
86157587          10TMAct                                                                                    Adeh_1161                365    Anaeromyxobacter dehalogenans 2CP-C                                   proteobacteria>deltaproteobacteria        acyltransferase 3 [Anaeromyxobacter dehalogenans 2CP-C].  
51243869          10TMAct+TM+GDSL                                                                            DP0017                   689    Desulfotalea psychrophila LSv54                                       proteobacteria>deltaproteobacteria        O-antigen acetylase [Desulfotalea psychrophila LSv54].  
108762380         10TMAct                                                                                    MXAN_1736                386    Myxococcus xanthus DK 1622                                            proteobacteria>deltaproteobacteria        acyltransferase family protein [Myxococcus xanthus DK 1622].  
108763526         10TMAct                                                                                    MXAN_7325                410    Myxococcus xanthus DK 1622                                            proteobacteria>deltaproteobacteria        acyltransferase family protein [Myxococcus xanthus DK 1622].  
197122215         SIG+10TMAct+TM                                                                             AnaeK_1808               376    Anaeromyxobacter sp. K                                                proteobacteria>deltaproteobacteria        acyltransferase 3 [Anaeromyxobacter sp. K].  
162457265         SIG+10TMAct+GDSL                                                                           sce8980                  612    Sorangium cellulosum 'So ce 56'                                       proteobacteria>deltaproteobacteria        hypothetical protein sce8980 [Sorangium cellulosum 'So ce 56'].  
157736927         10TMAct+TM+GDSL                                                                            Abu_0676                 668    Arcobacter butzleri RM4018                                            proteobacteria>epsilonproteobacteria      putative O-antigen acyltransferase [Arcobacter butzleri RM4018].  
50084945          SIG+10TMAct+TM+TM+GDSL                                                                     ACIAD1798                597    Acinetobacter sp. ADP1                                                proteobacteria>gammaproteobacteria        putative lipopolysaccharide modification acyltransferase  
110833538         10TMAct+TM+GDSL                                                                            ABO_0677                 597    Alcanivorax borkumensis SK2                                           proteobacteria>gammaproteobacteria        acyltransferase, putative [Alcanivorax borkumensis SK2].  
110833778         10TMAct+GDSL                                                                               ABO_0917                 651    Alcanivorax borkumensis SK2                                           proteobacteria>gammaproteobacteria        acyltransferase, putative [Alcanivorax borkumensis SK2].  
209809835         SIG+10TMAct+TM+GDSL                                                                        VSAL_p54_03              642    Aliivibrio salmonicida LFI1238                                        proteobacteria>gammaproteobacteria        acyltransferase [Aliivibrio salmonicida LFI1238].  
196156105         SIG+10TMAct+TM                                                                             MADE_01286               344    Alteromonas macleodii 'Deep ecotype'                                  proteobacteria>gammaproteobacteria        putative lipopolysaccharide modification acyltransferase  
196157253         10TMAct+TM+GDSL                                                                            MADE_02452               639    Alteromonas macleodii 'Deep ecotype'                                  proteobacteria>gammaproteobacteria        putative lipopolysaccharide modification acyltransferase  
71279655          10TMAct+TM+GDSL                                                                            CPS_5009                 656    Colwellia psychrerythraea 34H                                         proteobacteria>gammaproteobacteria        putative lipopolysaccharide modification acyltransferase [Colwellia  
188534839         10TMAct+GDSL                                                                               ETA_27190                628    Erwinia tasmaniensis Et1/99                                           proteobacteria>gammaproteobacteria        O-antigen acetylase [Erwinia tasmaniensis Et1/99].  
167627533         10TMAct+TM+GDSL                                                                            Fphi_1307                660    Francisella philomiragia subsp. philomiragia ATCC 25017               proteobacteria>gammaproteobacteria        hypothetical protein Fphi_1307 [Francisella philomiragia subsp.  
167627534         10TMAct+TM+GDSL                                                                            Fphi_1308                645    Francisella philomiragia subsp. philomiragia ATCC 25017               proteobacteria>gammaproteobacteria        hypothetical protein Fphi_1308 [Francisella philomiragia subsp.  
167626563         SIG+10TMAct+TM+GDSL                                                                        Fphi_0345                658    Francisella philomiragia subsp. philomiragia ATCC 25017               proteobacteria>gammaproteobacteria        hypothetical protein Fphi_0345 [Francisella philomiragia subsp.  
167627535         10TMAct+TM+GDSL                                                                            Fphi_1309                668    Francisella philomiragia subsp. philomiragia ATCC 25017               proteobacteria>gammaproteobacteria        hypothetical protein Fphi_1309 [Francisella philomiragia subsp.  
68248995          10TMAct+TM+GDSL                                                                            NTHI0512                 622    Haemophilus influenzae 86-028NP                                       proteobacteria>gammaproteobacteria        acyltransferase [Haemophilus influenzae 86-028NP].  
148827594         10TMAct+TM+GDSL                                                                            CGSHiGG_05135            630    Haemophilus influenzae PittGG                                         proteobacteria>gammaproteobacteria        long-chain-fatty-acid--CoA ligase [Haemophilus influenzae PittGG].  
30995371          SIG+10TMAct+TM+GDSL                                                                        HI0391                   336    Haemophilus influenzae Rd KW20                                        proteobacteria>gammaproteobacteria        hypothetical protein HI0391 [Haemophilus influenzae Rd KW20].  
16272341          SIG+10TMAct                                                                                HI0392                   245    Haemophilus influenzae Rd KW20                                        proteobacteria>gammaproteobacteria        O-antigen acetylase [Haemophilus influenzae Rd KW20].  
113460698         10TMAct+TM+GDSL                                                                            HS_0553                  544    Haemophilus somnus 129PT                                              proteobacteria>gammaproteobacteria        acetyltransferase [Haemophilus somnus 129PT].  
170717890         10TMAct                                                                                    HSM_1624                 342    Haemophilus somnus 2336                                               proteobacteria>gammaproteobacteria        acyltransferase 3 [Haemophilus somnus 2336].  
83647620          10TMAct+TM+GDSL                                                                            HCH_04941                625    Hahella chejuensis KCTC 2396                                          proteobacteria>gammaproteobacteria        acyltransferase [Hahella chejuensis KCTC 2396].  
83645507          10TMAct+TM+GDSL                                                                            HCH_02732                660    Hahella chejuensis KCTC 2396                                          proteobacteria>gammaproteobacteria        acyltransferase [Hahella chejuensis KCTC 2396].  
148360566         10TMAct+TM+GDSL                                                                            LPC_2510                 660    Legionella pneumophila str. Corby                                     proteobacteria>gammaproteobacteria        O-antigen acetylase [Legionella pneumophila str. Corby].  
148358666         SIG+10TMAct+TM+GDSL                                                                        LPC_0542                 657    Legionella pneumophila str. Corby                                     proteobacteria>gammaproteobacteria        acyltransferase [Legionella pneumophila str. Corby].  
148361111         SIG+10TMAct+TM+GDSL                                                                        LPC_3085                 658    Legionella pneumophila str. Corby                                     proteobacteria>gammaproteobacteria        O-acetyltransferase [Legionella pneumophila str. Corby].  
54295437          SIG+10TMAct+TM+GDSL                                                                        lpl2523                  657    Legionella pneumophila str. Lens                                      proteobacteria>gammaproteobacteria        hypothetical protein lpl2523 [Legionella pneumophila str. Lens].  
54293765          10TMAct+TM+GDSL                                                                            lpl0821                  660    Legionella pneumophila str. Lens                                      proteobacteria>gammaproteobacteria        hypothetical protein lpl0821 [Legionella pneumophila str. Lens].  
54295627          SIG+10TMAct+TM+GDSL                                                                        lpl2714                  658    Legionella pneumophila str. Lens                                      proteobacteria>gammaproteobacteria        hypothetical protein lpl2714 [Legionella pneumophila str. Lens].  
54298781          SIG+10TMAct+TM+GDSL                                                                        lpp2845                  658    Legionella pneumophila str. Paris                                     proteobacteria>gammaproteobacteria        hypothetical protein lpp2845 [Legionella pneumophila str. Paris].  
54298589          SIG+10TMAct+TM+GDSL                                                                        lpp2653                  657    Legionella pneumophila str. Paris                                     proteobacteria>gammaproteobacteria        hypothetical protein lpp2653 [Legionella pneumophila str. Paris].  
54296806          10TMAct+TM+GDSL                                                                            lpp0846                  660    Legionella pneumophila str. Paris                                     proteobacteria>gammaproteobacteria        hypothetical protein lpp0846 [Legionella pneumophila str. Paris].  
52842806          SIG+10TMAct+TM+GDSL                                                                        lpg2600                  657    Legionella pneumophila subsp. pneumophila str. Philadelphia 1         proteobacteria>gammaproteobacteria        acyltransferase [Legionella pneumophila subsp. pneumophila str.  
52842995          SIG+10TMAct+TM+GDSL                                                                        lpg2799                  658    Legionella pneumophila subsp. pneumophila str. Philadelphia 1         proteobacteria>gammaproteobacteria        O-acetyltransferase [Legionella pneumophila subsp. pneumophila str.  
120554574         SIG+10TMAct                                                                                Maqu_1654                380    Marinobacter aquaeolei VT8                                            proteobacteria>gammaproteobacteria        acyltransferase 3 [Marinobacter aquaeolei VT8].  
53804678          10TMAct+TM+GDSL                                                                            MCA0963                  759    Methylococcus capsulatus str. Bath                                    proteobacteria>gammaproteobacteria        acyltransferase family protein [Methylococcus capsulatus str.  
197286800         10TMAct+TM+GDSL                                                                            PMI2971                  659    Proteus mirabilis HI4320                                              proteobacteria>gammaproteobacteria        surface polysaccharide modification acyltransferase [Proteus  
109897421         SIG+10TMAct+TM+GDSL                                                                        Patl_1096                614    Pseudoalteromonas atlantica T6c                                       proteobacteria>gammaproteobacteria        acyltransferase 3 [Pseudoalteromonas atlantica T6c].  
109897409         SIG+10TMAct+TM+GDSL                                                                        Patl_1084                635    Pseudoalteromonas atlantica T6c                                       proteobacteria>gammaproteobacteria        acyltransferase 3 [Pseudoalteromonas atlantica T6c].  
77359372          10TMAct+TM+GDSL                                                                            PSHAa0406                522    Pseudoalteromonas haloplanktis TAC125                                 proteobacteria>gammaproteobacteria        hypothetical protein PSHAa0406 [Pseudoalteromonas haloplanktis  
152984265         10TMAct+TM+GDSL                                                                            PSPA7_5981               662    Pseudomonas aeruginosa PA7                                            proteobacteria>gammaproteobacteria        putative O-antigen acetylase [Pseudomonas aeruginosa PA7].  
15598353          SIG+10TMAct+GDSL                                                                           PA3157                   629    Pseudomonas aeruginosa PAO1                                           proteobacteria>gammaproteobacteria        acetyltransferase [Pseudomonas aeruginosa PAO1].  
15600431          SIG+10TMAct+TM+GDSL                                                                        PA5238                   662    Pseudomonas aeruginosa PAO1                                           proteobacteria>gammaproteobacteria        putative O-antigen acetylase [Pseudomonas aeruginosa PAO1].  
116053385         SIG+10TMAct+TM+GDSL                                                                        PA14_69170               662    Pseudomonas aeruginosa UCBPP-PA14                                     proteobacteria>gammaproteobacteria        putative O-antigen acetylase [Pseudomonas aeruginosa UCBPP-PA14].  
77461676          10TMAct+TM+GDSL                                                                            Pfl01_5455               665    Pseudomonas fluorescens Pf0-1                                         proteobacteria>gammaproteobacteria        acyltransferase 3 [Pseudomonas fluorescens Pf0-1].  
77458835          10TMAct+GDSL                                                                               Pfl01_2610               555    Pseudomonas fluorescens Pf0-1                                         proteobacteria>gammaproteobacteria        acyltransferase 3 [Pseudomonas fluorescens Pf0-1].  
167032770         10TMAct+TM+GDSL                                                                            PputGB1_1762             662    Pseudomonas putida GB-1                                               proteobacteria>gammaproteobacteria        acyltransferase 3 [Pseudomonas putida GB-1].  
71736143          10TMAct+TM+GDSL                                                                            PSPPH_5170               672    Pseudomonas syringae pv. phaseolicola 1448A                           proteobacteria>gammaproteobacteria        acyltransferase family protein [Pseudomonas syringae pv.  
71736798          10TMAct+TM                                                                                 PSPPH_0896               408    Pseudomonas syringae pv. phaseolicola 1448A                           proteobacteria>gammaproteobacteria        acyltransferase family protein [Pseudomonas syringae pv.  
66048311          10TMAct+TM+GDSL                                                                            Psyr_5087                627    Pseudomonas syringae pv. syringae B728a                               proteobacteria>gammaproteobacteria        acyltransferase 3 [Pseudomonas syringae pv. syringae B728a].  
66044117          10TMAct+GDSL                                                                               Psyr_0862                673    Pseudomonas syringae pv. syringae B728a                               proteobacteria>gammaproteobacteria        acyltransferase 3 [Pseudomonas syringae pv. syringae B728a].  
28872652          SIG+10TMAct+TM+GDSL                                                                        PSPTO_5547               628    Pseudomonas syringae pv. tomato str. DC3000                           proteobacteria>gammaproteobacteria        acyltransferase family protein [Pseudomonas syringae pv. tomato  
28868217          10TMAct+GDSL                                                                               PSPTO_0997               667    Pseudomonas syringae pv. tomato str. DC3000                           proteobacteria>gammaproteobacteria        acyltransferase family protein [Pseudomonas syringae pv. tomato  
71065204          SIG+10TMAct+TM+GDSL                                                                        Psyc_0638                647    Psychrobacter arcticus 273-4                                          proteobacteria>gammaproteobacteria        acyltransferase family protein [Psychrobacter arcticus 273-4].  
119944221         10TMAct+TM+GDSL                                                                            Ping_0443                625    Psychromonas ingrahamii 37                                            proteobacteria>gammaproteobacteria        acyltransferase 3 [Psychromonas ingrahamii 37].  
90019823          10TMAct                                                                                    Sde_0174                 358    Saccharophagus degradans 2-40                                         proteobacteria>gammaproteobacteria        acyltransferase-like [Saccharophagus degradans 2-40].  
29140985          10TMAct+TM+GDSL                                                                            t0466                    640    Salmonella enterica subsp. enterica serovar Typhi str. Ty2            proteobacteria>gammaproteobacteria        putative lipopolysaccharide modification acyltransferase  
161613060         SIG+10TMAct+TM+GDSL                                                                        SPAB_00767               609    Salmonella enterica subsp. enterica serovar Paratyphi B str. SPB7     proteobacteria>gammaproteobacteria        hypothetical protein SPAB_00767 [Salmonella enterica subsp.  
16761319          10TMAct+TM+GDSL                                                                            STY2629                  640    Salmonella enterica subsp. enterica serovar Typhi str. CT18           proteobacteria>gammaproteobacteria        putative lipopolysaccharide modification acyltransferase  
205353508         10TMAct+TM+GDSL                                                                            gtrC                     640    Salmonella enterica subsp. enterica serovar Gallinarum str. 287/91    proteobacteria>gammaproteobacteria        putative lipopolysaccharide modification glycosyltransferase  
56412707          10TMAct+TM+GDSL                                                                            SPA0467                  640    Salmonella enterica subsp. enterica serovar Paratyphi A str. ATCC     proteobacteria>gammaproteobacteria        putative lipopolysaccharide modification acyltransferase  
198244472         10TMAct+TM+GDSL                                                                            SeD_A2751                640    Salmonella enterica subsp. enterica serovar Dublin str. CT_02021853   proteobacteria>gammaproteobacteria        acyltransferase family protein [Salmonella enterica subsp. enterica  
16765560          SIG+10TMAct+TM+GDSL                                                                        oafA                     609    Salmonella enterica subsp. enterica serovar Typhimurium str. LT2      proteobacteria>gammaproteobacteria        O-antigen acetylase [Salmonella typhimurium LT2].  
207857813         10TMAct+TM+GDSL                                                                            gtrC                     640    Salmonella enterica subsp. enterica serovar Enteritidis str.          proteobacteria>gammaproteobacteria        putative lipopolysaccharide modification acyltransferase  
91792040          SIG+10TMAct                                                                                Sden_0678                379    Shewanella denitrificans OS217                                        proteobacteria>gammaproteobacteria        acyltransferase 3 [Shewanella denitrificans OS217].  
170724439         SIG+10TMAct+TM+GDSL                                                                        Swoo_0069                652    Shewanella woodyi ATCC 51908                                          proteobacteria>gammaproteobacteria        acyltransferase 3 [Shewanella woodyi ATCC 51908].  
170725916         10TMAct+TM+GDSL                                                                            Swoo_1561                643    Shewanella woodyi ATCC 51908                                          proteobacteria>gammaproteobacteria        acyltransferase 3 [Shewanella woodyi ATCC 51908].  
190572785         10TMAct+TM+GDSL                                                                            Smlt0737                 666    Stenotrophomonas maltophilia K279a                                    proteobacteria>gammaproteobacteria        putative O-antigen acetylase [Stenotrophomonas maltophilia K279a].  
156976861         SIG+10TMAct+TM+GDSL                                                                        VIBHAR_05637             615    Vibrio harveyi ATCC BAA-1116                                          proteobacteria>gammaproteobacteria        acyltransferase [Vibrio harveyi ATCC BAA-1116].  
28900626          SIG+10TMAct+TM+GDSL                                                                        VPA0771                  616    Vibrio parahaemolyticus RIMD 2210633                                  proteobacteria>gammaproteobacteria        putative lipopolysaccharide modification acyltransferase [Vibrio  
37678491          10TMAct                                                                                    VV0307                   313    Vibrio vulnificus YJ016                                               proteobacteria>gammaproteobacteria        acyltransferase family protein [Vibrio vulnificus YJ016].  
21244639          10TMAct                                                                                    oac                      399    Xanthomonas axonopodis pv. citri str. 306                             proteobacteria>gammaproteobacteria        acyltransferase [Xanthomonas axonopodis pv. citri str. 306].  
188993444         SIG+10TMAct                                                                                xccb100_4049             374    Xanthomonas campestris pv. campestris str. B100                       proteobacteria>gammaproteobacteria        Putative acyltransferase [Xanthomonas campestris pv. campestris  
66770246          SIG+10TMAct                                                                                XC_3949                  374    Xanthomonas campestris pv. campestris str. 8004                       proteobacteria>gammaproteobacteria        acyltransferase [Xanthomonas campestris pv. campestris str. 8004].  
78049589          10TMAct                                                                                    XCV4033                  342    Xanthomonas campestris pv. vesicatoria str. 85-10                     proteobacteria>gammaproteobacteria        putative acyltransferase [Xanthomonas campestris pv. vesicatoria  
15837380          10TMAct+GDSL                                                                               XF0778                   706    Xylella fastidiosa 9a5c                                               proteobacteria>gammaproteobacteria        O-antigen acetylase [Xylella fastidiosa 9a5c].  
45658201          10TMAct                                                                                    yrhL                     403    Leptospira interrogans serovar Copenhageni str. Fiocruz L1-130        spirochaetes                              cell surface saccharide acetylase protein [Leptospira interrogans  
183221424         SIG+10TMAct+TM+GDSL                                                                        LEPBI_I2042              698    Leptospira biflexa serovar Patoc strain 'Patoc 1 (Paris)'             spirochaetes                              putative acyltransferase; putative membrane protein [Leptospira  
116327489         10TMAct                                                                                    LBL_0713                 408    Leptospira borgpetersenii serovar Hardjo-bovis L550                   spirochaetes                              acyltransferase [Leptospira borgpetersenii serovar Hardjo-bovis  
116331897         10TMAct                                                                                    LBJ_2395                 408    Leptospira borgpetersenii serovar Hardjo-bovis JB197                  spirochaetes                              acyltransferase [Leptospira borgpetersenii serovar Hardjo-bovis  
24213028          10TMAct                                                                                    LA0328                   408    Leptospira interrogans serovar Lai str. 56601                         spirochaetes                              acyltransferase [Leptospira interrogans serovar Lai str. 56601].  
45656189          10TMAct                                                                                    LIC10285                 408    Leptospira interrogans serovar Copenhageni str. Fiocruz L1-130        spirochaetes                              putativeacetyltransferase [Leptospira interrogans serovar  
183219885         10TMAct                                                                                    LEPBI_I0467              403    Leptospira biflexa serovar Patoc strain 'Patoc 1 (Paris)'             spirochaetes                              putative acyltransferase; putative membrane protein [Leptospira  
Aano1000010852    TM+TM+TM+10TMAct+TM                                                                        Aano1000010852           685    Aureococcus anophagefferens                                           stramenopiles                             69078  
  
  
--                -                                                                                          -  
# 209;  beltless ; NRF + Acyltranf  
89307436          TM+10TMAct                                                                                 TTHERM_01078020          723    Tetrahymena thermophila SB210                                         alveolata>ciliophora                      Acyltransferase family protein [Tetrahymena thermophila SB210].  
145496784         SIG+TM+10TMAct                                                                             GSPATT00036116001        715    Paramecium tetraurelia strain d4-2                                    alveolata>ciliophora                      hypothetical protein [Paramecium tetraurelia strain d4-2].  
89305662          SIG+TM+10TMAct                                                                             TTHERM_00473120          712    Tetrahymena thermophila SB210                                         alveolata>ciliophora                      Acyltransferase family protein [Tetrahymena thermophila SB210].  
145527824         TM+10TMAct                                                                                 GSPATT00002444001        587    Paramecium tetraurelia strain d4-2                                    alveolata>ciliophora                      hypothetical protein [Paramecium tetraurelia strain d4-2].  
145530772         TM+10TMAct                                                                                 GSPATT00018078001        607    Paramecium tetraurelia strain d4-2                                    alveolata>ciliophora                      hypothetical protein [Paramecium tetraurelia strain d4-2].  
145497977         SIG+TM+10TMAct                                                                             GSPATT00006887001        659    Paramecium tetraurelia strain d4-2                                    alveolata>ciliophora                      hypothetical protein [Paramecium tetraurelia strain d4-2].  
145527826         SIG+TM+10TMAct                                                                             GSPATT00002445001        659    Paramecium tetraurelia strain d4-2                                    alveolata>ciliophora                      hypothetical protein [Paramecium tetraurelia strain d4-2].  
89285709          SIG+TM+10TMAct                                                                             TTHERM_00825130          858    Tetrahymena thermophila SB210                                         alveolata>ciliophora                      Acyltransferase family protein [Tetrahymena thermophila SB210].  
145475063         SIG+TM+10TMAct                                                                             GSPATT00004250001        656    Paramecium tetraurelia strain d4-2                                    alveolata>ciliophora                      hypothetical protein [Paramecium tetraurelia strain d4-2].  
145478599         SIG+TM+10TMAct                                                                             GSPATT00028959001        650    Paramecium tetraurelia strain d4-2                                    alveolata>ciliophora                      hypothetical protein [Paramecium tetraurelia strain d4-2].  
145497759         TM+10TMAct                                                                                 GSPATT00006777001        607    Paramecium tetraurelia strain d4-2                                    alveolata>ciliophora                      hypothetical protein [Paramecium tetraurelia strain d4-2].  
89294168          TM+10TMAct                                                                                 TTHERM_00803530          614    Tetrahymena thermophila SB210                                         alveolata>ciliophora                      Acyltransferase family protein [Tetrahymena thermophila SB210].  
118359517         SIG+TM+10TMAct                                                                             TTHERM_00322970          679    Tetrahymena thermophila                                               alveolata>ciliophora                      Acyltransferase family protein [Tetrahymena thermophila].  
118388734         TM+10TMAct                                                                                 TTHERM_00647540          815    Tetrahymena thermophila                                               alveolata>ciliophora                      Acyltransferase family protein [Tetrahymena thermophila].  
145489189         SIG+TM+10TMAct                                                                             GSPATT00033052001        716    Paramecium tetraurelia strain d4-2                                    alveolata>ciliophora                      hypothetical protein [Paramecium tetraurelia strain d4-2].  
Dpur1000011142    SIG+TM+10TMAct                                                                             Dpur1000011142           780    Dictyostelium purpureum                                               amoebozoa>mycetozoa                       GID1.0049545  
167537822         SIG+TM+10TMAct                                                                             MONBRDRAFT_12668         953    Monosiga brevicollis MX1                                              choanoflagellida                          hypothetical protein [Monosiga brevicollis MX1].  
167536497         SIG+TM+10TMAct                                                                             MONBRDRAFT_38918         699    Monosiga brevicollis MX1                                              choanoflagellida                          hypothetical protein [Monosiga brevicollis MX1].  
219493433         SIG+TM+10TMAct                                                                             BRAFLDRAFT_105715        684    Branchiostoma floridae                                                metazoa                                   hypothetical protein BRAFLDRAFT_105715 [Branchiostoma floridae].  
210088713         SIG+10TMAct                                                                                BRAFLDRAFT_105716        640    Branchiostoma floridae                                                metazoa                                   hypothetical protein BRAFLDRAFT_105716 [Branchiostoma floridae].  
210109917         10TMAct                                                                                    BRAFLDRAFT_85037         644    Branchiostoma floridae                                                metazoa                                   hypothetical protein BRAFLDRAFT_85037 [Branchiostoma floridae].  
210084705         SIG+10TMAct                                                                                BRAFLDRAFT_109419        164    Branchiostoma floridae                                                metazoa                                   hypothetical protein BRAFLDRAFT_109419 [Branchiostoma floridae].  
210092350         SIG+TM+10TMAct                                                                             BRAFLDRAFT_102246        607    Branchiostoma floridae                                                metazoa                                   hypothetical protein BRAFLDRAFT_102246 [Branchiostoma floridae].  
210103576         SIG+10TMAct                                                                                BRAFLDRAFT_91501         595    Branchiostoma floridae                                                metazoa                                   hypothetical protein BRAFLDRAFT_91501 [Branchiostoma floridae].  
219484216         SIG+10TMAct                                                                                BRAFLDRAFT_101594        349    Branchiostoma floridae                                                metazoa                                   hypothetical protein BRAFLDRAFT_101594 [Branchiostoma floridae].  
210113364         SIG+10TMAct+IPP                                                                            BRAFLDRAFT_81521         621    Branchiostoma floridae                                                metazoa                                   hypothetical protein BRAFLDRAFT_81521 [Branchiostoma floridae].  
219429881         10TMAct                                                                                    BRAFLDRAFT_75116         330    Branchiostoma floridae                                                metazoa                                   hypothetical protein BRAFLDRAFT_75116 [Branchiostoma floridae].  
198421178         SIG+TM+10TMAct                                                                             LOC100181555             762    Ciona intestinalis                                                    metazoa                                   PREDICTED: similar to CG5892 CG5892-PA [Ciona intestinalis].  
219452974         SIG+10TMAct                                                                                BRAFLDRAFT_124632        585    Branchiostoma floridae                                                metazoa                                   hypothetical protein BRAFLDRAFT_124632 [Branchiostoma floridae].  
219443144         SIG+10TMAct                                                                                BRAFLDRAFT_81523         577    Branchiostoma floridae                                                metazoa                                   hypothetical protein BRAFLDRAFT_81523 [Branchiostoma floridae].  
219460994         SIG+TM+10TMAct                                                                             BRAFLDRAFT_90701         372    Branchiostoma floridae                                                metazoa                                   hypothetical protein BRAFLDRAFT_90701 [Branchiostoma floridae].  
210111815         10TMAct                                                                                    BRAFLDRAFT_83078         374    Branchiostoma floridae                                                metazoa                                   hypothetical protein BRAFLDRAFT_83078 [Branchiostoma floridae].  
219450105         TM+10TMAct                                                                                 BRAFLDRAFT_85033         555    Branchiostoma floridae                                                metazoa                                   hypothetical protein BRAFLDRAFT_85033 [Branchiostoma floridae].  
219412921         SIG+10TMAct                                                                                BRAFLDRAFT_66615         542    Branchiostoma floridae                                                metazoa                                   hypothetical protein BRAFLDRAFT_66615 [Branchiostoma floridae].  
210106850         SIG+TM+10TMAct                                                                             BRAFLDRAFT_88115         527    Branchiostoma floridae                                                metazoa                                   hypothetical protein BRAFLDRAFT_88115 [Branchiostoma floridae].  
210078888         10TMAct                                                                                    BRAFLDRAFT_113331        155    Branchiostoma floridae                                                metazoa                                   hypothetical protein BRAFLDRAFT_113331 [Branchiostoma floridae].  
210088529         10TMAct+CALC                                                                               BRAFLDRAFT_105902        983    Branchiostoma floridae                                                metazoa                                   hypothetical protein BRAFLDRAFT_105902 [Branchiostoma floridae].  
210107905         10TMAct                                                                                    BRAFLDRAFT_86997         511    Branchiostoma floridae                                                metazoa                                   hypothetical protein BRAFLDRAFT_86997 [Branchiostoma floridae].  
219443244         SIG+TM+10TMAct                                                                             BRAFLDRAFT_81522         628    Branchiostoma floridae                                                metazoa                                   hypothetical protein BRAFLDRAFT_81522 [Branchiostoma floridae].  
198420343         SIG+TM+10TMAct                                                                             LOC100184946             498    Ciona intestinalis                                                    metazoa                                   PREDICTED: similar to predicted protein, partial [Ciona  
210109916         TM+10TMAct                                                                                 BRAFLDRAFT_85035         385    Branchiostoma floridae                                                metazoa                                   hypothetical protein BRAFLDRAFT_85035 [Branchiostoma floridae].  
219501827         SIG+TM+10TMAct                                                                             BRAFLDRAFT_132370        719    Branchiostoma floridae                                                metazoa                                   hypothetical protein BRAFLDRAFT_132370 [Branchiostoma floridae].  
210101631         SIG+TM+10TMAct                                                                             BRAFLDRAFT_127041        720    Branchiostoma floridae                                                metazoa                                   hypothetical protein BRAFLDRAFT_127041 [Branchiostoma floridae].  
210088527         SIG+TM+10TMAct                                                                             BRAFLDRAFT_105897        726    Branchiostoma floridae                                                metazoa                                   hypothetical protein BRAFLDRAFT_105897 [Branchiostoma floridae].  
210103534         TM+10TMAct+TM+10TMAct                                                                     BRAFLDRAFT_126287        1366   Branchiostoma floridae                                                metazoa                                   hypothetical protein BRAFLDRAFT_126287 [Branchiostoma floridae].  
210128640         SIG+TM+10TMAct                                                                             BRAFLDRAFT_66621         724    Branchiostoma floridae                                                metazoa                                   hypothetical protein BRAFLDRAFT_66621 [Branchiostoma floridae].  
219417366         TM+TM+10TMAct                                                                              BRAFLDRAFT_69060         662    Branchiostoma floridae                                                metazoa                                   hypothetical protein BRAFLDRAFT_69060 [Branchiostoma floridae].  
Caps1000012212    TM+10TMAct                                                                                 Caps1000012212           756    Capitella spI                                                         metazoa>annelida                          estExt_fgenesh1_pg.C_6110013  
Caps1000016088    SIG+10TMAct                                                                                Caps1000016088           590    Capitella spI                                                         metazoa>annelida                          estExt_fgenesh1_pg.C_2800009  
Caps1000020137    TM+10TMAct                                                                                 Caps1000020137           193    Capitella spI                                                         metazoa>annelida                          gw1.519.57.1  
Caps1000023313    SIG+10TMAct                                                                                Caps1000023313           844    Capitella spI                                                         metazoa>annelida                          fgenesh1_pg.C_scaffold_67000058  
Caps1000012299    SIG+10TMAct                                                                                Caps1000012299           598    Capitella spI                                                         metazoa>annelida                          fgenesh1_pg.C_scaffold_308000008  
Caps1000020409    SIG+10TMAct                                                                                Caps1000020409           240    Capitella spI                                                         metazoa>annelida                          fgenesh1_pg.C_scaffold_8285000002  
Caps1000028542    10TMAct                                                                                    Caps1000028542           516    Capitella spI                                                         metazoa>annelida                          fgenesh1_pg.C_scaffold_9891000001  
Caps1000024206    SIG+TM+10TMAct                                                                             Caps1000024206           720    Capitella spI                                                         metazoa>annelida                          estExt_fgenesh1_pg.C_8760005  
Caps1000009734    SIG+10TMAct                                                                                Caps1000009734           379    Capitella spI                                                         metazoa>annelida                          estExt_Genewise1.C_6680029  
Caps1000001800    TM+10TMAct                                                                                 Caps1000001800           737    Capitella spI                                                         metazoa>annelida                          fgenesh1_pg.C_scaffold_56000053  
Caps1000012211    SIG+10TMAct                                                                                Caps1000012211           352    Capitella spI                                                         metazoa>annelida                          fgenesh1_pg.C_scaffold_611000012  
Caps1000011431    SIG+TM+10TMAct                                                                             Caps1000011431           705    Capitella spI                                                         metazoa>annelida                          fgenesh1_pg.C_scaffold_171000051  
221117776         10TMAct                                                                                    LOC100205697             809    Hydra magnipapillata                                                  metazoa>cnidaria                          PREDICTED: similar to predicted protein [Hydra magnipapillata].  
221129071         10TMAct                                                                                    LOC100204488             305    Hydra magnipapillata                                                  metazoa>cnidaria                          PREDICTED: similar to predicted protein, partial [Hydra  
221117165         SIG+TM+TM+10TMAct                                                                          LOC100211727             639    Hydra magnipapillata                                                  metazoa>cnidaria                          PREDICTED: similar to predicted protein [Hydra magnipapillata].  
221132141         SIG+10TMAct                                                                                LOC100197759             521    Hydra magnipapillata                                                  metazoa>cnidaria                          PREDICTED: similar to predicted protein [Hydra magnipapillata].  
156226523         10TMAct                                                                                    NEMVEDRAFT_v1g86778      440    Nematostella vectensis                                                metazoa>cnidaria                          predicted protein [Nematostella vectensis].  
156401739         SIG+10TMAct                                                                                NEMVEDRAFT_v1g160622     439    Nematostella vectensis                                                metazoa>cnidaria                          predicted protein [Nematostella vectensis].  
156226575         SIG+10TMAct                                                                                NEMVEDRAFT_v1g86704      440    Nematostella vectensis                                                metazoa>cnidaria                          predicted protein [Nematostella vectensis].  
221123729         10TMAct                                                                                    LOC100208537             597    Hydra magnipapillata                                                  metazoa>cnidaria                          PREDICTED: similar to predicted protein [Hydra magnipapillata].  
Dpul1000012049    TM+10TMAct                                                                                 Dpul1000012049           801    Daphnia pulex                                                         metazoa>crustacea                         SNAP_00005860  
Dpul1000009652    SIG+10TMAct                                                                                Dpul1000009652           404    Daphnia pulex                                                         metazoa>crustacea                         NCBI_GNO_1500200  
Dpul1000009637    SIG+TM+10TMAct                                                                             Dpul1000009637           750    Daphnia pulex                                                         metazoa>crustacea                         PASA_GEN_1500180  
Dpul1000009002    SIG+TM+10TMAct                                                                             Dpul1000009002           710    Daphnia pulex                                                         metazoa>crustacea                         fgenesh1_pg.C_scaffold_24000144  
Dpul1000011870    SIG+TM+10TMAct                                                                             Dpul1000011870           749    Daphnia pulex                                                         metazoa>crustacea                         NCBI_GNO_1000061  
Dpul1000009633    10TMAct                                                                                    Dpul1000009633           447    Daphnia pulex                                                         metazoa>crustacea                         e_gw1.15.46.1  
Dpul1000009645    SIG+TM+10TMAct                                                                             Dpul1000009645           765    Daphnia pulex                                                         metazoa>crustacea                         NCBI_GNO_1500193  
Dpul1000011868    SIG+10TMAct                                                                                Dpul1000011868           372    Daphnia pulex                                                         metazoa>crustacea                         estExt_Genewise1.C_100110  
Dpul1000009001    SIG+10TMAct                                                                                Dpul1000009001           377    Daphnia pulex                                                         metazoa>crustacea                         estExt_Genewise1Plus.C_240219  
Dpul1000009624    SIG+10TMAct                                                                                Dpul1000009624           366    Daphnia pulex                                                         metazoa>crustacea                         e_gw1.15.79.1  
Dpul1000022463    SIG+10TMAct                                                                                Dpul1000022463           379    Daphnia pulex                                                         metazoa>crustacea                         e_gw1.91.5.1  
Dpul1000020588    SIG+10TMAct                                                                                Dpul1000020588           382    Daphnia pulex                                                         metazoa>crustacea                         e_gw1.94.9.1  
Dpul1000011872    SIG+10TMAct                                                                                Dpul1000011872           355    Daphnia pulex                                                         metazoa>crustacea                         e_gw1.10.68.1  
Dpul1000023079    SIG+10TMAct                                                                                Dpul1000023079           355    Daphnia pulex                                                         metazoa>crustacea                         gw1.96.8.1  
Dpul1000005544    10TMAct                                                                                    Dpul1000005544           383    Daphnia pulex                                                         metazoa>crustacea                         PASA_GEN_4600080
[truncated: 626,205 more chars]
